# Structure-based discovery of highly bioavailable, covalent, broad-spectrum coronavirus M^Pro^ inhibitors with potent in vivo efficacy

**DOI:** 10.1126/sciadv.adt7836

**Published:** 2025-04-23

**Authors:** Tyler C. Detomasi, Gilles Degotte, Sijie Huang, Rahul K. Suryawanshi, Amy Diallo, Luca Lizzadro, Francisco J. Zaptero-Belinchón, Taha Y. Taha, Jiapeng Li, Alicia L. Richards, Eric R. Hantz, Zain Alam, Mauricio Montano, Maria McCavitt-Malvido, Rajesh Gumpena, James R. Partridge, Galen J. Correy, Yusuke Matsui, Annemarie F. Charvat, Isabella S. Glenn, Julia Rosecrans, Jezrael L. Revalde, Dashiell Anderson, Judd F. Hultquist, Michelle R. Arkin, R. Jeffrey Neitz, Danielle L. Swaney, Nevan J. Krogan, Brian K. Shoichet, Kliment A. Verba, Melanie Ott, Adam R. Renslo, Charles S. Craik

**Affiliations:** ^1^ Department of Pharmaceutical Chemistry, University of California, San Francisco, San Francisco, CA, USA.; ^2^ Gladstone Institute of Virology, Gladstone Institutes, San Francisco, CA, USA.; ^3^ Department of Cellular and Molecular Pharmacology, University of California, San Francisco, San Francisco, CA, USA.; ^4^ Gladstone Institute of Data Science and Biotechnology, Gladstone Institutes, San Francisco, CA, USA.; ^5^ Department of Bioengineering and Therapeutic Sciences, University of California, San Francisco, San Francisco, CA, USA.; ^6^ Quantitative Biosciences Institute (QBI), University of California, San Francisco, San Francisco, CA, USA.; ^7^ Division of Infectious Diseases, Northwestern University Feinberg School of Medicine, Chicago, IL, USA.

## Abstract

The main protease (M^Pro^) of severe acute respiratory syndrome coronavirus 2 (SARS-CoV-2) is a validated drug target. Starting with a lead-like dihydrouracil chemotype identified in a large-library docking campaign, we improved M^Pro^ inhibition >1000-fold by engaging additional M^Pro^ subsites and using a latent electrophile to engage Cys^145^. Advanced leads from this series show pan-coronavirus antiviral activity, low clearance in mice, and for **AVI-4773**, a rapid reduction in viral titers >1,000,000 after just three doses. Both compounds are well distributed in mouse tissues, including brain, where concentrations >1000× the 90% effective concentration are observed 8 hours after oral dosing for **AVI-4773**. **AVI-4516** shows minimal inhibition of major cytochrome P450s and human proteases. **AVI-4516** also exhibits synergy with the RNA-dependent RNA polymerase inhibitor, molnupiravir, in cellular infection models. Related analogs strongly inhibit nirmatrelvir-resistant M^Pro^ mutant virus. The properties of this chemotype are differentiated from existing clinical and preclinical M^Pro^ inhibitors and will advance therapeutic development against emerging SARS-CoV-2 variants and other coronaviruses.

## Teaser

This study describes a class of potent, bioavailable SARS-CoV-2 M^Pro^ inhibitors having an unusual mechanism of inhibition.

## INTRODUCTION

Five years after the start of the COVID-19 pandemic, the persistent threat of highly transmissible, pathogenic, and immune-evading severe acute respiratory syndrome coronavirus 2 (SARS-CoV-2) variants remains a global concern. SARS-CoV-2 variants are expected to continue emerging, and thus, to stop the cycle of infections and emergence of new variants, it is crucial to develop effective direct-acting antiviral therapeutics. Proteolytic processing of the SARS-CoV-2 polyprotein is essential for viral replication and depends on the action of *nsp5* (*1*, *2*), which encodes the main protease (M^Pro^), also referred to as 3C-like protease (3CL^Pro^). Targeting the proteases involved in viral replication has a long track record of success in delivering antiviral therapeutics (*3*). M^Pro^ is a clinically validated target for SARS-CoV-2, with the M^Pro^ inhibitors nirmatrelvir (*4*) and ensitrelvir (*5*) used clinically to treat COVID-19. SARS-CoV-2 will continue to mutate and generate new resistant variants, which calls for new agents with pan-coronavirus activity and enhanced antiviral spectrum to treat these infections. Moreover, given the ongoing risk of future pandemics arising from coronaviral reservoirs in bats and in other small mammals (*6*, *7*), it is crucial to identify molecules that target evolutionarily conserved domains of M^Pro^ to elicit pan coronaviral antiviral activity. This approach is an essential preparative measure beyond the current pandemic (*8*).

Herein, we describe the discovery of uracil-based, nonpeptidic M^Pro^ inhibitors exemplified by the advanced lead molecule **AVI-4516**. Attractive features of this new chemotype include a simple achiral and easily diversified chemical scaffold that exhibits potent biochemical and in vitro antiviral activity against multiple SARS-CoV-2 variants as well as other known human coronaviruses. The most efficacious analogs from this series use an unactivated (latent-electrophilic) alkyne warhead to engage the active site cysteine (Cys^145^) of M^Pro^, leading to potent, irreversible inhibition, both in vitro and in vivo. By virtue of its weak electrophilicity, cross-reactivity with important mammalian proteases is avoided, as are interactions with other important off-targets, such as receptors and ion channels, including the human Ether-a-go-go-Related Gene (hERG) channel. We propose that the desired reactivity with Cys^145^ is promoted by precise positioning of the alkyne function adjacent to the oxyanion hole in M^Pro^ (*9*), thereby stabilizing the developing negative charge (*9*) in the transition state of nucleophilic attack. Overall, advanced leads **AVI-4516** and **AVI-4773** manifest many differentiated properties that augur for the discovery of development candidates for SARS-CoV-2 and related coronaviruses from this new chemotype.

## RESULTS

### Docking campaign reveals dihydrouracil core

**Fig. 1.**
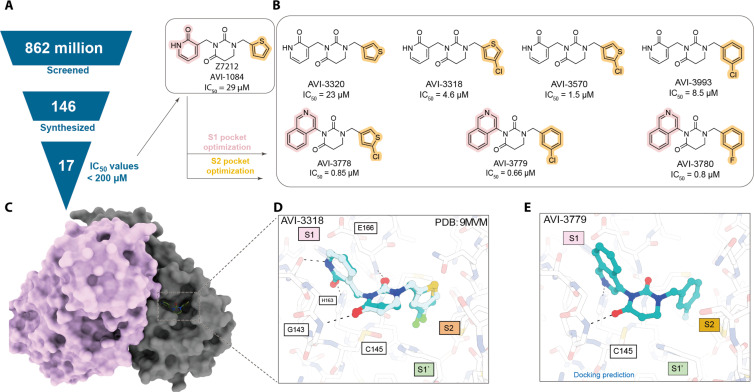
Initial discovery and early structure-based optimization of the AVI-4673 series. (**A**) Large-library docking led to 17 diverse inhibitors, of which Z7212 (**AVI-1084**) is shown (*10*). (**B**) Structure-based optimization of one of them Z7212, explored side chains modeled to bind in the S2 and S1 pockets, leading to submicromolar inhibitors. (**C**) Surface representation of the homodimer of M^Pro^. Protomer A is in pink and protomer B is in light brown. (**D**) Superposition between the docking predicted (green carbons) and the crystal structure (gray carbons, PDB: 9MVM) of the inhibitor **AVI-3318** [from (A)]. (**E**) Docked pose of **AVI-3779**, the most potent inhibitor based on docking.

As we described previously (*10*), an initial docking screen of 862 million “tangible,” make-on-demand molecules against a deposited [Protein Data Bank (PDB): 6Y2G] M^Pro^ structure returned several scaffolds with mid-low micromolar inhibition, including the inhibitor **AVI-1084** [Z3535317212, median inhibitory concentration (IC_50_): 29 μM]. Informed by the docking poses, we sought to improve its potency by exploring the much larger 48 billion molecule space represented by the tangible library, an approach we have used previously (*11*–*14*). The docked pose of **AVI-1084** suggested favorable hydrogen bond interactions between its dihydrouracil core and the backbone of Glu^166^ and of Gly^143^ (fig. S1). With a simple structure, this scaffold was amenable to initial structure-activity relationship (SAR) expansion using the SmallWorld search engine (*15*) in ZINC22 (*16*), whereby we identified 17,123 purchasable analogs of **AVI-1084**. Each of these was docked into the M^Pro^ structure to evaluate complementarity with the binding site. From this effort, a total of 29 compounds (Fig. 1A, fig. S1, and table S1) were prioritized for synthesis and tested for activity in an M^Pro^ activity assay. Seven of the analogs showed improved activity, with the most potent, **AVI-3570**, exhibiting an IC_50_ of 1.5 μM (Fig. 1B). The improved potency of these analogs stemmed from the introduction of chloro or fluoro substituents augmenting nonpolar interactions with the M^Pro^ S2 pocket. We note that the 29 analogs predominantly focused on modifying the thiophene ring of **AVI-1084**, targeting the M^Pro^ S2 pocket, without addressing optimization of the inhibitor’s crucial pyridinone moiety that was modeled to bind the M^Pro^ active site in its S1 pocket. Cognizant that isoquinoline is a privileged structure for the S1 subsite in M^Pro^, we replaced the pyridinone ring with isoquinoline, leading to **AVI-3778**, **AVI-3779** (Fig. 1E), and **AVI-3780**; these analogs had submicromolar potencies, about 50-fold improved over the initial docking hit, **AVI-1084**.

To inform further optimization of this scaffold, the x-ray crystal structure of the low-micromolar inhibitor **AVI-3318** in complex with M^Pro^ was determined to 1.96 Å resolution (Fig. 1, C and D, and table S12). The docking pose of the inhibitor closely superposed with its experimental structure, with a root mean square deviation of 0.45 Å. With only minor discrepancies between the orientation of the chlorine substituent on the thiophene ring, all major interactions predicted by docking were confirmed in the crystal structure—the pyridinone side chain bound within the S1 pocket and in hydrogen-bonding contact with His^163^, the hydrophobic chlorothiophene group occupying the S2 pocket.

### Optimization of uracil scaffold and discovery of a latent electrophilic moiety affords low-nanomolar inhibitors

Fortified by the correspondence between the docking poses and the structure of the **AVI-3318**-M^Pro^ complex, we sought to expand the SAR beyond commercially available compounds, which were limited to substitutions of the dihydrouracil nitrogen atoms. By introducing side chains at the remaining two positions of the dihydrouracil core, we envisaged engaging the S1′ pocket to further improve potency. We also moved to an unsaturated uracil core, as this enabled the synthesis of putatively S1′-targeting analogs without introducing a stereocenter. Docking studies supported the potential of the proposed uracil-derived analogs to target multiple subsites in the active site.

**Fig. 2.**
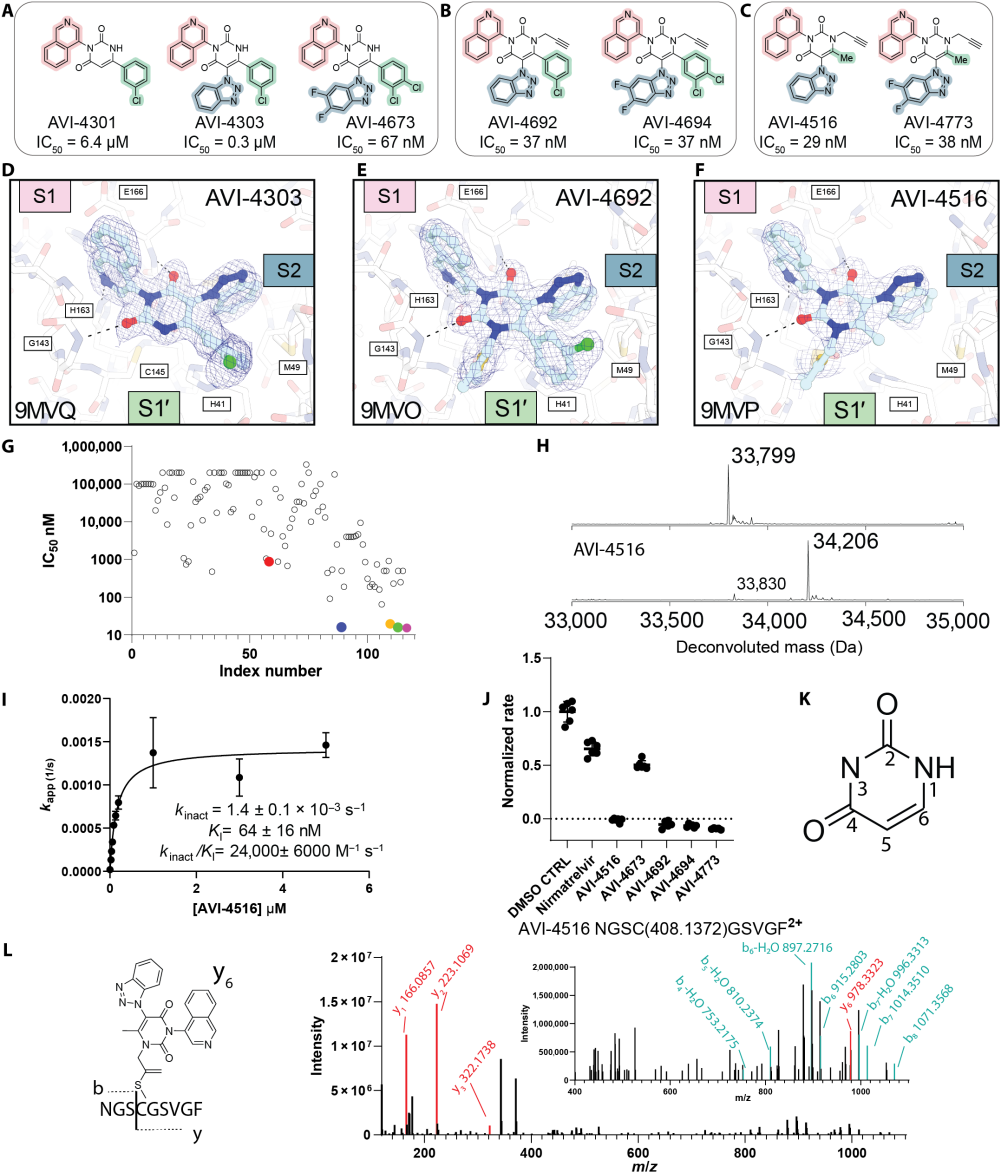
Medicinal chemistry optimization of the uracil scaffold and identification of latent electrophilic warhead. The arms of the compounds are highlighted according to the corresponding M^Pro^ subsite. Pink, S1; green, S1′; blue, S2. (**A**) Structures of notable compounds during optimization of noncovalent series with uracil core; **AVI-4673** is the most potent noncovalent. (**B**) Structure of the most potent C6-aryl covalent compounds. (**C**) The structures of the C6-methyl version of the covalent alkyne compounds, **AVI-4516** and **AVI-4773**. (**D** to **F**) Structures of M^Pro^ bound to inhibitors; the ligands were modeled using *F*_o_ − *F*_c_ electron density maps, with subsites annotated; **AVI-4303** and M^Pro^ costructure solved at a maximum resolution of 1.58 Å PDB: 9MVQ (D) **AVI-4692** and M^Pro^ costructure solved at a maximum resolution of 1.85 Å PDB: 9MVO (E) AVI-4516 and M^Pro^ costructure solved at a maximum resolution of 2.35 Å PDB: 9MVP (F). (**G**) Overall comparison of 116 compounds with measured biochemical IC_50_ values. **AVI-4303** is red, **AVI-4516** is blue, **AVI-4692** is yellow, **AVI-4694** is green, and **AVI-4773** is purple. (**H**) Deconvoluted whole protein denaturing mass spectrum of M^Pro^ alone, and M^Pro^ treated with **AVI-4516** indicating one modification. (**I**) Concentration of **AVI-4516** plotted against apparent inaction of M^Pro^ (*k*_app_) with calculated inhibitor kinetic parameters denoted. (**J**) Dialysis experiment demonstrating that **AVI-4516**, **AVI-4773**, **AVI-4692**, and **AVI-4694** are irreversible covalent inhibitors. (**K**) Uracil ring atom numbering nomenclature. (**L**) MS2 spectra of chymotrypic peptide of **AVI-4516** and the structure of y_6_ ion observed with proposed adduct bound to Cys^145^.

To access the desired uracil analogs, we used a convergent synthesis involving a cyclization reaction between an enaminone and isoquinoline carbamic ester. The enaminones were either commercially available or synthesized via the Blaise reaction (*17*). This synthesis afforded novel analogs with N3 and C6 substitution, but wherein the N1 position was necessarily unsubstituted. To introduce a C5 substituent, we applied a previously described amination procedure in the synthesis of the enaminone intermediate (*18*). Promising early analogs from this effort included **AVI-4301**, which was roughly equipotent to **AVI-3318**, and the C5 benzotriazole analog **AVI-4303** (Fig. 2, A and G, and fig. S4) that was encouragingly ~10-fold more potent, with an IC_50_ = 300 nM (Fig. 2A). A structure of **AVI-4303** bound to M^Pro^ at 1.58 Å (Fig. 2D and table S12) revealed that the isoquinoline occupied S1 as expected, whereas the benzotriazole unexpectedly bound the S2 pocket and the C6 chlorophenyl side chain stacked in a region between S1′ and S2 positioned near the catalytic dyad residue His^41^. Compared to the binding of **AVI-3318** then, the uracil core in **AVI-4303** was flipped ~180°, albeit still anchored by the strong preference for isoquinoline at S1. Both orientations of the uracil in **AVI-4303** were observed by docking, with the S2-bound pose very similar to the experimentally determined structure (fig. S3, G and H). Further gains in affinity were realized with the introduction of fluoro and choloro substituents on the benzotriazole and aryl rings, respectively, leading to **AVI-4673** with an IC_50_ value of 67 nM (Fig. 2A and fig. S4).

With the **AVI-4303** series binding primarily the S1 and S2 pockets (Fig. 2, A and D), we noted that the unsubstituted N1 position offered a potential vector toward the shallow and comparatively solvent-exposed S1′ pocket, which is also adjacent the catalytic Cys^145^. To explore binding preferences at S1′, we prepared the N1 propargyl analog **AVI-4516**, which we expected would provide access to diverse S1′ targeting analogs via Cu(I)-catalyzed azide-alkyne cycloaddition (CuAAC) reactions of the propargyl function. Unexpectedly, while various CuAAC adducts of **AVI-4516** exhibited only low-micromolar potencies, **AVI-4516** itself was very potent, with an IC_50_ of 29 nM (Fig. 2C and fig. S4), representing a 100-fold improvement over the des-propargyl comparator **AVI-4375** (IC_50_ 7.4 μM) (fig. S2). A close analog of **AVI-4516** bearing difluoro substitution of the benzotriazole ring (**AVI-4773**) was similarly potent at 38 nM (Fig. 2C and fig. S4). We then explored the effect of an N1 propargyl side chain in the context of C6-aryl analogs, finding that both **AVI-4692** and **AVI-4694** had potent M^Pro^ inhibition with identical IC_50_ values of 37 nM (Fig. 2B and fig. S4).

To confirm that inhibition was due to drug-like binding at the active site, **AVI-4516**, **AVI-4673**, **AVI-4773**, **AVI-4692**, and **AVI-4694** were tested for (artifactual) aggregation-induced inhibition. None of the compounds inhibited aggregation-prone inhibition of enzymes like β-lactamase and malate dehydrogenase up to a concentration of 10 μM (fig. S3, A and C). Of the five compounds, only **AVI-4694** formed particles by dynamic light scattering (DLS) (fig. S3B), but the critical aggregation concentration (CAC) was 4.3 μM (fig. S3D), a concentration 100-fold higher than required for M^Pro^ inhibition. Thus, dual lines of evidence suggested that C6-methyl (**AVI-4516/4773**) and C6-aryl (**AVI-4673/4694/4692**) analogs act by a drug-like mechanism and not by aggregation at inhibitory concentrations.

### N1 propargyl side chain is a latent electrophile

Given the expected proximity of Cys^145^ to the propargyl group in **AVI-4516**, we hypothesized that nucleophilic attack on the alkyne function might explain the compound’s markedly (~100-fold) improved potency compared to its des-propargyl comparator. Although rare and generally underappreciated, the reactivity of catalytic cysteines with unactivated alkynes has, in fact, been demonstrated for deubiquitinases (*9*), cathepsin K (*19*), and even M^Pro^ (*20*). Accordingly, we sought to confirm covalent engagement using several orthogonal methods. First, we evaluated several analogs of **AVI-4516** in which the propargyl side chain was replaced by allyl (**AVI-4690**), homo-propargyl (**AVI-5764**), or butynyl (**AVI-4774**) side chains. All three analogs were devoid of potent M^Pro^ activity with measured IC_50_ > 0.5 µM, a finding consistent with the hypothesis of covalent modification requiring precise positioning of a terminally unsubstituted alkyne (fig. S5). The nitrile congener **AVI-4689** was more active, but unusually ~5-fold less potent than **AVI-4516**. Next, we attempted to detect an **AVI-4516**–M^Pro^ adduct (Fig. 2H) by denatured, intact-protein mass-spectrometry (MS) and were pleased to observe a single modification consistent with the mass of **AVI-4516**. Next, we confirmed modification at cysteine (Cys^145^) by a chymotryptic-digested, fragment MS analysis (Fig. 2L). Whole protein MS experiments were also performed with the other propargyl analogs **AVI-4773**, **AVI-4692**, and **AVI-4694**; all showed mass shifts consistent with modification at a single site (fig. S6A). Modification of Cys^145^ by **AVI-4694** was confirmed by analysis of chymotryptic peptides similar to our procedure for **AVI-4516** (fig. S6B). Consistent with the latent (weakly) electrophilic nature of the propargyl group and the hypothesis of proximity-based reactivity, no covalent adduct was observed in incubations of **AVI-4516** with excess deuterated β-mercaptoethanol, as observed by ^1^H-NMR (fig. S8).

Next, kinetic parameters of inhibition by **AVI-4516** were then measured to test whether these compounds were consistent with covalent modification. **AVI-4516** exhibited *k*_inact_ and *K*_I_ values of 1.4 ± 0.1 × 10^−3^ s^−1^ and 64 ± 16 nM, respectively (Fig. 2I and fig. S7A). The modest *k*_inact_ is consistent with a weak electrophile and values reported for other alkyne warheads (*19*). Overall, the inactivation efficiency was reasonable for **AVI-4516** (*k*_inact_/*K*_I_ = 22,000 ± 6000 M^−1^ s^−1^), **AVI-4773** (*k*_inact_/*K*_I_ = 24,400 ± 700 M^−1^ s^−1^), **AVI-4692** (*k*_inact_/*K*_I_ = 27,000 ± 2200 M^−1^ s^−1^), and **AVI-4694** (*k*_inact_/*K*_I_ = 48,000 ± 6000 M^−1^ s^−1^) (Fig. 2I and fig. S7, F to H), and comparable to *k*_inact_/*K*_I_ values reported for other covalent protease inhibitors (*21*–*24*). Only **AVI-4516** shows saturation behavior in the assay and the *k*_inact_/*K*_I_ values for the latter three inhibitors were derived from a linear fit from the *k*_app_ versus inhibitor concentration plot; the raw traces (fig. S7, A and C to E) match what is expected for covalent inhibitors (loss of all activity over time) and the trend in inactivation efficiency matches the potency observed in cells (vide infra). When analyzing only the linear range of curve to best compare to the other inhibitors, **AVI-4516** exhibits a *k*_inact_ /*K*_I_ = 8500 ± 690 M^−1^ s^−1^ (fig. S3B), which is consistent with the three other inhibitors exhibiting greater potency than **AVI-4516**. To determine whether the presumed thioenol ether adduct was subject to hydrolytic instability and regeneration of functional enzyme, a dialysis experiment was performed wherein M^Pro^ was treated with a slight excess of compound (1.5 equiv) and incubated for 4 hours, after which dialysis was performed for 20 hours (Fig. 2J) or for 7 days (fig. S7I). In these experiments, M^Pro^ activity was not regained after inhibition by **AVI-4516**, **AVI-4773**, **AVI-4692**, or **AVI-4694**, confirming that the modification is irreversible and the adduct is stable over the timescale examined. In contrast, the thioimidate adduct formed upon incubation with nirmatrelvir was not stable, and partial activity was restored on dialysis, consistent with the expected reversible-covalent reactivity of nitrile warheads (Fig. 2K and fig. S7H). These results also suggest that our IC_50_ values are overestimated and are often close to approximately half the enzyme used and thus the *k*_inact_/*K*_I_ values allow us to rank these inhibitors for biochemical SAR.

Last, we were able to obtain crystal structures of N1 propargyl analogs **AVI-4516** and **AVI-4692** bound to M^Pro^ (Fig. 2, E and F). Although **AVI-4516** was solved at 2.35 Å resolution, **AVI-4692** was solved at 1.85 Å, allowing for a detailed analysis of the binding mode (table S12). Both compounds retained the same global binding mode of des-propargyl progenitor **AVI-4303**, with their isoquinoline moieties bound in S1 and benzotriazole substituents in S2. The density surrounding Cys^145^ is most consistent with a thioenol ether adduct formed by reaction at the internal carbon of the alkyne function (fig. S16). This mode of reactivity is also that suggested by structures of M^Pro^ bound to an alkyne analog of nirmatrelvir (PDB: 8B2T) and reports of other propargyl-based cysteine warheads (*9*, *19*). Both the **AVI-4516** and **AVI-4692** structures exhibited partial occupancy and extra density near Cys^145^ that is suggestive of partial oxidation of the sulfur, a plausible result of the extended soaks performed to generate these structures.

### M^Pro^ inhibitors exhibit efficacy in SARS-CoV-2–infected cells

**Fig. 3.**
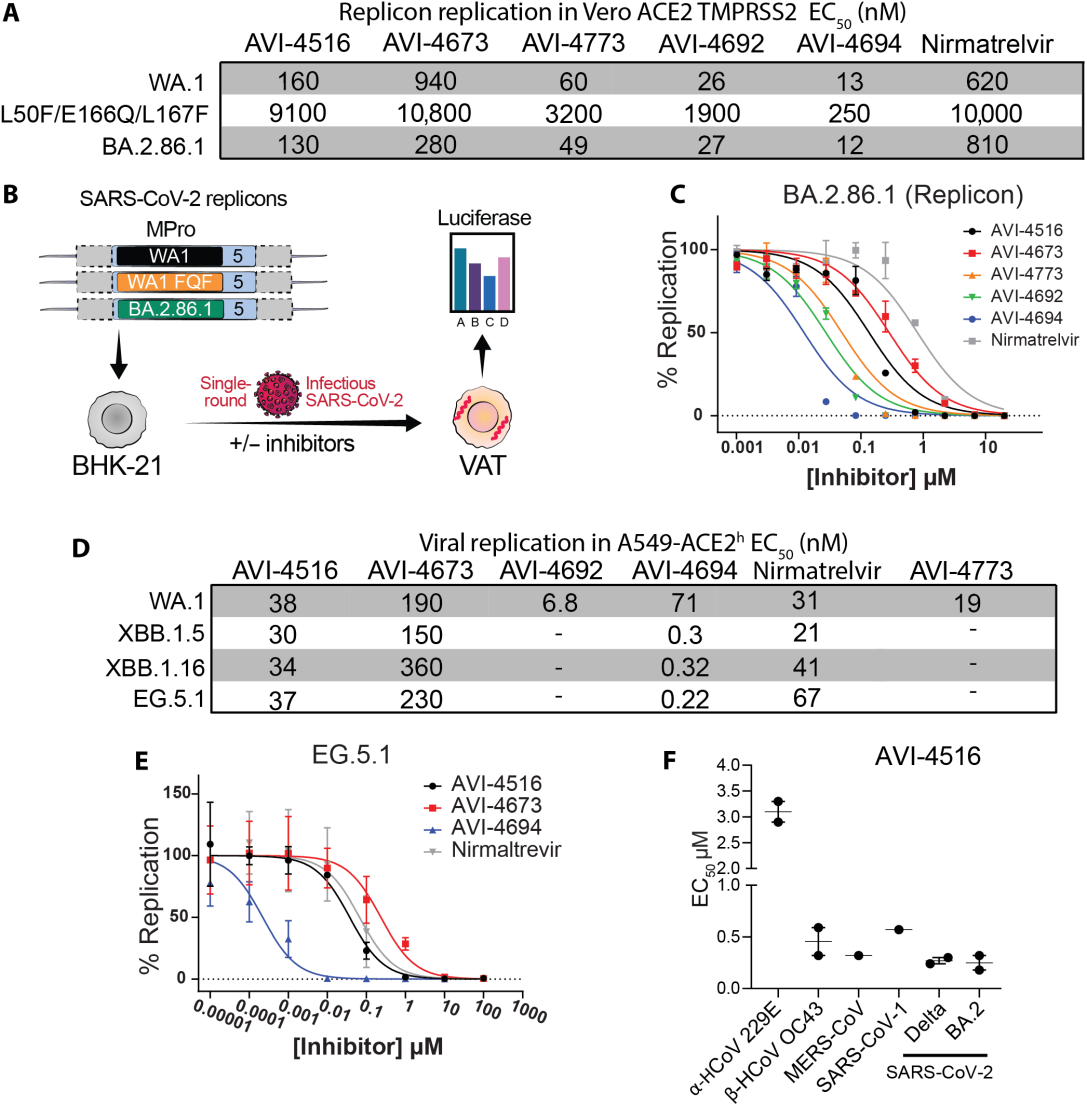
In cellulo efficacy of M^Pro^ inhibitors against SARS-CoV-2 strains, related coronaviruses, and nirmatrelvir-resistant mutations. (**A**) A table of EC_50_ values for dose-response inhibition of viral replication in replicon-based assay. The BA.2.86.1 curve is presented below (C) and the rest are presented in fig. S9 (A and B). (**B**) A schematic cartoon of the replicon assay for measurement of antiviral potency. (**C**) Dose-response curves for selected compounds in the BA.2.86.1 replicon assay. (**D**) A table of EC_50_ values for dose-response inhibition of viral replication in the live virus–based assay. The EG.5.1 curve is presented below (E) and the other strains are presented in fig. S9 (C to E). (**E**) Dose-response curves for selected compounds against EG.5.1 live virus SARS-CoV-2 variant in A549-ACE2^h^ cells. (**F**) **AVI-4516** EC_50_ values for pan-coronavirus antiviral efficacy screen determined through CPE. All error bars are plotted as ±SD.

Before antiviral efficacy assessment in cells, all compounds were evaluated for permeability in a parallel artificial membrane permeability assay (PAMPA) and for cytotoxicity in A549-ACE2^h^ cells (table S2). After demonstrating high passive permeability and a lack of acute cellular toxicity, the most promising compounds were evaluated for antiviral efficacy using a previously described SARS-CoV-2 replicon assay (*25*, *26*). In this assay, the SARS-CoV-2 Spike coding sequence is replaced with luciferase and fluorescence reporters to conduct single-round infection and rapid testing of many compounds (Fig. 3B). The reporter activity from replicon-infected cells has been validated as a surrogate of viral RNA replication (*25*). Several of the lead molecules inhibited viral RNA replication in cells (Fig. 3A and fig. S9A); the C6-aryl analogs, **AVI-4692** and **AVI-4694**, exhibited excellent median effective concentration (EC_50_) values of 26 and 13 nM, respectively.

To assess the lead compounds’ activity in the context of nirmatrelvir-resistant M^Pro^ [E166Q, A173V, and S144A (*27*)], we evaluated their activity on purified M^Pro^ containing these specific mutations. **AVI-4516** maintained low-nanomolar potency only against the E166Q enzyme with an IC_50_ of 25 nM (fig. S10, A to D) while the other tested compounds lost considerable activity (≥10-fold) when compared to their wild-type (WT) M^Pro^ IC_50_ values (Fig. 3, A, B, and E). To determine whether this trend held in the replicon system, compounds were then tested against a triple mutant that contains in addition to E166Q two more mutations in M^Pro^ (L50F, L167F). These are the most prevalent resistant mutations at each respective position in natural sequences on the Global Initiative on Sharing All Influenza Data database (Fig. 3A and fig. S9B). Although **AVI-4516** and **AVI-4773** maintained potency similar to nirmatrelvir in this triple mutant, **AVI-4692** and **AVI-4694** exhibited enhanced potency, likely due to a combination of better permeability and improved inhibitory properties. Notably, all tested compounds exhibited slightly better efficacy against a recent Omicron strain (BA.2.86.1) that was an ancestor of the currently circulating KP.3 variant and contains the relatively fixed P132H mutation (*28*, *29*) (Fig. 3, A and C), suggesting that P132H likely does not affect compound binding.

To determine the inhibitory activity of compounds in authentic replicating SARS-CoV-2, we used an antiviral Incucyte-based high-throughput screen using multiple SARS-CoV-2 variants. In this assay, we used a genetically encoded fluorescent reporter virus, where the Orf7a and Orf7b coding sequences were replaced with the reporter mNeonGreen (mNG), icSARS-CoV-2-mNG (*30*). This reporter virus was then used to generate recent Omicron variant viruses. The infectivity and replication of the WA1, XBB.1.5, XBB.1.16, and EG.5.1 were evaluated to optimize signal-to-noise ratio. As expected, we found that nirmatrelvir was potent against all viruses tested with an EC_50_ ranging from 21 to 67 nM consistent with previously reported values (*31*) and has better potency than in the replicon-infected Vero ACE2 TMPRSS2 cells, which have high expression of the xenobiotic transporter, P-glycoprotein (P-gp) (*32*). The noncovalent analog **AVI-4673**, as well as covalent C6-methyl, **AVI-4516**, and C6-aryl **AVI-4694** showed potent antiviral efficacy against the ancestral SARS-CoV-2 WA.1 variant (**AVI-4516** EC_50_ = 38 nM, **AVI-4673** EC_50_ = 190 nM, **AVI-4694** EC_50_ = 71 nM) while **AVI-4694** was remarkably ≥100-fold more potent than nirmatrelvir against the recent Omicron variants XBB.1.5, XBB.1.16, and EG.5.1, with EC_50_ values of 0.30, 0.32, and 0.22 nM, respectively (Fig. 3, B to D, and fig. S9, D and E). Together, the biochemical, replicon, and live virus assay data suggest that the combination of C6-aryl substitution with the propargyl warhead (as in **AVI-4694**) has great potential to produce an agent that effectively targets recently emergent variants of SARS-CoV-2.

Promisingly, the lead molecules exhibit high potency against SARS-CoV-1 M^Pro^ (fig. S10) in biochemical assays. To test whether this was a general result and whether the lead molecules exhibited pan-coronavirus activity, two covalent analogs (one C6-methyl and one C6-aryl) were further tested against live viruses. The viruses tested included human common cold coronaviruses (α-HCoV 229E and β-HCoV OC43), MERS-CoV, SARS-CoV, and various SARS-CoV-2 variants (Delta, BA.2). **AVI-4516** and **AVI-4694** demonstrated pan-coronavirus activity with low EC_50_ values (<3 μM) and high selectivity indices [50% selectivity index (SI_50_) > 170] against all tested variants (Fig. 3F and table S11). These findings suggest that **AVI-4516** and **AVI-4694** could serve as effective pan-coronavirus inhibitors. The pan-coronavirus activity is consistent with reactivity directed by the presence of an oxyanion hole positioned near a reactive cysteine and conserved S1 and S2 pockets. We aligned the sequences of coronavirus M^Pro^ enzymes from multiple families to evaluate sequence conservation and generate a sequence similarity network of all searchable coronaviruses (fig. S19, A and B). Further mining these data, we generated sequence logos (fig. S20) to visualize the conservation of residues that either directly contact **AVI-4516** or **AVI-4692** or are involved in second-sphere contact with direct-interacting residues. This analysis revealed residues that are highly conserved for all families and are thus high-priority residues for targeting by future analogs. Of note is that C6-aryl analog **AVI-4692** engages the S1′ subsite and forms an edge-face interaction with strictly conserved His^41^, the catalytic base in coronavirus M^Pro^ enzymes.

There have been some reports of M^Pro^ inhibitors that exhibit synergy (*33*) when used in combination with RNA-dependent RNA polymerase (RdRp) inhibitors. We tested our scaffold to determine whether these inhibitors could synergize with an RdRp inhibitor. The inhibitor, molnupiravir, was chosen as it is clinically approved and available orally, which could facilitate further studies if successful. Cells that were infected with either WA.1 or XBB.1.16 were then tested with **AVI-4516** and molnupiravir to measure synergy between a C6-methyl compound and the RdRp inhibitor (figs. S17, A to F, and S18). For the WA.1 strain–infected cells, a minor effect was observed in the direction of positive synergy. However, for the XBB.1.16 strain of SARS-CoV-2 (fig. S17, D to F), synergy was observed at several concentrations when using a Zero Interaction Potency (ZIP) synergy analysis (*34*).

### AVI-4516 has an excellent pharmacokinetic and in vitro off-target safety profile

**Fig. 4.**
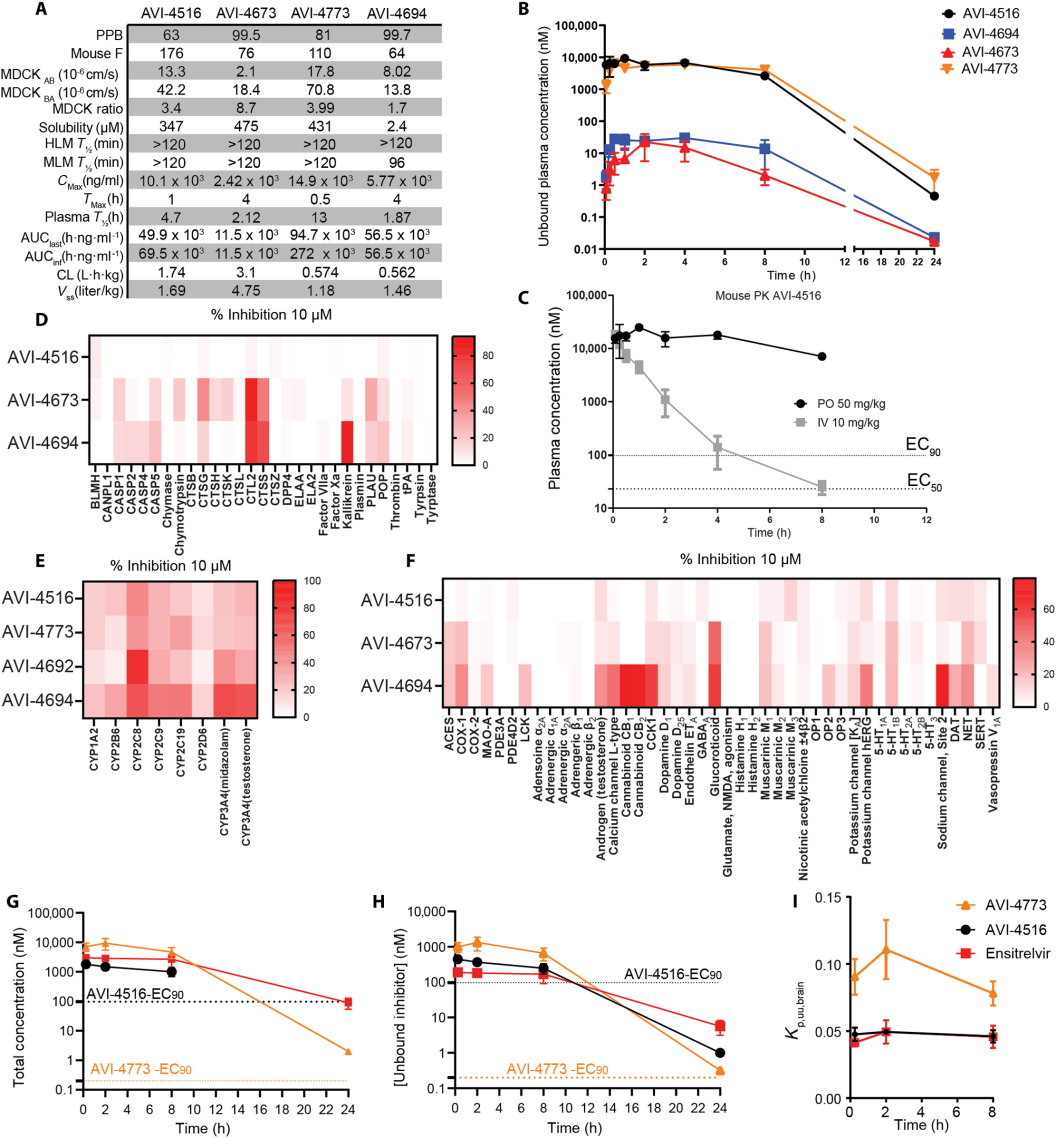
ADME, PK, and safety properties of M^Pro^ inhibitors. (**A**) ADME and PK properties of **AVI-4516**, **AVI-4773**, **AVI-4692**, and **AVI-4694**. (**B**) Unbound concentration in mouse plasma of **AVI-4516**, **AVI-4773**, **AVI-4692**, and **AVI-4694** after 50 mg/kg po dosing. (**C**) Comparison of mouse plasma concentration of **AVI-4516** when dosed 10 mg/kg iv or 50 mg/kg po. The concentration at 24 hours was below the LOQ. (**D**) Percent inhibition of mammalian peptidase panel when treated with 10 μM **AVI-4516**, **AVI-4673**, and **AVI-4694**. **AVI-4516** has low inhibition across the panel. Proteases that inhibited >50% have dose-response curves that generated IC_50_ values in fig. S13. (**E**) Percent inhibition of human CYP panel of **AVI-4516**, **AVI-4773**, **AVI-4692**, and **AVI-4694** compared to nirmatrelvir and ensitrelvir. (**F**) Percent inhibition of mammalian receptor panel when treated with 10 μM **AVI-4516**, **AVI-4673**, and **AVI-4694**. **AVI-4516** has low inhibition across the panel. Proteins that were inhibited >50% have dose-response curves that generated IC_50_ values in fig. S13 (A and B). (**G**) Total brain and plasma concentration of **AVI-4516**, **AVI-4773**, and ensitrelvir after the 100 mg/kg po dose. All error bars are plotted as ± SD. (**H**) Unbound concentration of **AVI-4516**, **AVI-4773**, and ensitrelvir in mouse brain. (**I**) Unbound brain to plasma partitioning coefficient for **AVI-4516**, **AVI-4773**, and ensitrelvir.

To guide our lead optimization efforts, we performed a panel of standard in vitro absorption, distribution, metabolism, and excretion (ADME) assays for all new analogs exhibiting potent biochemical activity. Among the leads described herein, **AVI-4516**, **AVI-4773**, **AVI-4692**, and **AVI-4694** all exhibited excellent stability in mouse and human liver microsomes (MLM and HLM *T*_1/2_ > 120 min, Fig. 4A). The C6 methyl analogs **AVI-4516** and **AVI-4773** showed plasma protein binding (PPB) that was low to moderate at 61 and 83%, respectively, while C6 aryl analogs **AVI-4692** and **AVI-4694**, by contrast, had very high PPB at >99% (Fig. 4A). Permeability in MDCK-MDR1 monolayers that express P-gp were high in the apical-to-basolateral direction for **AVI-4516** (13.3 × 10^−6^ cm/s), **AVI-4773** (17.8 × 10^−6^ cm/s) and **AVI-4694** (8.2 × 10^−6^ cm/s) while efflux ratios were reasonable to low at 3.4, 3.99, and 1.7, respectively (Fig. 4A). The combined in vitro antiviral and ADME data suggested excellent potential for **AVI-4516**, **AVI-4773**, and **AVI-4694** to achieve efficacious plasma and cell/tissue concentrations in animals and thus were nominated for in vivo pharmacokinetic (PK) profiling.

We chose doses of 50 mg/kg po (oral) and 10 mg/kg iv (intravenous) for PK experiments with **AVI-4516**, **AVI-4773**, **AVI-4694,** and **AVI-4673**, which were performed in male CD-1 mice (Fig. 4B; fig. S11, A to D; and tables S4 to S7). All four compounds showed low clearance, and remarkably so for **AVI-4694** and **AVI-4773** with in vivo clearance just ~7% of hepatic blood flow in the mouse. Total exposure by area under the curve (AUC) was the highest for C6 methyl analogs **AVI-4773** and **AVI-4516** and the lowest for **AVI-4673**, consistent with the considerably lower permeability of this analog in the MDCK-MDR1 assay. Overall, the plasma exposure profiles and oral bioavailability of the lead compounds were excellent, with the oral bioavailability (%*F*) values of **AVI-4516** and **AVI-4773** exceeding 100%. These very high apparent *F* values may reflect a slow intestinal absorption process (*35*) or might be due to saturation of clearance mechanisms at the rather high oral dose of 50 mg/kg (as compared to 10 mg/kg in the intravenous arm). Using measured PPB values returned a free concentration of 2615 nM for **AVI-4516** and 4045 nM for **AVI-4773** at the 8-hour time point (Fig. 4B), values approximately 26-fold higher than the cellular 90% effective concentration (EC_90_) = 97 nM for **AVI-4516** in the WA.1 strain (Fig. 3A). Accordingly, we predicted that a 50 mg/kg or higher dose of either **AVI-4516** or **AVI-4773** should retain efficacious antiviral concentrations at 12 hours and that twice-daily (BID) dosing would be effective in mouse efficacy studies.

Paxlovid, ensitrelvir, and other recently reported (*4*, *5*, *36*–*38*) preclinical agents are known to be inhibitors of CYP3A4, with the potential for drug-drug interactions that must be monitored and can lead to adverse events in some patient populations. Accordingly, we evaluated **AVI-4516**, **AVI-4773**, **AVI-4692**, and **AVI-4694**, for inhibition of important human cytochrome P450 (CYP) isoforms at a fixed concentration of 10 μM. Both **AVI-4516** and **AVI-4773** exhibited minimal inhibition (≤25% at 10 μM) across the panel, with the exception of CYP2C8 (~45% at 10 μM). The C6-aryl analog **AVI-4692**, by contrast, was a somewhat more potent inhibitor of both CYP2C8 and CYP3A4 while **AVI-4694** showed the overall poorest profile, inhibiting several CYP isoforms >50% at 10 μM (Fig. 4E). We next evaluated three exemplar analogs: **AVI-4673** (noncovalent analog), **AVI-4516** (C6 methyl analog), and **AVI-4694** (C6 aryl analog) for off-target activity across a panel of 40 receptors, ion channels (including the hERG channel), and serine and cysteine proteases (Fig. 4, D and F). Of the three leads, latent-electrophilic analog **AVI-4516** bearing C6 methyl substitution showed the most exceptional in vitro safety profile, demonstrating no substantial inhibition or interference with any of the off-targets at 10 μM. Of the other leads, noncovalent analog **AVI-4673** was found to be a low-micromolar inhibitor of cathepsin L2 (fig. S13A), while C6 aryl analog **AVI-4694** showed low-micromolar inhibition of nine enzymes/receptors in the panel (fig. S13B). To evaluate the selectivity of **AVI-4516** across the cellular proteome, we turned to thermal proteome profiling (TPP) (*39*). To establish a baseline, we measured the melting temperature (*T*_m_) shift of recombinant M^Pro^ treated with **AVI-4516** and observed a shift in *T*_m_ of ≥15°C (fig. S12A). We next treated cellular lysate from A549 cells with **AVI-4516** for TPP analysis, which uses a proteomic analysis to detect protein stabilization across complex proteomes. Notably, we found only one protein, HAPLN1, that showed a statistically significant increase in *T*_m_ of 5.77°C. In summary, these studies revealed a remarkably clean off-target binding profile for the C6-methyl-N-propargyl uracil chemotype exemplified by **AVI-4516** (fig. S12, B and C).

Given the superior off-target selectivity profile of the N-propargyl/C6-methyl chemotype, we sought to explore the distribution of **AVI-4516** across mouse tissues following a 100 mg/kg oral dose. Of particular interest was exposure in lung, bronchial alveolar fluid (BALF), and brain, given that infection is centered in the respiratory tract, while reservoirs of virus may persist in brain (*40*). Encouragingly, we found that **AVI-4516** and **AVI-4773** were more significantly distributed into pharmacologically relevant compartments like lung and BALF than was ensitrelvir. Thus, **AVI-4516** maintains very high exposure compared to its cellular EC_90_ (WA.1 live virus Fig. 3B and fig. S9C) at 8 hours in mouse heart (227× EC_90_), lungs (47× EC_90_), and BALF (11.8× EC_90_) (fig. S11, E and F, and table S8) while **AVI-4773** exhibited even higher exposures in these tissues, which was especially notable given its even lower cellular EC_90_ values (fig. S11, G and H, and table S10). For both analogs, exposure in brain was considerably lower than in plasma or other tissues, but total brain concentration of **AVI-4516** at 8 hours (Fig. 4G) was still ~10-fold greater than its antiviral EC_90_ versus the WA.1 strain_._ On account of its ~4.7-fold higher exposure in brain and nanomolar EC_90_ value, the total brain concentration of **AVI-4773** at 8 hours was ~1000-fold greater than its cellular EC_90_ versus the WA.1 strain. Using measured binding to mouse plasma protein and brain homogenate, we calculate an unbound brain-to-plasma partitioning coefficient (*K*_P,uu,brain_) of ~5 and ~8% for **AVI-4516** and **AVI-4773**, respectively, at 8 hours. Overall, the biodistribution studies demonstrate that **AVI-4773** is more favorably partitioned into the lung, heart, and BALF when compared to ensitrelvir, while exhibiting ~10-fold higher free drug concentrations in brain 8 hours after a single oral dose (Fig. 4H). While directly analogous data for nirmatrelvir are unavailable, a study of nirmatrelvir in the rat (when dosed at an allometrically scaled dose of 60 mg/kg nirmatrelvir and 20 mg/kg ritonavir/day) revealed brain concentrations only three times the respective EC_90_ value (*41*). In summary, the favorable PK profile, significant free fraction, and favorable biodistribution profile of **AVI-4516** and **AVI-4773** nominated these compounds as promising lead compounds for in vivo studies of antiviral efficacy.

### AVI-4516 and AVI-4773 demonstrate potent in vivo antiviral efficacy

**Fig. 5.**
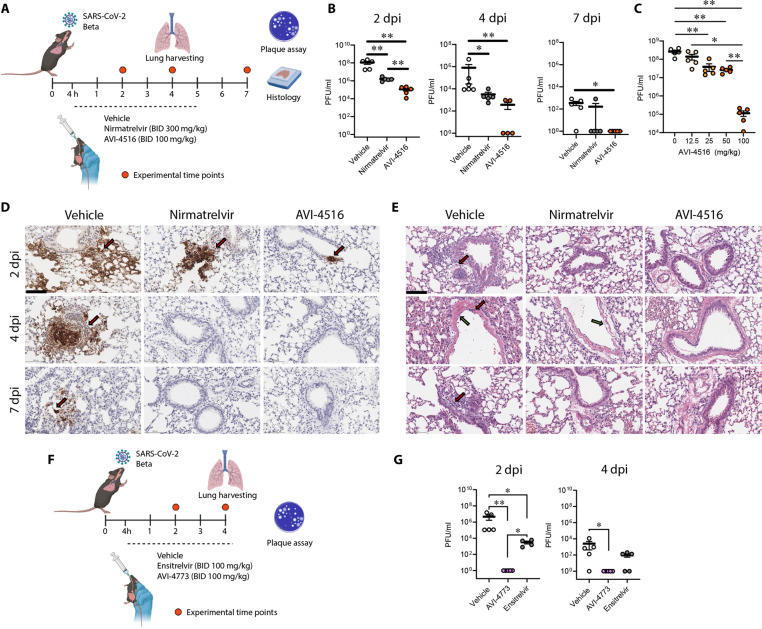
Oral administration of AVI-4516 limits virus replication. (**A**) Schematic of antiviral efficacy experiment. WT mice were intranasally infected with 10^3^ PFU of the SARS-CoV-2 Beta variant. Infected animals were orally dosed (BID) with either vehicle, nirmatrelvir, or **AVI-4516**. The lung tissues were harvested and processed for further analysis at 2, 4, and 7 days postinfection (dpi) (*n* = 5 per group per time point). (**B**) Graphs presenting mature virus particles from the lungs of infected mice at the indicated time points. Each dot represents the infectious virus titer in an individual mouse. (**C**) A separate group of mice were infected with the SARS-CoV-2 Beta variant and treated with various concentrations of **AVI-4516**. Virus titers were measured at 2 dpi. (**D**) Representative images of immunohistochemistry for the SARS-CoV-2 N protein in the left lung lobe of mice from different treatment groups at the specified time points. (**E**) Hematoxylin and eosin (H-E) staining of lung tissue. Significant mononuclear cell infiltrations were marked by red arrows, and severe injury in respiratory epithelia, characterized by epithelial cell debris in the lumen and incomplete epithelial regeneration, was highlighted by green arrows. (**F**) Schematic of antiviral efficacy experiment. WT mice were intranasally infected with 103 PFU of the SARS-CoV-2 Beta variant. Infected animals were orally dosed (BID) with either vehicle, ensitrelvir, or **AVI-4773**. The lung tissues were harvested and processed for further analysis at 2 and 4 dpi (*n* = 5 per group per time point). (**G**) Graphs presenting mature virus particles from the lungs of infected mice at the indicated time points are presented. Each dot represents the infectious virus titer in an individual mouse. All data points are presented as mean ± SEM for each time point and were analyzed using a two-tailed unpaired Student’s *t* test. Scale bars, 100 μm.

We next turned to a mouse infection model to elucidate the in vivo antiviral effects of irreversible M^pro^ inhibition by **AVI-4516** and **AVI-4773**. An initial study in C57BL/6 mice infected with the SARS-CoV-2 Beta compared nirmatrelvir (300 mg/kg BID) and **AVI-4516** (100 mg/kg BID) with vehicle-treated animals. Of note, the Beta variant contains a natural mutation (N501Y) in its Spike protein that allows nonlethal infection of WT mice, while M^Pro^ of the Beta variant contains the K90R mutation (*42*). As shown in the schematic (Fig. 5A), treatment began at 4 hours postinfection with oral BID dosing continuing for 5 days postinfection (dpi), during which we closely monitored body weight as a marker for severity of infection (fig. S15A). At 2, 4, and 7 dpi, a subset of mice (*n* = 5) from each group was euthanized to determine the virus titers through plaque assays (Fig. 5B).

As expected, for this model, we observed no weight loss in any treatment group until day 7 (fig. S14A). Encouragingly, measurement of mature virus particles using plaque assays revealed the potent antiviral efficacy of **AVI-4516**, with a 3- to 4-log reduction in virus replication compared to the vehicle, while the positive control, nirmatrelvir, showed a 2-log reduction (Fig. 5B). Histology data indicated heightened staining for the SARS-CoV-2 N protein in the vehicle treatment group through immunohistochemistry. In contrast, the nirmatrelvir and **AVI-4516** treatment groups exhibited minimal or no viral protein present at all time points (Fig. 5D and fig. S13A). Peribronchiolar and perivascular infiltrations by mononuclear cells and respiratory epithelial cell injury were observed in the vehicle-treated group, to a minimal extent in the nirmatrelvir-treated group, and were notably absent in the **AVI-4516**-treated mouse lungs, underscoring the rapid action and superior efficacy of **AVI-4516** compared to a higher dose of nirmatrelvir in this model (Fig. 5E and fig. S13B).

In an independent experiment, the dose-dependent antiviral efficacy of **AVI-4516** was determined using SARS-CoV-2 Beta–infected WT mice, where the mice were treated orally with **AVI-4516** at doses ranging from 12.5 to 100 mg/kg. The animals were euthanized at day 2 postinfection to determine the virus titers in the lung tissues. The results of this study demonstrated a dose-dependent decrease in virus replication and allowed the calculation of an IC_50_ value of 14.7 mg/kg (Fig. 5C). From these data, it is evident that three doses of **AVI-4516** at 25 mg/kg is sufficient to significantly reduce viral load at 2 dpi. In summary, **AVI-4516** exhibits significant dose-dependent antiviral efficacy, demonstrating a significant reduction in mature virus particle production compared to the vehicle- and nirmatrelvir-treated mice. In addition, the histology data show that early treatment with **AVI-4516** mitigates signs of lung inflammation.

Encouraged by the superior efficacy of **AVI-4516** as compared to nirmatrelvir, we next compared the efficacy of **AVI-4516** and difluoro congener **AVI-4773** to ensitrelvir, which is regarded as more efficacious than single-agent nirmatrelvir in mouse models (*43*) and in our models performed better at a third the dose. Thus, a series of experiments were performed comparing **AVI-4516** or **AVI-4773** to ensitrelvir using a 100 mg/kg BID dosing regimen. In the experiments with **AVI-4516** and ensitrelvir, we observed similar reductions in viral titers for the two test articles, as compared with vehicle-treated controls at 2 dpi (fig. S15B). More notably, we found that **AVI-4773** conferred a marked and rapid reduction of viral titers to below detectable levels by day 2, after just three doses (Fig. 5F). This represented a >3-log reduction in viral load at day 2 when compared to the ensitrelvir-treated arm and a ~6-log reduction compared to vehicle-treated animals at day 2 (Fig. 5G). In a second, identical study, we confirmed this powerful pharmacodynamic effect, with a reduction of viral titers to below detectable levels after just three doses of **AVI-4773** (fig. S15C). The remarkable pharmacodynamics of **AVI-4773** can be understood in light of the compound’s high exposure in BALF of ~4400 nM at 8 hours after a single oral dose (fig. S11H) and potent, low-nanomolar, or subnanomolar antiviral effects in cellular models.

## DISCUSSION

The approval of nirmatrelvir and ensitrelvir for clinical use was followed rapidly by efforts from various academic and industrial groups to identify improved, next-generation inhibitors of SARS-CoV-2 M^Pro^. Many of these next-generation compounds are inspired by, or based on nirmatrelvir, differing in the side chains and the nature of the cysteine-targeting warhead (*36*–*38*, *44*, *45*). A second class of inhibitors are entirely nonpeptidic in nature and based on cyclic uracil, dihydrouracil, or hydantoin cores (*5*, *33*, *46*–*49*) from which aromatic or aliphatic arms are displayed. These latter compounds, such as ensitrelvir, act by reversible, noncovalent mechanisms of inhibition. Here, we describe a distinct chemotype (fig. S3F) that combines the general trifold architecture of ensitrelvir with a latent-electrophilic warhead, resulting in a unique mechanism of M^Pro^ inhibition and differentiated antiviral and pharmacodynamic properties. These improvements are exemplified by the exquisite off-target safety profile and potent pan-coronaviral in cellulo activity of **AVI-4516** and the superior pharmacokinetics and tissue distribution of **AVI-4773**, which, together with potent antiviral activity, produce a remarkable pharmacodynamic effect in infected mice.

The rapid reduction of viral titers conferred by **AVI-4516,** and especially **AVI-4773**, suggests the potential for more convenient, less frequent dosing regimens and might extend the window for effective treatment of infection. Further preclinical assessment will be required to predict a human dose and to determine optimal dosing regiments of **AVI-4773** or further-optimized analogs from this scaffold. At present, the remarkably rapid killing effects of **AVI-4773** in animals stands out among other recently disclosed M^Pro^ inhibitors. In part, these properties derive from the unique mode of inhibition conferred by an unactivated N-propargyl side chain. We posit that successful capture of Cys^145^ with this very weak electrophile requires placement of the alkyne function near the oxyanion hole, which promotes the nucleophilic attack of Cys^145^ by stabilizing the developing negative charge at the terminal carbon and its eventual protonation, plausibly by His^41^ of the catalytic dyad. Thus, the irreversible inhibition of M^Pro^ by **AVI-4516**, **AVI-4773**, and related analogs distinguish this chemotype mechanistically from nirmatrelvir (reversible-covalent) and ensitrelvir (noncovalent), while offering apparent advantages in terms of target engagement and pharmacodynamic effect, which we continue to explore. It is worth noting here that the 4-aminoisoquinoline ring has been reported to be genotoxic by the AMES assay (*50*) and the possibility that this free species could be generated by metabolism of the uracil ring represents a potential development liability for current isoquinoline-based leads. Ongoing optimization of this series includes exploring replacement of isoquinoline with related heterocycles known to be AMES-negative (*50*). Additional features of this new chemotype include a simple structure and straightforward synthesis in four or five steps, which implies good potential for low manufacturing costs, an important criterion in the context of global coronavirus pandemic preparedness, and the stockpiling of drug substance.

In nirmatrelvir-resistant mutants, **AVI-4516** performs at least as well as nirmatrelvir while C6-aryl congeners (e.g., **AVI-4694**) exhibit even more potent antiviral activity, likely due to a combination of improved permeability and the formation of additional active-site contacts beyond those of nirmatrelvir. The C6-chlorophenyl substituent of **AVI-4692** contacts His^41^ of the catalytic dyad, which is absolutely conserved across all coronavirus M^Pro^ enzymes and may, in part, explain the enhanced spectrum of this chemotype. In addition, all the residues that make contact with both **AVI-4694** and **AVI-4516** are highly conserved across the Beta coronavirus family with many conserved across the other three families. The C6-aryl subcategory is thus a promising one for further expansion of antiviral spectrum to combat current and future variants. Synergy has been explored previously as a treatment modality for SARS-CoV-2 that has the potential to circumvent mutational pressure (*33*). Promisingly, **AVI-4516** has synergy with an orally available RdRp inhibitor in a cellular infection model with the Omicron strain of SARS-CoV-2, XBB.1.16.

Coronaviruses can infect the brain, leading to inflammatory syndrome and diverse neurological symptoms, and possibly even contributing to poorly understood conditions like “long COVID.” As demonstrated here, **AVI-4773** crosses the blood-brain barrier in mice, with an unbound brain concentration ~8% of that in plasma and at least 1000-fold higher than the antiviral EC_90_ at 8 hours. This suggests **AVI-4773** as a potentially valuable in vivo tool compound to better understand coronavirus infection and the brain. Above all, the discovery of compounds such as **AVI-4516** and **AVI-4773** reveals an advanced preclinical lead series with differentiated properties and excellent prospects to deliver a pan-coronavirus therapeutic development candidate. This discovery approach and unique mechanism of inhibition of these compounds also provide a roadmap for the discovery of antiviral scaffolds that target cysteine proteases of other viruses of concern.

## MATERIALS AND METHODS

### Noncovalent optimization

Analogs for docking hit Z3535317212 were queried in SmallWorld 48 billion make-on-demand libraries (*15*). The resulting analogs were further filtered based on Tc > 0.5 and docked to the M^Pro^-x11612 as described in the previous docking campaign (*10*). Compounds were also designed by modifying the two-dimensional structure and custom synthesis by Enamine Ltd. (Kyïv, Ukraine). The docked poses were visually inspected for compatibility with the site, and prioritized analogs were synthesized and tested. Make-on-demand noncovalent analogs were purchased and synthesized by Enamine Ltd. Purities of molecules were at least 90% and most active compounds were at least 95% (assessed by LC/MS data).

### M^Pro^ expression and purification

The M^Pro^ expression plasmid was generated as previously described (*10*) with slight modifications. BL21 pLyS Ros2 (DE3) cells were transformed with the expression plasmid. A single colony was used to start an overnight culture in LB media supplemented with ampicillin (100 μg/ml) and chloramphenicol (20 μg/ml). This overnight culture was diluted 1:50 to inoculate 2XYT media supplemented with ampicillin (100 μg/ml) and chloramphenicol (20 μg/ml). These cultures grew at 37°C until the OD_600_ (optimal density at 600 nm) reached approximately 1 to 2.0, at which point the temperature was reduced to 20°C and isopropyl-β-D-thiogalactopyranoside was added to a final concentration of 1 mM induced overnight. The cultures were then harvested by centrifugation at 4000*g* for 20 min at 4°C. The pellet was then resuspended in 50 mM tris, pH 8.0, 400 mM NaCl, and 20 mM imidazole with DNase I (0.1 mg/ml) and lysozyme (0.1 mg/ml; Sigma). The resuspended pellet was lysed by sonication and then clarified with centrifugation at 42,000*g* for 30 min. The clarified lysate was loaded onto pre-equilibrated Ni-NTA resin with buffer A (400 mM NaCl, 50 mM tris, and 20 mM imidazole, pH 8.0). The column was then washed with 20× column volume (CV) of buffer A and eluted in a step gradient of imidazole from 20 to 500 mM. The fractions were analyzed with SDS–polyacrylamide gel electrophoresis for purity. Pure fractions were pooled and concentration to <1 ml treated with His-tagged HRV 3C protease [expressed as described previously (*10*)] in the ratio of 1 mg of HRV 3C protease/50 mg of M^Pro^ (by *A*_280_) to cleave the histidine tag. This solution was then dialyzed overnight into HRV3C buffer (50 mM tris, pH 7.0, 150 mM NaCl, 1 mM EDTA, and 1 mM DTT) at room temperature (RT) overnight. RT dialysis reduced precipitation as reported previously (*51*). The dialyzed and cleaved protein was then flown over a Ni-NTA column equilibrated with buffer A. The column was washed with five CV of buffer A and five CV of buffer A with 500 mM imidazole. M^Pro^ was collected and pooled from the flowthrough fractions and the wash with buffer A fractions without imidazole and concentrated using 10 kDa MWCO Amicon ultracentrifugal filters (Millipore Sigma). This protein was then passed over an S200 (GE) column in buffer B [25 mM Hepes, 150 mM NaCl, and 1 mM tris(2-carboxyethyl)phosphine (TCEP), pH 7.5]. Purified M^Pro^ was concentrated to ~10 mg/ml and stored at −80 for months with negligible loss of activity. The variants E166Q, S144A, Q192T, and A173V were generated as previously described (*10*) and purified in the same manner as WT M^Pro^.

### M^Pro^ inhibition assays

All M^Pro^ enzymatic assays used buffer C (50 mM tris, 150 mM NaCl, 1 mM EDTA, 0.05% Tween 20, and 1 mM TCEP, pH 7.4). TCEP was added fresh for each assay and the pH was readjusted to 7.4 upon addition. Enzyme was incubated in buffer C with TCEP for 10 min to ensure full activation of M^Pro^. The compounds were then incubated with M^Pro^ at RT for 1 hour on Corning 3820 384-well black plates. After incubation with compounds, the M^Pro^ substrate, (dR)(dR)(MCA)KATVQAIAS(DNP)K, which was synthesized as previously described (*10*), was used to initiate the reaction at a final concentration of 10 μM. The final dimethyl sulfoxide (DMSO) in each well was 1%. Increase in fluorescence with an excitation of 328 nm and an emission of 393 nm over time was monitored for the first 30 min of the reaction with a BioTek Neo2 plate reader. Data are shown as mean values from experiments performed in at least technical triplicate. Each slope was normalized to M^Pro^ with DMSO only as control. These dose-response curves were fitted to a four-parameter inhibitor versus response curve (IC_50_) curve in GraphPad Prism 10.2.0. For compounds that displayed an IC_50_ value > ~1 μM, 50 nM M^Pro^ was used in the assay. For compounds that displayed greater potency, 25 nM M^Pro^ was used as enzyme concentrations lower than 25 nM resulted in high noise. All mutated SARS-CoV-2 IC_50_ assays were performed at 50 nM. SARS-CoV-1 M^Pro^ IC_50_ values were measured using the same substrate and buffer conditions as the SARS-CoV-2 M^Pro^ assay.

### Inhibitor kinetics assays

A 12-point serial dilution (starting either 1.5 dilution from 3 μM or 1.2 dilution from 100 nM) of inhibitor and DMSO only was prepared in DMSO and diluted in buffer C to 3× the final concentration. This was added to an equal volume of a 3× solution of substrate (30 μM) diluted in buffer C. Ten microliters of a 3× solution of M^Pro^ diluted in buffer C was then add to a Corning 3820 384-well black plate. Both the substrate and inhibitor mixture and the enzyme on the plate were allowed to warm at 37°C for 30 min to ensure accuracy of reads and full activation of M^Pro^. The substrate and inhibitor mixture was then added to the plate and the increase in fluorescence was monitored with an excitation of 328 nm and an emission of 393 nm on a BioTek Neo2 plate reader. All assays were run in technical quintuplicate. An average trace of blank containing substrate alone was then subtracted from all traces. The traces were then fit similar to previous reported (*21*) in GraphPad Prism software version 9.1.1 using *Y* = {[(*V*)/*k*]*[1 − exp(−*k*_app_*x)]} + *Z* to obtain *k*_app_ at each inhibitor concentration. These values were then plotted versus inhibitor concentration and fit using the Michaelis Menten fitting equation in Prism to obtain *k*_inact_ and *K*_I._ All inhibitors tested were compared against 25 nM due to high noise and low signal with lower concentrations of M^Pro^, and thus, for **AVI-4773**, **AVI-4692**, and **AVI-4694**, inhibitor saturation could not be observed and the resulting *k*_app_ data were fit using a linear regression in Prism to obtain the *k*_inact_/*K*_I_ values from the slope. The values were corrected for substrate in the assay using previously described equations (*21*).

### Enzyme aggregation inhibition assays

Samples were prepared in 50 mM KPi buffer, pH 7.0, with a final DMSO concentration at 1% (v/v). Compounds were incubated with 2 nM malate dehydrogenase (MDH) (Sigma-Aldrich, 442610) or AmpC β-lactamase (AmpC) for 5 min. MDH reactions were initiated by the addition of 200 μM nicotinamide adenine dinucleotide (Sigma-Aldrich, 54839) and 200 μM oxaloacetic acid (Sigma-Aldrich, 324427). The change in absorbance was monitored at 340 nm for 80 s. AmpC reactions were initiated by the addition of 50 μM CENTA chromogenic substrate (Sigma-Aldrich, 219475). The change in absorbance was monitored at 405 nm for 80 s. Initial rates were normalized with the DMSO control to determine percent enzyme activity (%). Each compound was initially screened at 10 μM in triplicate. Compounds that did not inhibit MDH but formed colloidal-like particles by DLS were screened against AmpC. Data were analyzed using GraphPad Prism software version 9.1.1 (San Diego, CA).

### Dynamic light scattering

Samples were prepared in filtered 50 mM KPi buffer, pH 7.0, with a final DMSO concentration at 1% (v/v). Colloidal particle formation was detected using DynaPro Plate Reader III (Wyatt Technologies). All compounds were screened in triplicate at 10 μM. If colloidal-like particles were detected, seven-point half-log dilutions of compounds were performed in triplicate. As previously reported (*52*), CACs were determined. Analysis was performed with GraphPad Prism software version 9.1.1 (San Diego, CA).

### Intact protein mass spectrometry

M^Pro^ (500 nM) was incubated with DMSO only and 100 μM **AVI-4516** or **AVI-4694** in 50 mM ammonium acetate and 1 mM TCEP, pH 7.4, with a final DMSO concentration of 1% for 24 hours. Eight microliters of this reaction was then injected onto an I-Class Acquity UPLC (Waters) equipped with an Acquity UPLC protein BEH C4 column (Waters). Mass spectra were measured by a Xevo G2-XS Quadrupole Time of Flight mass spectrometer with a ZSpray ion source. The gradient and mass spectrum collection were performed as described previously (*53*). For comparison of modified proteins, the spectra were deconvoluted using MaxEnt1 software, and the resulting data were visualized in Prism 10.

### Determination of modified residue for covalent inhibitors 4516 and 4694 using chymotryptic digestion

MPro (10 μM) was incubated in 50 mM ammonium acetate and 5 mM DTT, pH 7.4, for 20 hours at RT with either 100 μM of compound **AVI-4516** or **AVI-4694**. The protein was then denatured with the addition of guanidinium HCl (Sigma-Aldrich) to a final concentration of 1 M and heated at 60°C for 20 min then alkylated with 15 mM iodoacetamide and digested according to the manufacturer’s protocol for chymotrypsin (Promega). These samples were desalted using preequilibrated [3 × 15 μl of 50% acetonitrile (ACN) and 0.2% formic acid then 3 × 15 μl of 0.2% formic acid] Cleanup C18 pipette tips (Agilent) by pipetting 15 μl of the acidified solution 10× to ensure full binding of the peptides to the C18 plug in the tips. The tips were then washed 5× with 15 μl of 0.2% formic acid and lastly with 5 × 15 μl of 50% ACN and 0.2% formic acid eluted into a nonstick 0.5-ml Axygen maximum recovery tube (Corning). This eluant was then evaporated in a speed vac and reconstituted with 15 μl of 0.1% formic acid. Five microliters of each sample was then injected into a PepMap RSLC C18 (Thermo Fisher Scientific ES900) attached to a 10,000-psi nanoACQUITY Ultra Performance Liquid Chromatography System (Waters) followed by a Q Exactive Plus Hybrid Quadrupole-Orbitrap (Thermo Fisher Scientific). The peaks were assigned using PAVA, and the peaks were searched using ProteinProspector for any modification on cysteine.

### Reactivity of AVI-4516 with β-mercaptoethanol

A 4 mM solution of **AVI-4516** was prepared in 0.5 ml of DMSO-d_6_ and analyzed by ^1^H-NMR as *T*_0_. Then, 0.5 ml of 40 mM BME-d_4_ in DMSO-d_6_ was added to the compound and incubated at RT. The sample was analyzed by ^1^H-NMR after 1 and 24 hours. The three obtained spectra were aligned and then stacked for comparison.

### Parallel artificial membrane permeability assay

PAMPA measurements were made using the PAMPAExplorer kit (pION, PN 120670-10) as described by the manufacturer. Prisma HT Buffer (pION, PN110151) at pH 7.4 was added to each well of the 96-well High Sensitivity UV Plate (pION, PN 110286) and the UV absorption was read from 250 to 500 nm using 10-nm steps using the Molecular DevicesFlexStation 3 Multi-Mode Microplate Reader to obtain the baseline signal. Once the compounds were added to the 96-well deep well plate (pION, PN 110023) as directed by the kit, the plate was agitated for 1 hour at 1000 rpm. Afterward, the contents of the plate were transferred to a 96-well filter plate (AcroPrep Advance, PN 8129) with an empty deep well plate underneath and spun down in a centrifuge. The filtered solutions were then transferred to a UV plate and read as described above to determine the initial signal. The PAMPA plate sandwich was then prepared as directed in the kit, with three controls as a reference of permeation speed and DMSO as a blank. All the compounds were done in technical triplicate. The GIT-0 lipid solution (pION, PN 110669) was used to mimic the gastrointestinal tract (GIT) conditions. The control references for the GIT assay were verapamil for high permeability, antipyrine for low/moderate permeability, and ranitidine for low permeability. After 15 min, the wells of the acceptor plate were filled with the acceptor sink buffer for the gut PAMPA as described in the kit. The sandwich was then placed in a humidity-controlled chamber and incubated at RT for 18 hours without any well stirrers. After the 18-hour incubation, the contents from the plates were transferred to the UV plate. The permeation speed was then determined in the PAMPA Explorer software using the initial, baseline, and final measurements for each well.

### Cell cytotoxicity assay

A549-ACE2^h^ cells were used for the cytotoxicity assay. Briefly, 2 × 10^4^ cells per well were seeded in Nunc Edge 2.0 96-well plates (Thermo Fisher Scientific) filled with 1.5 ml of phosphate-buffered saline (PBS) for outer moats and 100 μl for in-between wells and incubated for 24 hours at 37°C and 5% CO_2_. Next, cells were treated with compounds at the respective concentrations and vehicle control for 50 hours at 37°C and 5% CO_2_. After the incubation, Cell Titer-Glo reagent was added 1:1 to cells and incubated at RT for 5 min before the transfer of 100 μl of mixture to a white 96-well plate. Luciferase was measured in an infinite M Plex plate reader (Tecan). Cell viability was analyzed as the percentage of viability normalized to the vehicle control. Compound cytotoxicity was assessed in parallel to infection experiments with cells of the same passage.

Compound plates were created using an Echo acoustic dispenser with a final DMSO concentration of 0.5% in a 782080 Greiner 384-well plate. A total of 2000 A540 cells in 25 μl of media were added to each well. The plate was incubated at 37°C under a 5% CO_2_ atmosphere for 48 hours followed by the addition of 25 μl of Cell-Titer glo. Percent viability was measured using a PerkinElmer Envision plate reader. Data processing was completed using GraphPad Prism software version 9.1.1 with a 4-PL logistic curve fit with DMSO only control set as 100% viability and media only control as 0%.

### Cells and viruses

A549-ACE2^h^ cells were generated by stable expression and selection for hACE2 expression (*54*) followed by sorting of cells expressing high levels of the receptor by FACS using a hACE-2 Alexa Fluor 647–conjugated mab (FAB9332R, R-D Systems). Cells were maintained with Dulbecco’s modified Eagle’s medium (DMEM) supplemented with 10% fetal bovine serum (FBS), blasticidin (10 μg/ml) (Sigma), 1× non-essential amino acids (NEAA) (Gibco), and 1% l-glutamine (Corning) at 37°C and 5% CO_2_. Vero-ACE2/TMPRRS2 (VAT) (gifted from A. Creanga and B. Graham at NIH) were maintained in DMEM supplemented with 10% FBS, 1× penicillin-streptomycin, and puromycin (10 μg/ml) at 37°C and 5% CO_2_.The mNeonGreen SARS-CoV-2 (icSARS-CoV-2-mNG) was a kind gift from P.-Y. Shi (University of Texas Medical Branch, Galveston). Virus was propagated in VAT cells and viral sequence verified.

### SARS-CoV-2 replicon assay

SARS-CoV-2 single-round infectious particles were generated as previously described with some modifications (*25*). BHK-21 cells were seeded in a 10-cm dish (1 × 10^6^) and were transfected the next day with 10 μg of pBAC SARS-CoV-2 Spike replicon plasmid (WA1, WA1 *nsp5* L50F/E166Q/L167F, or BA.2.86.1), 5 μg of Spike Delta variant plasmid (*55*), and 5 μg of Nucleocapsid R203M plasmid (*56*) using Xtremegene 9 DNA transfection reagent (Sigma-Aldrich). The medium was changed the next day, and the cells were incubated at 37°C and 5% CO_2_. At 70 hours posttransfection, 20K VAT cells in 50 μl of culture medium were mixed with 50 μl of compound at 4× final concentration and plated in 96-well tissue culture plates. At 72 hours posttransfection, the supernatant was 0.45 μm filtered and 100 μl was added to each well of compound-treated VAT cells and the cells were incubated for 6 to 8 hours at 37°C and 5% CO_2_. The cells were washed once with 300 μL culture medium and 100 μl of compound containing culture medium was added. The cells were incubated for 24 hours and 50 μl of supernatant was transferred to a white 96-well plate. Promega nanoGlo reagent (50 μl) was added and luminescence was recorded in a Tecan plate reader. Experiments were conducted in two biological replicates.

### In cell drug antiviral screening and dose-dependent curves

Compound antiviral activity was determined using the Incucyte live cell analysis system. A549-ACE2^h^ cells were seeded and incubated as for the cytotoxicity assay. The next day, cells were pretreated with compounds for 2 hours before removal of compounds and infection with the mNeon expressing viruses icSARS-CoV-2-mNG [Multiplicity of Infection (MOI) 0.1], SARS-CoV-2/XBB.1.5 (MOI 0.13), SARS-CoV-2/XBB.1.16 (MOI 1), or SARS-CoV-2/EG.5.1 (MOI 0.13) Cells were infected with 50 μl of viral inoculum for 2 hours before the removal and addition of fresh compounds and controls. Fresh compounds and controls were diluted in DMEM complete (10% FBS, 1% l-glutamine, 1× penicillin-streptomycin, and 1× NEAA) supplemented with Incucyte Cytotox Dye (4632, Sartorius) to control for cell death. After the addition of fresh compounds, infected cells were placed in an Incucyte S3 (Sartorius) and infection/cell death was measured for 48 hours in 1-hour intervals using a 10× objective and capturing three images per well per time point under cell maintenance conditions (37°C, 5% CO_2_). Infection was quantified as Total Green Object Integrated Intensity (Green Calibrated Unit [GCU] × μm^2^/image) with an acquisition time of 300 ms and cell death was quantified as Red Object Integrated Intensity (Red Calibrated Unit [RCU] × μm^2^/image) for 400 ms. Image analysis for measurements was done with the following parameters: Phase, AI confluence segmentation. Green, Top-hat segmentation with a 50 μm radius, GCU threshold of 0.5, and Edge Split On. Red was similar to Green with a 100 μm radius and a threshold of 1 RCU. A 2% spectral unmixing of the red channel into the green one was predefined to prevent signal spillover. Post in-built software analysis, raw data were exported and antiviral efficacy was determined as the percentage of infection normalized to the vehicle control. A positive control (nirmatrelvir, HY-138687, MedChemExpress) at efficacious concentrations and uninfected cells were used as an intra-assay positive and negative control. Unless otherwise stated, experiments were performed in triplicate with three technical replicates. EC_50_ values were calculated using GraphPad Prism 10 (La Jolla, CA, USA) using a dose-response inhibition equation with the nonlinear fit regression model.

### AVI-4303 molecular docking

Noncovalent small-molecule docking was conducted using MOE2022.02 (Molecular Operating Environment, Chemical Computing Group). The ligand-bound M^pro^ structure was protonated using the Protonate3D application, then minimized using the Amber10:Extended Hückel Theory force field (*57*) with a root mean square gradient of 0.1 kcal mol^−1^ Å^−2^. Default values were used for fixing protein atoms farther from 8 Å from the ligand atoms and to tether restraints in the MOE QuickPrep panel. Small molecules were docked into the ligand binding site using the Triangle Matcher method and poses ranked by the London dG scoring function. The poses were then refined by the Induced Fit refinement method and rescored by the generalized Born volume integral/weighted surface area dG scoring function (*58*). The top five scoring poses for each docked compound were retained for further evaluation.

### Pancoronavirus inhibition

In vivo antiviral screening (pan-coronavirus assays) was performed via NIAID’s preclinical services (contract no. 75N93019D00021/75N93023F00001 and SRF no. 2021-1229-003). The general procedure for testing compounds is as follows:

Reduction of virus-induced cytopathic effect (primary CPE assay): Confluent or near-confluent cell culture monolayers of Vero 76 cells (or another appropriate cell line) are prepared in 96-well disposable microplates the day before testing. Cells are maintained in MEM supplemented with 5% FBS. For antiviral assays, the same medium is used but with FBS reduced to 2% and supplemented with gentamicin (50 μg/ml). Compounds are dissolved in DMSO. The test compound is prepared at eight serial half-log10 concentrations, usually 32, 10, 3.2, 1.0, 0.32, 0.1, 0.032, and 0.01 μM. Five microwells are used per dilution: three for infected cultures and two for uninfected toxicity cultures. Controls for the experiment consist of six microwells that are infected and not treated (virus controls) and six that are untreated and uninfected (cell controls) on every plate. A known active drug is tested in parallel as a positive control drug using the same method as is applied for test compounds. On the testing day, the growth medium is removed from the cells and the test compound is applied in 0.1 ml volume to wells at 2× concentration. Virus, normally at a titer that will cause >80% CPE (usually an MOI of 80% CPE for most virus strains) is observed in virus control wells. The plates are then stained with 0.011% neutral red for approximately 2 hours at 37°C in a 5% CO_2_ incubator. The neutral red medium is removed by complete aspiration, and the cells may be rinsed 1× with PBS to remove the residual dye. The PBS is completely removed, and the incorporated neutral red is eluted with 50% Sorensen’s citrate buffer/50% ethanol for at least 30 min. Neutral red dye penetrates into living cells; thus, the more intense the red color, the larger the number of viable cells present in the wells. The dye content in each well is quantified using a spectrophotometer at 540 nm wavelength. The dye content in each set of wells is converted to a percentage of dye present in untreated control wells using a Microsoft Excel–based spreadsheet and normalized based on the virus control. The EC_50_ (virus-inhibitory) and 50% cytotoxic (CC_50_, cell-inhibitory) concentrations are then calculated by regression analysis. The quotient of CC_50_ divided by EC_50_ gives the SI_50_ value. Compounds showing an EC_50_ ≥ 5 are considered minimally active. Reduction of virus yield (VYR assay) active compounds are further tested in a confirmatory VYR assay. This assay is run for compounds that have an EC_50_ < 10 μM and SI_50_ ≥ 5. After sufficient virus replication occurs (generally 3 days for many viruses), a sample of supernatant is taken from each infected well (replicate wells are pooled) and held frozen at −80°C for later virus titer determination. After maximum CPE is observed, the viable plates are stained with neutral red dye. The incorporated dye content is quantified as described above to generate the EC_50_ and CC_50_ values. The VYR test directly determines how much test compound is required to inhibit 90% virus replication. The virus yielded in the presence of the test compound is titrated and compared to virus titers from the untreated virus controls. The viral samples (collected as described in the paragraph above) are titrated by the endpoint dilution. Serial 10-fold dilutions of supernatant are made and plated into four replicate wells containing fresh cell monolayers of Vero 76 cells. Plates are then incubated, and cells are scored for the presence or absence of the virus after distinct CPE is observed, and the CCID_50_ is calculated using the Reed-Muench method. The EC_90_ is calculated by regression analysis by plotting the log10 of the inhibitor concentration versus log10 of the virus produced at each concentration. Dividing EC_90_ by the CC_50_ gives the SI_90_ value for this test (*59*). The positive control compound for SARS-CoV-1 and MERS-CoV-1 infection was remdesivir. The positive control for Alpha 229E CoV, Beta OC43, and SARS-CoV-2 strains was EIDD-1931.

### M^Pro^ bioinformatic analyses

To determine whether our chemical matter has the potential of pan-coronavirus activity beyond what is tested biochemical, the contacts that are observed in the crystal structure were inspected for conservation across coronavirus M^Pro^ sequences. A sequence similarity network was constructed using the enzyme function institute online tool using the BLAST option from the 229E MPro sequence (*60*). Starting from a non–SARS-CoV-2 sequence was chosen to not have a large bias toward SARS-CoV-2 genomes. The 10,000-sequence limit was selected, and the network was constructed using a 95% representative node network with default settings. The SSN was visualized in Cytoscape 3.10.3 to determine that an alignment score of 1500 was appropriate to separate the four major coronaviruses grouped into separate clades through UniProt automatic annotations.

The representative sequences (95% ID) for each coronavirus family cluster were then aligned using clustal omega (*61*) with default settings. Because the results from the bioinformatic search were for the polyproteins, the sequences were then manually trimmed by aligning to SARS-CoV-2 Mpro in Aliview 1.28 and all residues that do not align with the cleaved MPro sequence were deleted. These alignments were then uploaded using Skylign (*62*) to generate logos with the following options: observed counts and all information content. The positions that corresponded to residues that make contacts with the compounds were then visually inspected across each family.

### Peptidase selectivity panel

Peptidase was selectively tested using NIH PCS services contract no. HHSN272201800007I/75N93022F00001. Eurofins completed this analysis under the NIH contract. Compounds were screened against a panel of ~30 mammalian serine and cysteine peptidases. First, compounds were tested at a single concentration of 10 μM and IC_50_ was determined in follow-up for assays where the compound displayed >50% inhibition at 10 μM. These data were then visualized in GraphPad Prism 10 and all negative values were set to 0. The full protein name, species, source, and substrate used in the assay are listed in the Supplementary Materials in Table S13 via the information available from Eurofins.

### Secondary pharmacology screening

Secondary pharmacology screening was selectively tested using NIH PCS services contract no. HHSN272201800007I/75N93022F00001. In vitro assays were performed against a panel of ~50 mammalian receptors and enzymes to assess potential off-target pharmacology that might lead to toxicity (*63*). Eurofins completed this analysis under the NIH contract. Test compound was initially measured at a single concentration of 10 μM to determine % inhibition relative to controls. Follow-up IC_50_ analysis was done where the compound exhibited >50% inhibition using five concentrations of test compound to enable determination of an IC_50_. These data were then visualized in GraphPad Prism 10 and all negative values were set to 0. The full protein name, species, source, and substrate used in the assay are listed below in a table via the information available from Eurofins.

### Crystallography

Apo crystals of SARS-CoV-2 M^Pro^ WT and mutants were obtained via vapor diffusion in sitting drops using Swiss 24-well plates using a concentration of 8 mg/ml in 150 mM NaCl, 20 mM tris, pH 7.5, and 0.5 mM TCEP mixed with the well solution containing 20 to 24% w/v polyethylene glycol 8000 and 100 mM tris, pH 7.4. Plates were incubated at 20°C and crystals grew in 3 to 4 days. For crystallization with compounds, proteins were incubated for 1 hour with 10-fold IC_50_ of the compound and trays were prepared in the same conditions as the apo crystals. For some compounds, several rounds of seeding were required to obtain well-diffracting crystals. An initial seeding with crushed apo protein crystals was performed to obtain small crystals to subsequently soak with compound to generate crystalline protein-inhibitor complexes. These small crystals were then used for a second round of seeding yielding crystals appropriate for data collection. The crystals grown after the second round of seeding were then dipped into cryoprotectant containing the well buffer, 20% glycerol, and 100 μM of compound before being flash frozen in liquid nitrogen. X-ray diffraction data were collected at beamline 8.3.1 at the Advanced Light Source.

### Crystallographic data refinement

All datasets were refined using the same pipeline. Diffraction patterns were indexed, integrated, and scaled with XDS refined using the same pipeline. The data were indexed, integrated, and scaled with XDS (*64*). The high-resolution limit for each dataset was chosen based on three parameters, CC1/2 > 30% (*14*), *I* Mean *I*/σ(*I*) > 1, and completeness > 90%, for the highest-resolution shell. The structure determination and refinement were performed with Phenix 1.20.1_4487 (*65*). Structures were first modeled by molecular replacement using the starting model of *nsp5* PBD: 8B2T and refined (with phenix.refine) using three refinement macrocycles without ligands to generate a difference density for the ligand. The R-free flags were generate during the first phenix.refine and further processing was performed with the file “data.mtz.” For all the ligands, the ligand restraints were generated from SMILES strings using phenix.elbow (*66*) and ligands were placed into the positive peaks in the *mF*_o_ − *F*_c_ difference density using COOT version 0.9.8.93 (*67*). For covalent ligands, **AVI-4516** and **AVI-4692**, once binding pose was identified as above, a new restraints file was generated describing covalently linked compound to the cys side chain, which was then linked through “LINK” on COOT command to the c-alpha carbon. This allowed specification of the proper geometry for the covalent link to the sp2 carbon in the dictionary .cif file. Every ligand-protein costructure was further refined with phenix.refine using five refinement macrocycles. For the compound **AVI-4692**, positive peaks in the *mF*_o_ − *DF*_c_ difference density maps were observed near the cysteine, suggesting an oxidized cysteine. The oxidized cysteine (CSO) was added to the model using COOT and assigned to the alternative occupancy identifier (altloc) A and the ligand-bound state was assigned to altloc B. Coordinates and occupancy of the CSO and compound-bound were refined using phenix.refine and the maps and coordinates were manually examined using COOT. On the basis of positive peaks in the *mF*_o_ − *DF*_c_ difference density map after refinement around the compound, and the fact that noncovalent versions of these compounds still bind, we inferred the presence of unbound compound at partial occupancy. The unbound compound was added to the positive peaks in the *mF*_o_ − *DF*_c_ difference density and assigned to altloc C using COOT. Coordinates and occupancy were refined using *phenix.refine*. After a manual inspection of the map and coordinates, the occupancy of CSO, bound **AVI-4692**, and free **AVI-4692** was 76, 24, and 71%, respectively. The waters were automatically added to the positive peak in the *mF*_o_ − *DF*_c_ difference density map >3.5σ using phenix.refine at the end of the refinement. Last, after one round of refinement, maps and coordinates were manually examined using COOT. Statistics for the refined structures are reported in table S10. The crystallography datasets have been deposited in the PDB under the deposition IDs 9MVM (**AVI-3318**), 9MVQ (**AVI-4303**), 9MVP (**AVI-4516**), and 9MVO (**AVI-4692**).

### Drug synergy assessment

Synergy experiments are based on the overlap method (*68*). Briefly, 2 × 10^4^ A549-ACE2^h^ cells per well were seeded on Edge 2.0 96-well plate and incubated for 24 hours at 37°C and 5% CO_2_. The next day, a dilution matrix plate was generated by serially diluting the input compound and test compound to a 2× final concentration. Subsequently, cell culture medium was removed from cells and fresh medium was mixed with drug combinations to a 1× final concentration and pretreated on the cells or 2 hours at 37°C and 5% CO_2_. Treated cells were washed and infected for 2 hours with SARS-CoV-2 mNeon WA1 or mNeon XBB.1.16 at a MOI of 0.1 or 1, respectively. After that, virus inoculum was removed and 1× drug combinations were added to the cells. Infection was measured using the Incucyte technology as previously described. Synergy analysis was performed using Synergyfinder 3.0 (*69*) applying the ZIP method (35580060). 

### Kinetic solubility

Kinetic solubility, microsomal stability, MDCK-MDR1 bidirectional transport assay, PPB assay, and CYP 450 inhibitions studies were conducted at Quintara Discovery (Hayward, CA, USA). Kinetic solubility of drug substances in various buffer systems can be determined using samples supplied in DMSO solution. A sample dissolved in DMSO (typically 10 mM) is diluted with the appropriate amount of buffer (typically PBS, pH 7.4) and mixed by shaking for 1.5 hours followed by vacuum filtration. The sample is then assayed via reversed-phase high-performance liquid chromatography (HPLC) with UV detection. Quantitation is achieved by the reference to a three-point standard curve constructed via serial dilution of drug substance dissolved in 100% DMSO. Reference compounds (such as testosterone) are included in each test. Each compound was sent as a DMSO stock. DMSO stocks and control compounds (such as testosterone) are thawed. Add 190 μl of buffer solution (PBS, pH 7.4 as the default buffer) to all wells on a 96-well Millipore Solubility filter plate. Transfer 10 μl of compound DMSO stocks in triplicate to the buffer wells to a final concentration of 500 μM. The filter plate is shaken for 1.5 hours at RT. Samples are filtered via a vacuum system into a fresh 96-well plate. Dilute compounds to 500 μM (highest concentration) in DMSO and further dilute them 1:10 for calibration curve (three-point) using an HPLC/UV analysis (220, 254, and 280 nm).

### Microsomal stability assay

Metabolic stability of testing compound can be evaluated using human, rat, mouse, or other animal liver or intestine microsomes to predict intrinsic clearance. The assay is carried out in 96-well microtiter plates at 37°C. Reaction mixtures (25 μl) contain a final concentration of 1 μM test compound, liver microsome protein (0.5 mg/ml), and 1 mM NADPH and/or 1 mM UDPGA (with alamethicin) in 100 mM potassium phosphate, pH 7.4 buffer with 3 mM MgCl_2_. The incubation is done with *N* = 2. At each of the time point examples, 0, 15, 30, and 60 min, 150 μl of quench solution (100% acetonitrile with 0.1% formic acid) with internal standard is transferred to each well. Besides the zero-minute controls, mixtures containing the same components except the NADPH can also be prepared as the negative control. Verapamil is included as a positive control to verify assay performance. Plates are sealed, vortexed, and centrifuged at 4°C for 15 min at 4000 rpm. The supernatant is transferred to fresh plates for liquid chromatography with tandem mass spectrometry (LC/MS/MS) analysis. All samples are analyzed on LC/MS/MS using an AB Sciex API 4000 instrument, coupled to a Shimadzu LC-20 AD LC Pump system. Analytical samples are separated using a Waters Atlantis T3 dC18 reversed-phase HPLC column (20 mm by 2.1 mm) at a flow rate of 0.5 ml/min. The mobile phase consists of 0.1% formic acid in water (solvent A) and 0.1% formic acid in 100% acetonitrile (solvent B). The extent of metabolism was calculated as the disappearance of the test compound, compared to the 0-min time incubation. Initial rates are calculated for the compound concentration and used to determine *t*_1/2_ values and, subsequently, the intrinsic clearance, CL_int_ = (0.693)(1/*t*_1/2_ (min)(g of liver/kg of body weight)(ml incubation/mg of microsomal protein)(45 mg of microsomal protein/g of liver weight).

### MDCK-MDR1 bidirectional transport assay

MDCK-MDR1 cells are plated into 96-well Millipore Millicell-96 plates at 7500 cells/75 μl per well and incubated for 3 days at 37°C with 5% CO_2_. Cells are washed with Hanks’ balanced salt solution (HBSS) with 5 mM Hepes for 30 min before starting the experiment. Test compound solutions are prepared by diluting DMSO stock into HBSS buffer, resulting in a final DMSO concentration of 0.1%. Before the experiment, cell monolayer integrity is verified by transendothelial electrical resistance. Transport experiment is initiated by adding test compounds to the apical (75 μl) or basal (250 μl) side.

Transport plates are incubated at 37°C in a humidified incubator with 5% CO_2_. Samples are taken from the donor and acceptor compartments after 1 hour and analyzed by LC/MS/MS. Digoxin is typically used as reference control. Apparent permeability (*P*_app_) values are calculated using the following equation

$$P_{\text{app}}=\left(dQ/dt\right)/A/C_{0}$$

where d*Q*/d*t* is the initial rate of amount of test compound transported across the cell monolayer, *A* is the surface area of the filter membrane, and *C*_0_ is the initial concentration of the test compound, calculated for each direction using a 4-point calibration curve by LC/MS/MS.

Net flux ratio between the two directional transports is calculated by the following equation

$$\text{Ratio}=P_{\text{app,}}{}_{B-A}/P_{\text{app,}}{}_{A-B}$$

where *P*_app,B–A_ and *P*_app,A–B_ represent the apparent permeability of test compound from the basal-to-apical and apical-to-basal side of the cellular monolayer, respectively. Recovery is calculated on the basis of the compound concentration at the end of the experiment, compared to that at the beginning of the experiment, adjusted for volumes. A net flux ratio greater than 2 is considered a positive result for substrate determination.

### PPB assay

The rapid equilibrium dialysis (RED) device inserts along with a Teflon base plate (Pierce, Rockford, IL) are used for the binding studies. Human or animal plasma is obtained commercially. The pH of the plasma is adjusted to 7.4 before the experiment.

DMSO stocks (1 mM) are spiked into the plasma to make a final concentration of 2 μM. Aliquots of 100 μl were transferred to a fresh 96-well deep-well plate as the T4 (recovery) samples. An equal volume of blank PBS buffer is added to the plate to make the matrix as 50:50 plasma:buffer. The T4 recovery samples are incubated at 37°C for 4 hours. The spiked plasma solutions (300 μl) were placed into the sample chamber (indicated by the red ring), and 500 μl of PBS buffer, pH 7.4, is placed into the adjacent chamber. The plate is sealed with a self-adhesive lid and incubated at 37°C on an orbital shaker (250 rpm) for 4 hours. After 4 hours, from the RED plate, aliquots (100 μl) are removed from each side of the insert (plasma and buffer) and dispensed into the 96-well plate. Subsequently, 100 μl of blank plasma is added to the buffer samples and 100 μl of blank buffer is added to all the collected plasma samples. Last, 300 μl of quench solution (50% acetonitrile, 50% methanol, and 0.05% formic acid, warmed up at 37°C) containing internal standards is added to each well. Plates are sealed, vortexed, and centrifuged at 4°C for 15 min at 4000 rpm. The supernatant is transferred to fresh plates for LC/MS/MS analysis. Reference compound propranolol was included in every experiment. All samples were analyzed on LC/MS/MS using an AB Sciex API 4000 instrument, coupled to a Shimadzu LC-20AD LC Pump system. Analytical samples are separated using a Waters Atlantis T3 dC18 reversed-phase HPLC column (20 mm by 2.1 mm) at a flow rate of 0.5 ml/min. The mobile phase consisted of 0.1% formic acid in water (solvent A) and 0.1% formic acid in acetonitrile (solvent B).

The percentage of test compound bound to protein is calculated by the following equation: % Free = (Concentration in buffer chamber/Concentration in plasma chamber) × 100%. % Bound = 100% − % Free. The percentage of test compound recovered was calculated by the following equation: % Recovery = (Concentration in buffer chamber*500 + Concentration in plasma chamber*300)/(Concentration in T4 sample*300) × 100%. All the samples are diluted by quench solution to around 400 nM to be within compounds’ linear ranges.

### CYP450 inhibition assay

Selective substrates are incubated with pooled HLMs as single substrates. The assays were performed in 384-well plates using a final volume of 40 μl at 37°C. All assays use 100 mM potassium phosphate buffer, pH 7.4, with 3 mM MgCl_2_ and 1 mM cofactor NADPH. Compounds were tested at 10 μM to obtain % inhibition. Human cytochrome-specific inhibitors are also included within each assay as reference compounds. The quantitation window can be defined as 100% enzyme activity (NADPH added) in the presence of vehicle control (DMSO). Analysis was via LC/MS/MS where enzyme activity is based on the detection of appearance of the respective substrate metabolites.

Briefly, the conditions for all tested CYP450 are as follows: For CYP1A2, to HLM (0.1 mg/ml), the substrate phenacetin was used at 30 μM and the metabolite acetaminophen was monitored after 10 min. For CYP2B6, to HLM (0.1 mg/ml), the substrate bupropion was used at 100 μM and the metabolite hydroxybupropin was monitored after 10 min. For CYP2C8, to HLM (0.1 mg/ml), the substrate paclitaxel was used at 2 μM and the metabolite 6α-Hydroxypaclitaxel was monitored after 10 min. For CYP2C9, to HLM (0.1 mg/ml), the substrate diclofenac was used at 4 μM and the metabolite hydroxydiclofenac was monitored after 10 min. For CYP2C19, to HLM (0.2 mg/ml), the substrate mephenytoin was used at 35 μM and the metabolite 4′-hydroxymephenytoin was monitored after 20 min. For CYP2D6, to HLM (0.1 mg/ml), the substrate bufuralol was used at 10 μM and the metabolite hydroxybufuralol was monitored after 10 min. For CYP3A4, to HLM (0.05 mg/ml), either testosterone (30 μM) or midazolam (5 μM) was added and the metabolites 6β-hydroxytestosterone and 1′-hydroxymidazolam were monitored, respectively, after 5 min.

All samples were analyzed on LC/MS/MS using an AB Sciex API 4000 instrument, coupled to a Shimadzu LC-20 AD LC Pump system. Analytical sample of 1A2-ACE is separated using a Thermo Hypersil Gold C18 (50 mm by 2.1 mm) column, and other samples were separated using a Waters Atlantis T3 dC18 reversed-phase HPLC column (20 mm by 2.1 mm) at a flow rate of 0.5 ml/min. The mobile phase consists of 0.1% formic acid in water (solvent A) and 0.1% formic acid in acetonitrile (solvent B).

### TPP assay

For TPP optimization experiments, 5 μM MPro was treated with either DMSO or compound at a final concentration of 0.5, 5, 50, or 100 μM at RT for 30 min. Each condition was split into 20-μl aliquots, and aliquots from each condition were heated for 4 min on a Bio-Rad C1000 Touch Thermal cycler at the following temperatures: 37, 39, 42.3, 46.4, 51.9, 56.1, 59, and 61°C. Samples were centrifuged at 20,000*g* for 60 min. Supernatant was incubated with 8 M urea, 100 mM tris, and 10 mM TCEP/44 mM CAA (pH ~7.5) for 60 min. The urea concentration was diluted to 1 M with 100 mM tris (pH ~7.5). Samples were digested overnight with 1 μl of trypsin (Promega, 0.4 μg/μl). Samples were desalted with a 96-well mini 20MG PROTO 300 C18 plate (HNS S18V, The Nest Group) according to manufacturer’s directions. Peptide concentration was determined by NanoDrop (Thermo Fisher Scientific).

For optimization experiments, peptides were injected onto an Orbitrap Exploris 480 MS system (Thermo Fisher Scientific) equipped with an Easy nLC 1200 system (Thermo Fisher Scientific). Peptides were separated on a PepSep reversed-phase C18 column (1.9 mm particles, 1.5 mm × 15 cm, 150 mm ID) (Bruker). Mobile phase A consisted of 0.1% formic acid and mobile phase B consisted of 80% ACN/0.1% formic acid. Peptide mixtures were separated by mobile phase B ranging from 0 to 28% over 27 min, followed by an increase to 45% B over 4 min, then held at 95% B for 9 min at a flow rate of 500 nl/min. Samples were analyzed by data-dependent acquisition (DDA) with an MS1 resolution of 120 K [at 200 mass/charge ratio (*m*/*z*)], a scan range of 350 to 1250 *m*/*z*, an MS1 normalized AGC target of 300%, and an exclusion duration of 30 s. MS2 cycle time was set to 1 s, with an isolation window of 1.3 *m*/*z*. Samples were fragmented at 28% HCD (higher-energy collisional dissociation) in Auto Scan Range Mode using an AGC target of 200%. MS2 Orbitrap resolution was set to 15,000.

For lysate experiments, pelleted A549 cells were resuspended in extraction buffer [1× PBS + phosphatase inhibitors (phosSTOP; Roche) (no protease inhibitors)] with gentle pipetting followed by rotation at 4°C for 30 min. Lysates were centrifuged at 1000*g* for 10 min at 4°C and supernatant was transferred to fresh tubes. Lysates (two replicates per condition) were distributed into ten 20-μl aliquots in polymerase chain reaction tubes. Samples were heated from 37° to 64°C in 3°C increments on a Bio-Rad C1000 Touch Thermal cycler and held for 4 min at the specified temperature. Samples were held at RT for 3 min. Samples were flash frozen, followed by thawing at 35°C (×2). Aggregated proteins were removed by centrifugation at 20,000*g* for 60 min. Twenty microliters of lysis buffer (8 M urea and 100 mM tris, pH ~7.5) was added to each well and samples were incubated for 30 min at RT. Samples were reduced and alkylated by the addition of TCEP (100 mM final) and 2-chloroacetamide (44 mM final) followed by incubation at RT for 30 min. Urea concentration was diluted to 1 M with 100 mM tris (pH ~7.5). Samples were digested overnight with LysC (Wako, 1:100 enzyme:protein ratio) and trypsin (Promega, 1:50 enzyme:protein ratio). Samples were desalted with a 96-well mini 20MG PROTO 300 C18 plate (HNS S18V, The Nest Group) according to the manufacturer’s directions. Peptide concentration was determined by NanoDrop (Thermo Fisher Scientific).

For lysate experiments, equal amounts of peptides were injected onto a timsTOF SCP (Bruker) connected to an EASY-nLC 1200 system (Thermo Fisher Scientific). Peptides were separated on a PepSep reverse-phase C18 column (1.9 mm particles, 1.5 mm by 15 cm, 150 mm ID) (Bruker) with a gradient of 5 to 28% buffer B (0.1% formic acid in acetonitrile) over buffer A (0.1% formic acid in water) over 20 min, an increase to 32% B in 3 min, and held at 95% B for 7 min. Data-Independent Acquisition (DIA) using Parallel Accumulation-Serial Fragmentation (DIA-PASEF) analyses were acquired from 100 to 1700 *m*/*z* over a 1/Kø of 0.70 to 1.30 Vs/cm^2^, with a ramp and accumulation time set to 75 ms. Library DDA PASEF runs were collected over the same *m*/*z* and 1/Kø range and a cycle time of 1.9 s.

All data were searched against the UniProt Human database (downloaded 25 May 23) appended with the SARS-CoV-2 database (downloaded 20 February 2024) using a combined DDA and DIA library in Spectronaut (Biognosys, version 16.0). Default settings, including trypsin digestion, variable modifications of methionine oxidation and N-termini acetylation, and fixed modification of cysteine carbamidomethylation, were used. Missing values were imputed for each run using background intensity. Data were filtered to obtain a false discovery rate of 1% at the peptide spectrum match and protein level. Lysate experiments were normalized (*70*) and melting points were determined in R using the Inflect package (*71*).

An ion mobility gas-phase fractionation library was acquired based on the work of Penny *et al.* (*72*). Briefly, data were collected from 400 to 1200 *m*/*z* using 5 *m*/*z* windows with a 1 *m*/*z* overlap on each side. The ion mobility range spanned 0.57 to 1.47 V/cm^2^, with two quadrupole positions per ion mobility cycle. These windows were evenly distributed across seven acquisition methods, with each method comprising 15 ion mobility cycles. Data are available via ProteomeXchange (*73*) with identifier PXD058827.

### Mice

All animal use protocols (AN203103-00A) were approved by the Institutional Animal Care and Use Committees at the University of California, San Francisco, and Gladstone Institutes, and were conducted in strict accordance with the National Institutes of Health *Guide for the Care and Use of Laboratory Animals* [National Research Council (US) Committee for the Update of the Guide for the Care and Use of Laboratory Animals, 2011]. The studies involved 6- to 8-week-old female WT mice (the Jackson Laboratory, 000664). The mice were housed in a pathogen-free facility with controlled temperature and humidity, a 12-hour light/dark cycle, and ad libitum access to water and standard laboratory rodent chow.

### SARS-CoV-2 culture for mice studies

The SARS-CoV-2 Beta variant was used for all the mice infection studies. All live virus experiments were performed in a Biosafety Level 3 laboratory. SARS-CoV-2 stocks were propagated in Vero-ACE2-TMPRSS2 cells, and their sequence was verified by next-generation sequencing. Viral stock titer was calculated using plaque-forming assays.

### Antiviral screening of compounds in WT mice

Forty-five WT mice were infected with the SARS-CoV-2 Beta variant at a dose of 10^3^ plaque-forming units (PFU) and divided into three treatment groups: **AVI-4516** (100 mg/kg), vehicle, and nirmatrelvir (300 mg/kg) as a positive control, with each group containing 15 mice. Treatment commenced 4 hours postinfection with oral BID dosing for 5 days, during which the animals were closely monitored for disease parameters such as weight loss, hypothermia, and posture. At 2, 4, and 7 dpi, five animals from each group were euthanized, and their lung tissue was harvested and homogenized for downstream analysis using plaque assay. The left lung lobe tissue from an additional subset of animals were processed for histological observations.

### Evaluating mouse dose-dependent efficacy of AVI-4516

The dose-dependent antiviral efficacy of **AVI-4516** was evaluated in SARS-CoV-2 Beta–infected WT mice. A group of 25 WT female mice aged 6 to 8 weeks were infected with the SARS-CoV-2 Beta variant at 10^3^ PFU. The mice were orally dosed with a range of concentrations of **AVI-4516** starting from 12.5 to 100 mg/kg. The treatment was started at 4 hours postinfection followed by BID on day 1 postinfection. All the mice were euthanized at day 2 postinfection and their lung tissues were harvested to estimate the virus titers in the lungs. Similar experiment was performed to analyze antiviral efficacy of AVI-4773, where the experimental time points were limited to day 2- and 4- post infection with three treatment groups including AVI-4773, vehicle and ensitrelvir (positive control).

### Plaque assays

The lung homogenates were clarified by centrifugation, and the supernatants were serially diluted to infect Vero ACE2-TMPRSS2 cells. Following a 1-hour absorption period, 2.5% Avicel (Dupont, RC-591) was applied to the cells and incubated for 48 hours. After incubation, the Avicel was removed, and the cells were fixed in 10% formalin for 1 hour and then stained with crystal violet for 10 min. Plaques were counted, and the data were presented as PFU.

### Chemical synthesis general procedures

Unless otherwise noted, all chemical reagents and solvents used are commercially available. Air and/or moisture sensitive reactions were carried out under an argon atmosphere in oven-dried glassware using anhydrous solvents from commercial suppliers. Air- and/or moisture-sensitive reagents were transferred via syringe or cannula and were introduced into reaction vessels through rubber septa. Solvent removal was accomplished with a rotary evaporator at ca. 10 to 50 torr. NMR spectra were recorded on a Bruker Avance III HD 400 MHz spectrometer. Chemical shifts are reported in δ units (ppm). NMR spectra were referenced relative to residual NMR solvent peaks. Coupling constants (*J*) are reported in hertz. Chromatography was carried out using Isolera Four and CombiFlash NextGen 300 flash chromatography systems with Silia*Sep* silica gel and C18 cartridges from Silicycle. Reversed-phase chromatography was carried out on Waters 2535 Separation module with a Waters 2998 Photodiode Array Detector. Separations were carried out on an XBridge Preparative C18, 19 mm by 50 mm column at ambient temperature using a mobile phase of water-acetonitrile containing a constant 0.1% formic acid. LC/MS data were acquired on a Waters Acquity UPLC QDa mass spectrometer equipped with Quaternary Solvent Manager, Photodiode Array Detector, and Evaporative Light Scattering Detector. Separations were carried out with an Acquity UPLC BEH C18 1.7 μm, 2.1 mm by 50 mm column at 25°C, using a mobile phase of water-acetonitrile containing a constant 0.1% formic acid. Specific synthetic steps are found in text S1.

## Supplementary Materials

### This PDF file includes:

Supplementary Text Figs. S1 to S20 Tables S1 to S13 NMR Spectra of Final Compounds
