# Supplementary material for: Structure-based discovery of highly bioavailable, covalent, broad-spectrum coronavirus MPro inhibitors with potent in vivo efficacy
Source: Sci Adv. 2025 Apr 23;11(17):eadt7836. doi: 10.1126/sciadv.adt7836 (PMC12017303; doi:10.1126/sciadv.adt7836)
Supplement: Supplementary file 1 — Supplementary Text Figs. S1 to S20 Tables S1 to S13 NMR Spectra of Final Compounds [file sciadv.adt7836_sm.pdf]

Science Advances
AAAS

# Supplementary Materials for

## Structure-based discovery of highly bioavailable, covalent, broad-spectrum coronavirus $M^{Pro}$ inhibitors with potent in vivo efficacy

Tyler C. Detomasi *et al.*

Corresponding author: Melanie Ott, melanie.ott@gladstone.ucsf.edu; Adam R. Renslo, adam.renslo@ucsf.edu; Charles S. Craik, charles.craik@ucsf.edu

*Sci. Adv.* **11**, eadt7836 (2025)
DOI: 10.1126/sciadv.adt7836

**This PDF file includes:**

Supplementary Text
Figs. S1 to S20
Tables S1 to S13
NMR Spectra of Final Compounds

**Supplementary Text 1**: Extended methods for synthesized chemicals.

### General Procedure for the Synthesis of Trisubstituted Uracil Derivatives

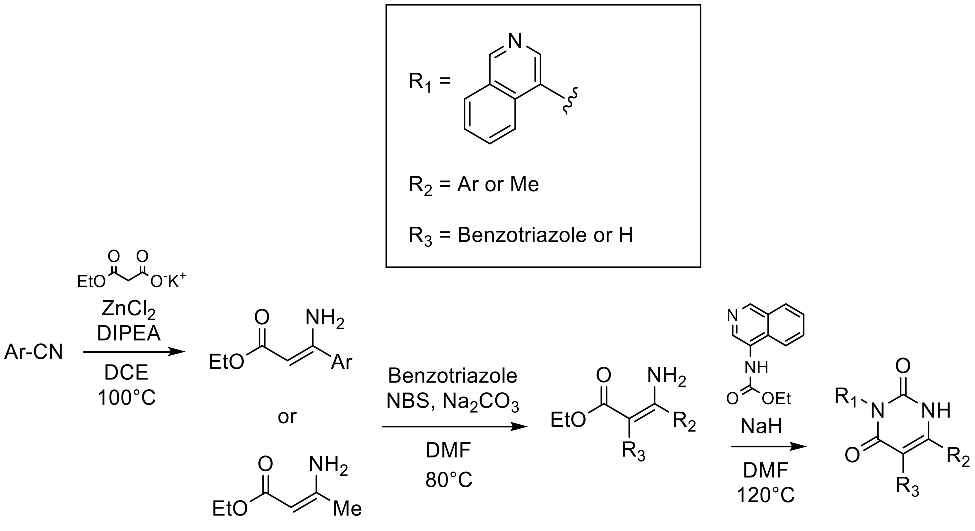


### General Procedure for the Synthesis of Tetrasubstituted Uracil Derivatives

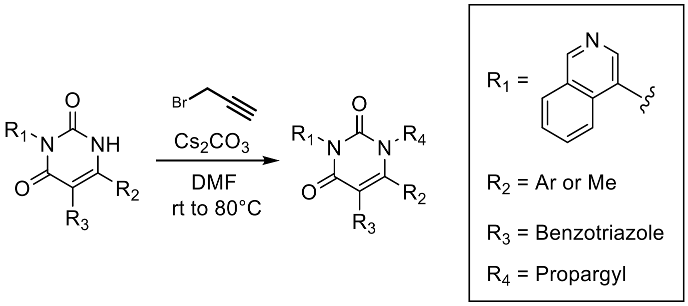


### Synthesis of carbamic acid-4-isoquinolinyl-ethyl ester

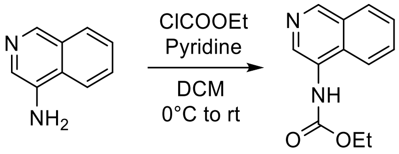


Pyridine (1.7 mL, 20.8 mmol, 3 eq) was added to a suspension of 4-aminoisoquinoline (1 g, 6.94 mmol) in DCM (25 mL) at 0°C, followed by a dropwise addition of ethyl chloroformate (0.995 mL, 10.41 mmol, 1.5 eq) dissolved in 5 mL of DCM. Then the mixture was warmed to room temperature and stirred for 1 h and quenched with 1N HCl (20 mL). The aqueous phase was extracted with DCM (3x20 mL). The organic phase was dried over $Na_2SO_4$ and concentrated in vacuo. The crude material (1 g, 6.02 mmol, 87%) was used in the next step without further purification.
**General**: $C_{12}H_{12}N_2O_2$; MW = 216.24.
**LCMS (ESI)**: $m/z$ = 217.1 $[M+H]^+$.

**General Procedure A: Blaise Reaction of Aryl Nitriles**

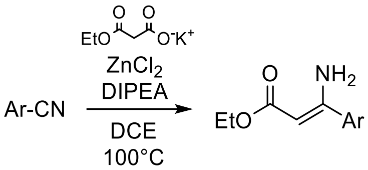


To a solution of the aryl nitrile (1 mmol) in 1,2-dichloroethane (10 mL), were added $ZnCl_2$ (1.2 eq), potassium ethyl malonate (2.3 eq) and DIPEA (0.3 eq). The mixture was stirred at 100°C for 16 h, then cooled to room temperature and washed with saturated $NH_4Cl$ aqueous solution. The aqueous phase was extracted with DCM and the organic extracts were dried over $Na_2SO_4$ and concentrated in vacuo. The crude material was used in the next step without further purification.

**General Procedure B: Amination of Enaminones**

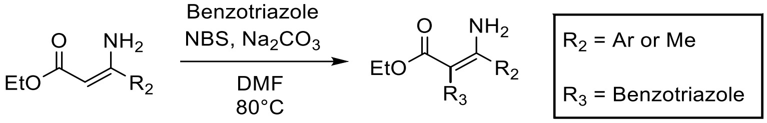


*N*-Bromosuccinimide (1.2 eq) was added to a solution of enaminone (1 mmol) in DMF (2 mL) and the mixture was stirred for 10 min at room temperature. Then, the corresponding benzotriazole (1.2 eq), and $Na_2CO_3$ (1.2 eq) were added, and the mixture was heated to 80°C and stirred for 2 h. The reaction was quenched with saturated $Na_2S_2O_3$ aqueous solution, and the aqueous phase was extracted with EtOAc. The organic extracts were dried over $Na_2SO_4$ and concentrated in vacuo. The crude was purified by flash chromatography on silica gel (EtOAc in hexane 0% to 60%).

**General Procedure C: Synthesis of Uracil Analogs**

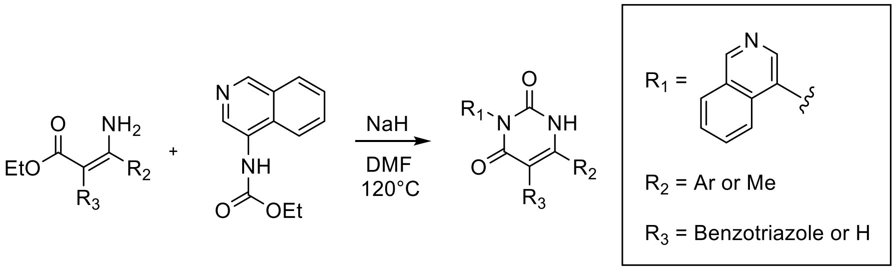


The enaminone (0.2 mmol) was dissolved in DMF (1 mL) and added dropwise to a suspension of NaH (60% in mineral oil; 2.5 eq) in DMF (0.5 mL) at 0°C. The mixture was stirred for 30 min at 0°C and then added to a solution of carbamic acid-4-isoquinolinyl-ethyl ester (1.5 eq) in DMF (1 mL) at 0°C. The mixture was heated to 120°C and stirred for 2 h; then cooled to room temperature and directly purified by preparative HPLC.

**General Procedure D: Alkylation of Uracil**

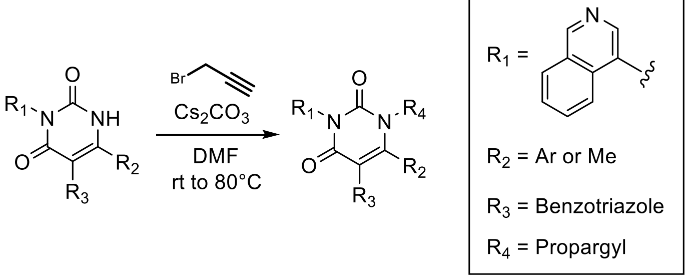


The uracil starting material (0.3 mmol) was dissolved in DMF (3 mL), then $Cs_2CO_3$ (1.7 eq) and propargyl bromide (1.5 eq) were added at room temperature. The mixture was stirred at 80°C for 4 h, and directly purified by preparative HPLC (40% to 90% $CH_3CN$ in $H_2O$ + 0.1% formic acid).

**Synthesis of AVI-4301**

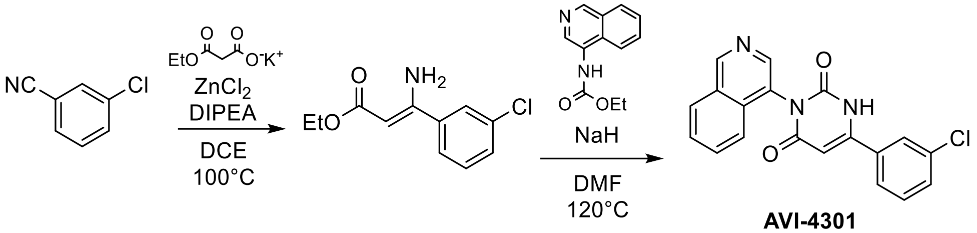

**AVI-4301**

**Step 1: Ethyl 3-amino-3-(3-chlorophenyl)-2-propenoate**

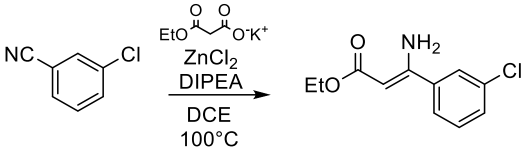

The general procedure A (Blaise Reaction) was followed, using 3-chlorobenzonitrile (1 g, 7.27 mmol). Yield: 1.52 g, 6.75 mmol, 93%.
**General**: $C_{11}H_{12}ClNO_2$; MW = 225.67.
**LCMS (ESI)**: *m/z* = 226.1 $[M+H]^+$ (calcd for $C_{11}H_{13}ClNO_2$: 226.06).

**Step 2: AVI-4301**

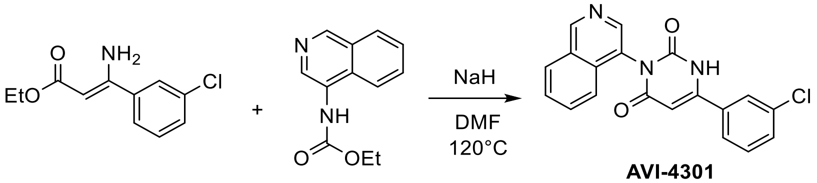

**AVI-4301**

General Procedure C (Synthesis of Uracil) was followed using ethyl 3-amino-3-(3-chlorophenyl)-2-propenoate (25 mg, 0.111 mmol). Purification by preparative HPLC (20% to 70% $CH_3CN$ in $H_2O$ + 0.1% formic acid) afforded **AVI-4301** (13.5 mg, 0.0387 mmol, 35%) as a white solid.
**General**: $C_{19}H_{12}ClN_3O_2$; MW = 349.77.
**$^1$H-NMR** (400 MHz, $CDCl_3$): δ (ppm): 10.82 (brs, 1H); 9.36 (s, 1H); 8.51 (s, 1H); 8.11 (m, 1H); 7.74 (m, 1H); 7.68 (t, *J* = 5.6 Hz, 2H); 7.57 (t, *J* = 1.8 Hz, 1H); 7.42 (dd, *J* = 14.5, 8.0 Hz, 1H); 7.26 (s, 1H); 7.16 (t, *J* = 7.9 Hz, 1H); 6.17 (s, 1H).
**LCMS (ESI)**: *m/z* = 350.1 $[M+H]^+$ (calcd for $C_{19}H_{13}ClN_3O_2$: 350.06).

**Synthesis of AVI-4303**

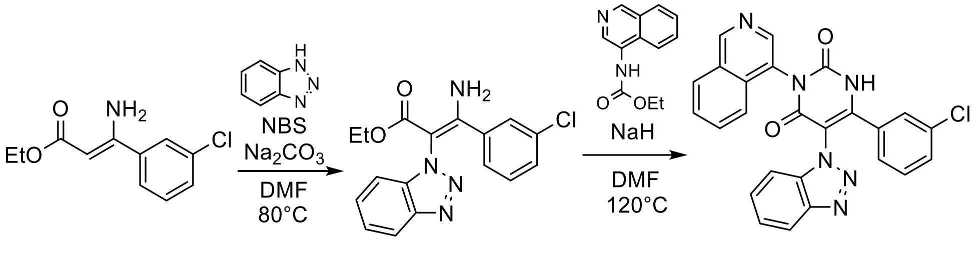

**AVI-4303**

**Step 1: Ethyl 3-amino-2-(1H-benzo[d][1,2,3]triazol-1-yl)-3-(3-chlorophenyl)acrylate**

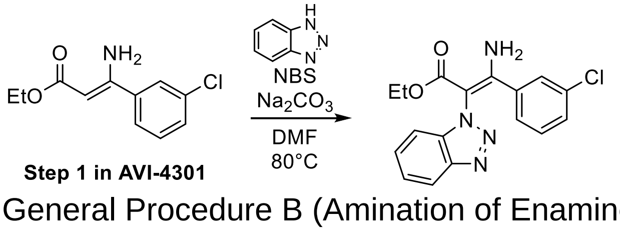

General Procedure B (Amination of Enaminone) was followed using ethyl 3-amino-3-(3-chlorophenyl)-2-propenoate (200 mg, 0.886 mmol) and 1*H*-benzotriazole. Yield: 80 mg, 0.233 mmol, 26%.

**General**: $C_{17}H_{15}ClN_4O_2$; MW = 342.78.
**LCMS (ESI)**: *m/z* = 343.1 $[M+H]^+$ (calcd for $C_{17}H_{16}ClN_4O_2$: 343.09).

### Step 2: AVI-4303

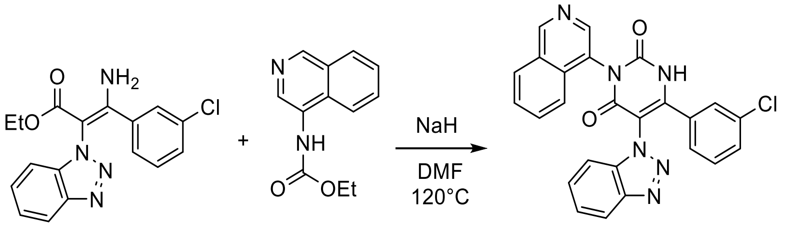


**AVI-4303**

General Procedure C (Synthesis of Uracil) was followed using ethyl 3-amino-2-(1H-benzo[d][1,2,3]triazol-1-yl)-3-(3-chlorophenyl)acrylate (122 mg, 0.357 mmol). Purification by preparative HPLC (30% to 70% $CH_3CN$ in $H_2O$ + 0.1% formic acid) afforded **AVI-4303** (55 mg, 0.118 mmol, 33%) as a white solid.
**General**: $C_{25}H_{15}ClN_6O_2$; MW = 466.89.
**$^1$H-NMR** (400 MHz, DMSO-d6): δ (ppm): 12.56 (brs, 1H); 9.44 (s, 1H); 8.65 (s, 1H); 8.28 (d, *J* = 8.3 Hz, 1H); 8.26-8.04 (m, 1H); 8.01 (d, *J* = 8.3 Hz, 1H); 7.96-7.82 (m, 2H); 7.79 (t, *J* = 7.6 Hz, 1H); 7.60-7.51 (m, 2H); 7.43 (dt, *J* = 7.7, 1.7 Hz, 1H); 7.38 (t, *J* = 7.7 Hz, 1H); 7.34-7.17 (m, 2H).
**$^{13}$C-NMR** (101 MHz, DMSO-d6): δ (ppm): 160.3, 153.4,152.3, 150.5, 144.5, 143.2, 140.7, 134.9, 132.9, 132.8, 131.7, 131.2, 130.9, 130.4, 128.8, 128.4, 128.1, 128.0, 127.1, 126.5, 124.4, 121.9, 119.3, 110.8, 108.1.
**LCMS (ESI)**: *m/z* = 467.1 $[M+H]^+$ (calcd for $C_{25}H_{16}ClN_6O_2$: 467.09).

### Synthesis of AVI-4692

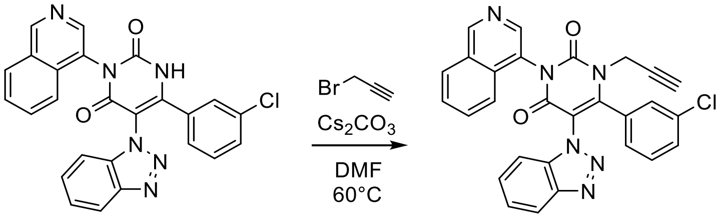


**AVI-4303** **AVI-4692**

General Procedure D (Alkylation of Uracil) was followed using AVI-4303 (20 mg, 0.0428 mmol). Yield: 10.5 mg, 0.0208 mmol, 49%.
**General**: $C_{28}H_{17}ClN_6O_2$; MW = 504.93.
**$^1$H-NMR** (400 MHz, $CD_3CN$): δ (ppm): 9.40 (s, 1H); 8.63 (d, *J* = 18.7 Hz, 1H); 8.22 (d, *J* = 8.3 Hz, 1H); 8.17-8.00 (m, 1H); 7.92 (q, *J* = 8.5 Hz, 2H); 7.82-7.52 (m, 4H); 7.36 (t, *J* = 7.6 Hz, 3H); 7.50-7.17 (m, 1H); 4.67-4.26 (m, 2H); 2.68 (m, 1H).
**$^{13}$C-NMR** (101 MHz, $CD_3CN$): δ (ppm): 160.5, 155.9, 154.9, 151.6, 145.8, 143.8, 135.6, 134.9, 133.7, 132.9, 131.7, 131.4, 131.0, 130.1, 129.7, 129.5, 129.3, 129.2, 128.4, 128.1, 126.7, 125.3, 122.0, 120.4, 111.2, 78.6, 74.6, 38.6.
**LCMS (ESI)**: *m/z* = 505.1 $[M+H]^+$ (calcd for $C_{28}H_{18}ClN_6O_2$: 505.11).

## Synthesis of AVI-4673

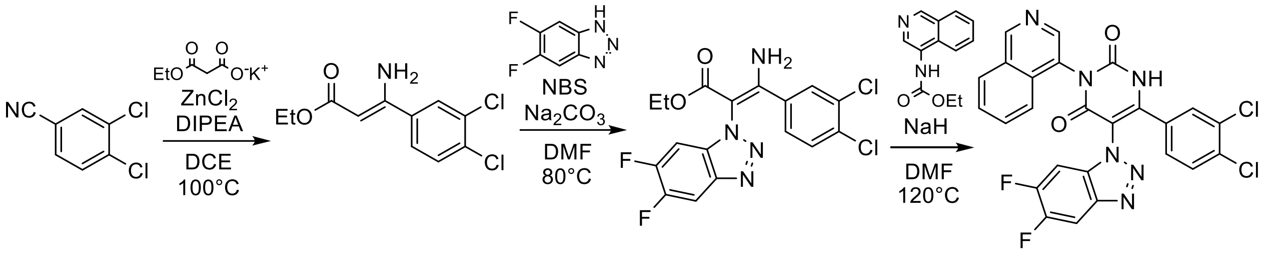


**AVI-4673**

## Step 1: Ethyl 3-amino-3-(3,4-dichlorophenyl)-2-propenoate

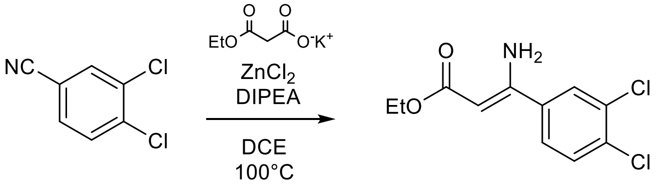


The general procedure A (Blaise Reaction) was followed, using 3,4-dichlorobenzonitrile (1 g, 5.81 mmol).
Yield: 1.42 g, 5.50 mmol, 95%.
**General**: $C_{11}H_{11}Cl_2NO_2$; MW = 260.11.
**LCMS (ESI)**: *m/z* = 260.1 $[M+H]^+$ (calcd for $C_{11}H_{12}Cl_2NO_2$: 260.02).

## Step 2: Ethyl 3-amino-2-(5,6-difluoro-1H-benzo[d][1,2,3]triazol-1-yl)-3-(3,4-dichlorophenyl)acrylate

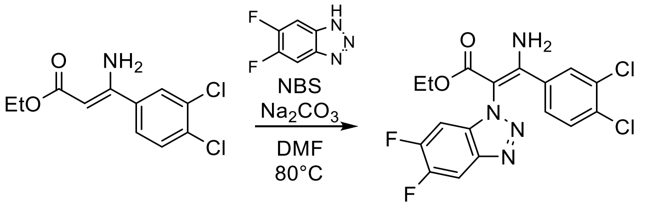


General Procedure B (Amination of Enaminone) was followed using ethyl 3-amino-3-(3,4-dichlorophenyl)-2-propenoate (80 mg, 0.309 mmol) and 5,6-difluoro-1*H*-benzotriazole. Yield: 59 mg, 0.143 mmol, 46%.
**General**: $C_{17}H_{12}Cl_2F_2N_4O_2$; MW = 413.21.
**LCMS (ESI)**: *m/z* = 413.1 $[M+H]^+$ (calcd for $C_{17}H_{13}Cl_2F_2N_4O_2$: 413.03).

## Step 3: AVI-4673

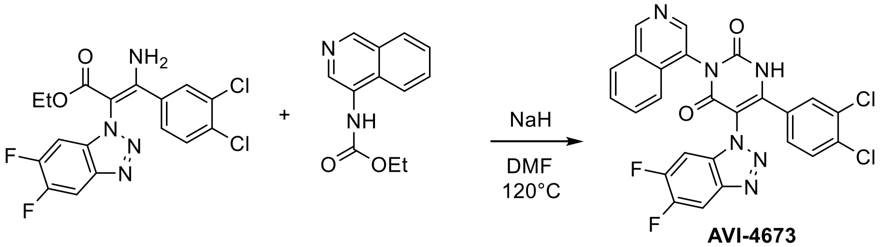


**AVI-4673**

General Procedure C (Synthesis of Uracil) was followed using ethyl 3-amino-2-(5,6-difluoro-1H-benzo[d][1,2,3]triazol-1-yl)-3-(3,4-dichlorophenyl) acrylate (59 mg, 0.143 mmol). Purification by preparative HPLC (30% to 70% $CH_3CN$ in $H_2O$ + 0.1% formic acid) afforded **AVI-4673** (29 mg, 0.0540 mmol, 38%) as a white solid.
**General**: $C_{25}H_{12}Cl_2F_2N_6O_2$; MW = 537.31.

**$^1$H-NMR** (400 MHz, DMSO-d6): δ (ppm): 12.68 (brs, 1H); 9.45 (s, 1H); 8.63 (s, 1H); 8.32-8.06 (m, 4H); 7.93 (brs, 1H); 7.83-7.73 (m, 2H); 7.61 (d, *J* = 8.3 Hz, 1H); 7.24 (brs, 1H).
**$^{13}$C-NMR** (101 MHz, DMSO-d6): δ (ppm): 160.0, 153.4, 150.6, 149.9, 147.1, 147.0, 143.1, 139.7, 139.6, 133.8, 132.7, 131.7, 131.3, 131.1, 130.9, 130.2, 130.1, 128.8, 128.1, 128.0, 127.1, 121.8, 107.7, 106.8, 106.6.
**LCMS (ESI)**: *m/z* = 537.1 $[M+H]^+$ (calcd for $C_{25}H_{13}Cl_2F_2N_6O_2$: 537.04).

**Synthesis of AVI-4694**

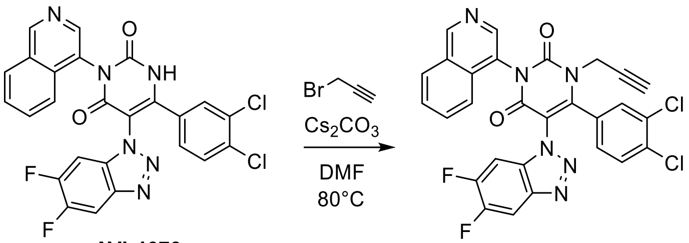


**AVI-4673** **AVI-4694**

General Procedure D (Alkylation of Uracil) was followed using AVI-4673 (200 mg, 0.372 mmol). Yield: 83.5 mg, 0.145 mmol, 39%.
**General**: $C_{28}H_{14}Cl_2F_2N_6O_2$; MW = 575.36.
**$^1$H-NMR** (400 MHz, DMSO-d6): δ (ppm): 9.48 (s, 1H); 8.67 (m, 1H); 8.34-8.08 (m, 4H); 8.03-7.76 (m, 3H); 7.75-7.57 (m, 1H); 7.55-7.29 (m, 1H); 4.73-4.18 (m, 2H); 3.51 (m, 1H).
**$^{13}$C-NMR** (101 MHz, DMSO-d6): δ (ppm): 158.7, 154.0, 153.7, 150.2, 150.1, 149.9, 149.7, 143.0, 142.9, 139.5, 139.4, 133.7, 132.4, 131.8, 131.1, 129.6, 128.8, 128.2, 127.1, 127.0, 121.7, 121.5, 106.7, 98.8, 98.6, 78.0, 76.0, 37.6.
**LCMS (ESI)**: *m/z* = 575.0 $[M+H]^+$ (calcd for $C_{28}H_{15}Cl_2F_2N_6O_2$: 575.05).

**Synthesis of AVI-4516**

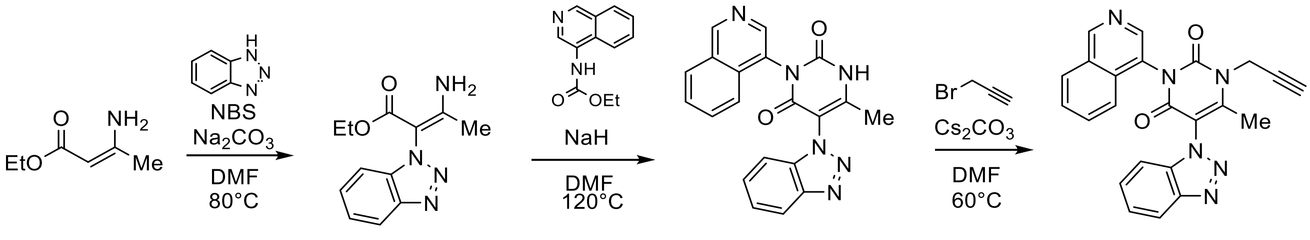


**AVI-4516**

**Step 1: Ethyl 3-amino-2-(1H-benzo[d][1,2,3]triazol-1-yl)-but-2-enoate**

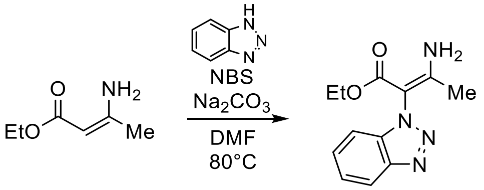


General Procedure B (Amination of Enaminone) was followed using ethyl 3-amino-but-2-enoate (50 mg, 1.56 mmol) and 1*H*-benzotriazole. Yield: 63 mg, 0.39 mmol, 66%.
**General**: $C_{12}H_{14}N_4O_2$; MW = 246.11.
**LCMS (ESI)**: *m/z* = 247.2 $[M+H]^+$ (calcd for $C_{12}H_{15}N_4O_2$: 247.11).
**Step 2: AVI-4375**

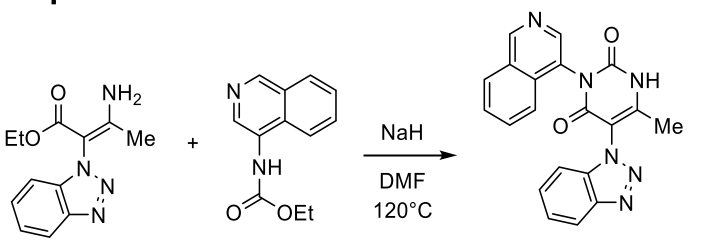


**AVI-4375**

General Procedure C (Synthesis of Uracil) was followed using ethyl 3-amino-2-(1H-benzo[d][1,2,3]triazol-1-yl)-but-2-enoate (112 mg, 0.447 mmol). Purification by preparative HPLC (10% to 100% $CH_3CN$ in $H_2O$ + 0.1% formic acid) afforded **AVI-4375** (30.2 mg, 0.082 mmol, 18%) as a white solid.
**General**: $C_{20}H_{14}N_6O_2$; MW = 370.11.
**$^1$H-NMR** (400 MHz, $CD_3OD$): δ (ppm): 12.33 (brs, 1H); 9.42 (s, 1H); 8.61 (d, *J* = 12.0 Hz, 1H); 8.28 (d, *J* = 8.1 Hz, 1H); 8.13 (d, *J* = 8.6 Hz, 1H); 8.01 (q, *J* = 10.0 Hz, 1H); 7.90-7.76 (m, 2H); 7.62 (t, *J* = 7.6 Hz, 1H); 7.53-7.50 (q, 1H); 7.43 (t, *J* = 7.1 Hz, 1H); 7.38 (t, *J* = 7.7 Hz, 1H); 2.01 (s, 3H).
**LCMS (ESI)**: *m/z* = 371.3 $[M+H]^+$ (calcd for $C_{20}H_{15}N_6O_2$: 371.12).

### Step 3: AVI-4516

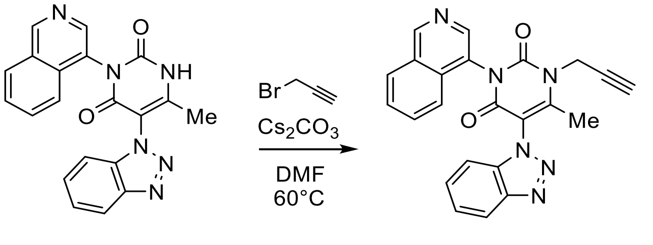


**AVI-4516**

General Procedure D (Alkylation of Uracil) was followed using AVI-4375 (20 mg, 0.054 mmol). Yield: 9.2 mg, 0.023 mmol, 42%.
**General**: $C_{23}H_{16}N_6O_2$; MW = 408.42.
**$^1$H-NMR** (400 MHz, DMSO-d6): δ (ppm): 9.49 (s, 1H); 8.71 (d, *J* = 13.6 Hz, 1H); 8.31 (d, *J* = 8.2 Hz, 1H); 8.17 (dd, *J* = 12.1, 8.5 Hz, 2H); 7.99-7.76 (m, 4H); 7.69-7.60 (m, 1H); 7.49 (td, *J* = 7.7, 2.4 Hz, 1H); 5.09-4.85 (m, 2H); 3.61 (dt, *J* = 5.6, 2.3 Hz, 1H); 2.28 (d, *J* = 7.5 Hz, 3H).
**$^{13}$C-NMR** (101 MHz, DMSO-d6): δ (ppm): 159.0, 155.4, 155.1, 153.6, 150.8, 145.3, 142.8, 134.9, 133.3, 133.1, 132.7, 128.8, 128.1, 125.0, 122.3, 122.0, 120.0, 111.6, 110.2, 78.6, 76.8, 35.9, 15.9.
**LCMS (ESI)**: *m/z* = 409.3 $[M+H]^+$ (calcd for $C_{23}H_{17}N_6O_2$: 409.13).

### Synthesis of AVI-4773

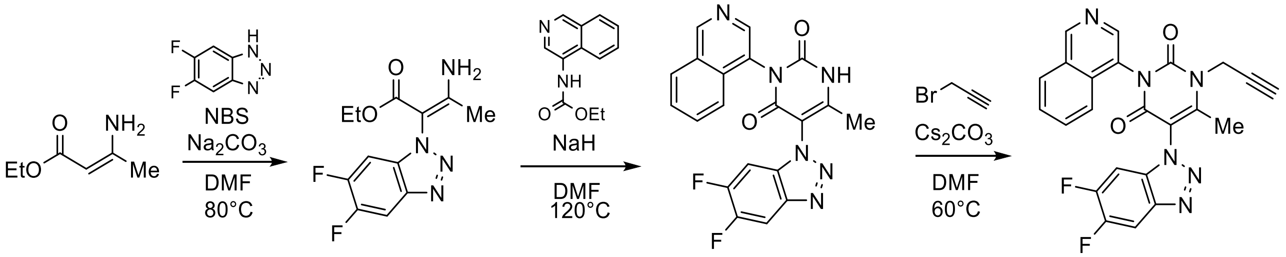


**AVI-4773**

### Step 1: Ethyl 3-amino-2-(5,6-difluoro-1H-benzo[d][1,2,3]triazol-1-yl)but-2-enoate

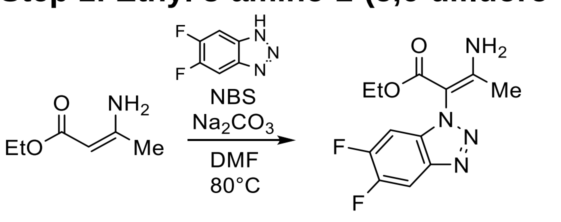


General Procedure B (Amination of Enaminone) was followed using ethyl 3-aminobut-2-enoate (83.3 mg, 0.645 mmol) and 5,6-difluoro-1*H*-benzotriazole. Yield: 137 mg, 0.485 mmol, 75%.
**General**: $C_{12}H_{12}F_2N_4O_2$; MW = 282.09.
**LCMS (ESI)**: *m/z* = 283.1 $[M+H]^+$ (calcd for $C_{12}H_{13}F_2N_4O_2$: 283.09).

**Step 2: AVI-4771**

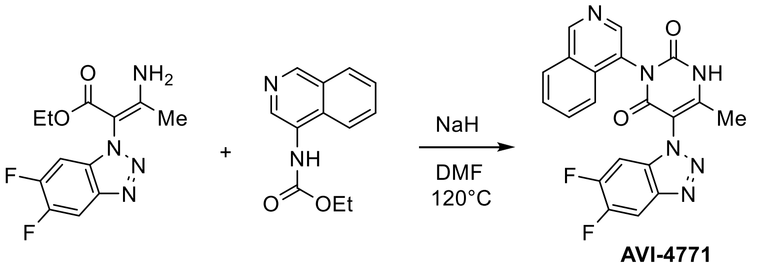


General Procedure C (Synthesis of Uracil) was followed using ethyl 3-amino-2-(5,6-difluoro-1H-benzo[d][1,2,3]triazol-1-yl)but-2-enoate (50 mg, 0.18 mmol). Purification by preparative HPLC (10% to 100% $CH_3CN$ in $H_2O$ + 0.1% formic acid) afforded **AVI-4771** (30 mg, 0.074 mmol, 42%) as a yellow solid.
**General**: $C_{20}H_{12}F_2N_6O_2$; MW = 406.10.
**$^1$H-NMR** (400 MHz, $CD_3CN$): δ (ppm): 12.35 (s, 1H); 9.43 (s, 1H); 8.61 (s, 1H); 8.35-8.31 (dd, *J* = 2.1 Hz, 1H); 8.27 (d, *J* = 8.1 Hz, 1H); 8.17 (m, 2H); 7.89 (t, *J* = 7.6 Hz, 1H); 7.78 (t, *J* = 7.7 Hz, 1H); 7.24 (dd, *J* = 8.3, 2.1 Hz, 1H); 2.03 (s, 3H).
**LCMS (ESI)**: *m/z* = 407.1 $[M+H]^+$ (calcd for $C_{20}H_{13}F_2N_6O_2$: 407.10).
**Step 3: AVI-4773**

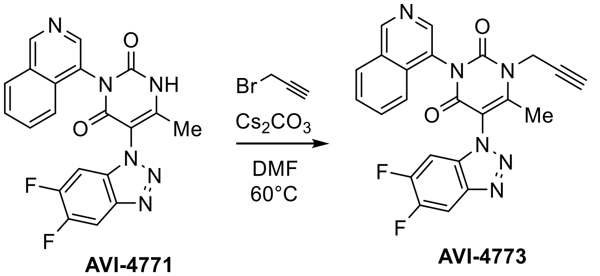


General Procedure D (Alkylation of Uracil) was followed using AVI-4771 (30 mg, 0.074 mmol). Yield: 8 mg, 0.02 mmol, 20%.
**General**: $C_{28}H_{17}ClN_6O_2$; MW = 444.11.
**$^1$H-NMR** (400 MHz, DMSO-d6): δ (ppm): 9.44 (d, *J* = 5.0 Hz, 1H); 8.66 (d, *J* = 14.8 Hz, 1H); 8.36 (ddd, *J* = 9.6, 7.1, 2.4 Hz, 1H); 8.27 (d, *J* = 8.2 Hz, 1H), 8.17 (td, *J* = 8.9, 2.8 Hz, 2H); 7.99-7.83 (m, 1H); 7.79 (m, 1H); 5.10-4.80 (m, 2H); 3.61 (dt, *J* = 6.7, 2.3 Hz, 1H); 2.30 (d, *J* = 9.7 Hz, 3H).
**$^{13}$C-NMR** (101 MHz, DMSO-d6): δ (ppm): 158.9, 155.7, 155.4, 154.0, 150.9, 143.5, 140.4, 133.0, 132.8, 132.1, 131.2, 129.1, 128.5, 127.7, 122.3, 121.9, 109.9, 107.1, 99.6, 78.5, 76.8, 35.9, 15.9.
**LCMS (ESI)**: *m/z* = 445.1 $[M+H]^+$ (calcd for $C_{28}H_{18}ClN_6O_2$: 445.11).

## Supplementary Figures

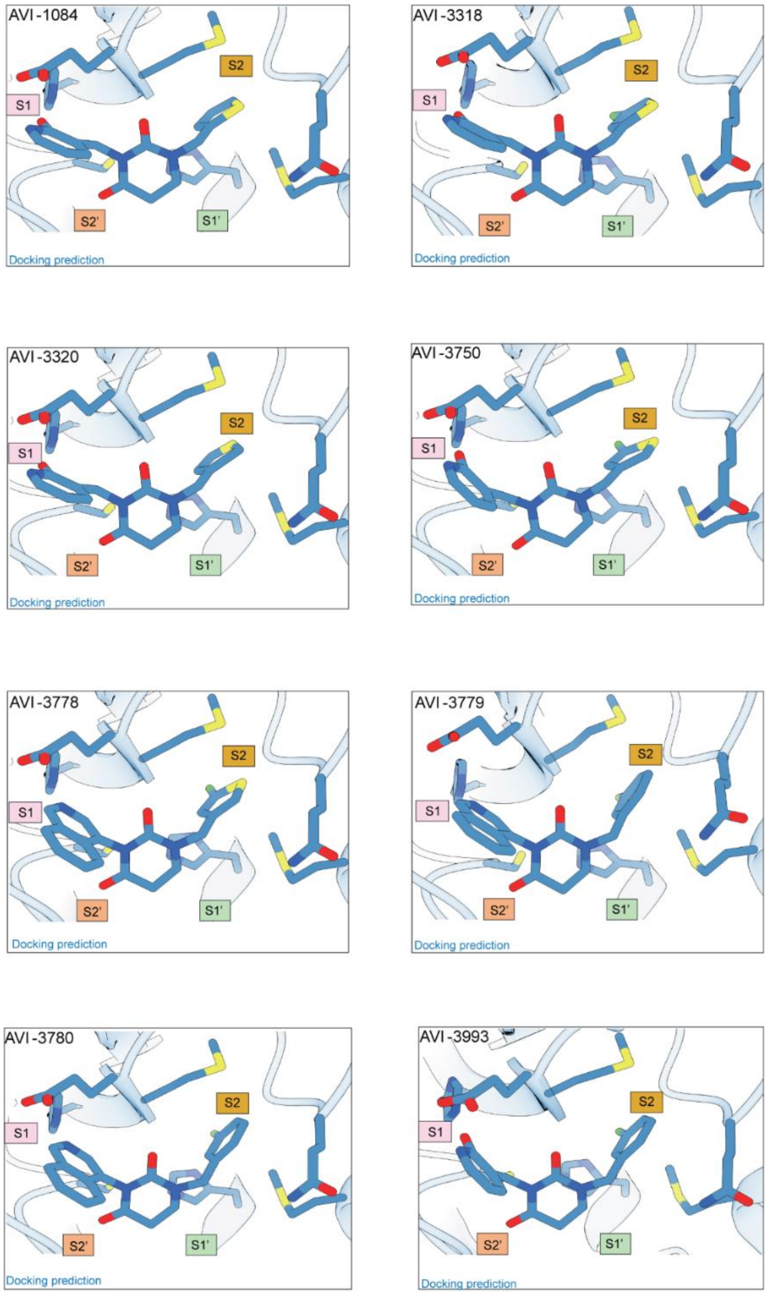


**Supplementary Fig. 1.** Docking poses for compounds in Fig. 1. The nearby $M^{Pro}$ S subsites are annotated.

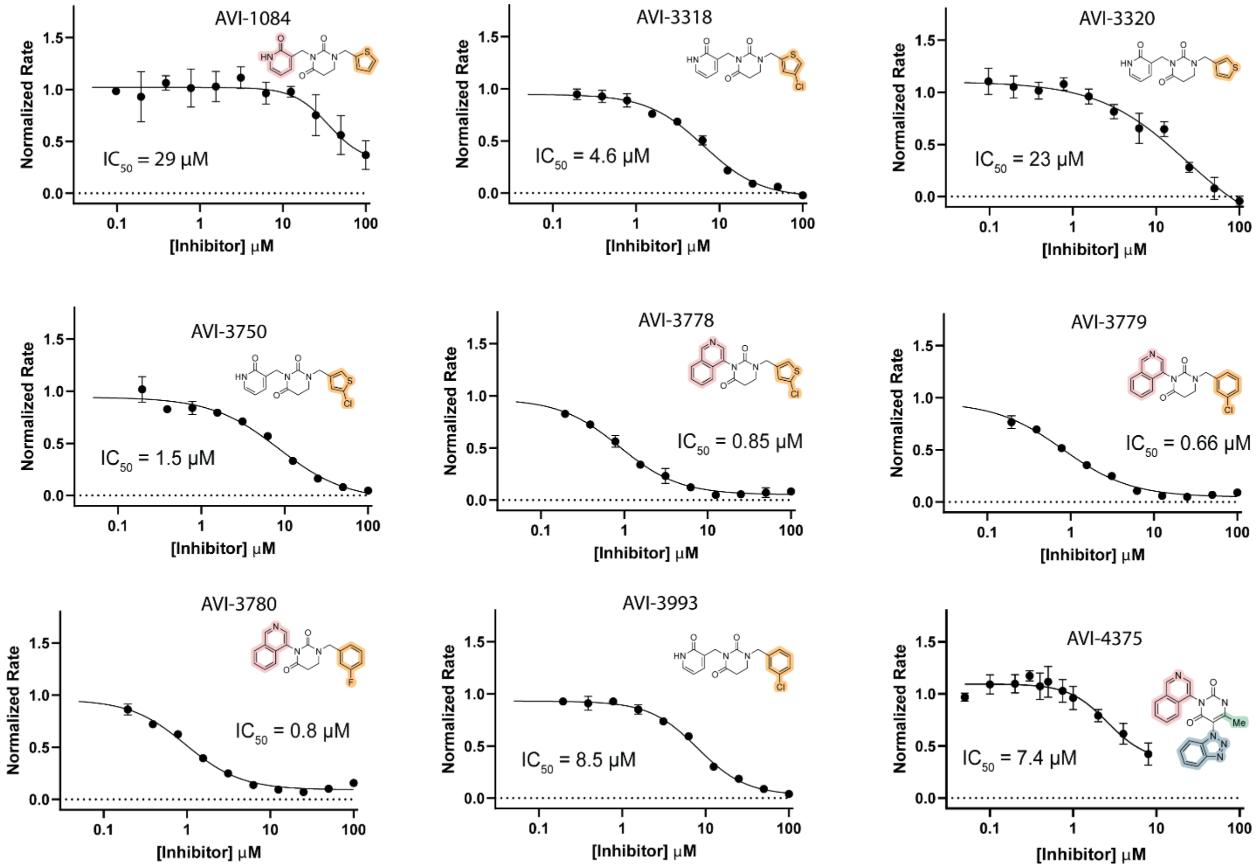


**Supplementary Fig. 2.** Dose response curves of selected compounds. (AVI-1084, AVI-3318, AVI-3320, AVI-3750, AVI-3778, AVI-3779, AVI-3780, AVI-3993, AVI-4375). Each point was performed in technical triplicate. All compounds were fit using four parameter inhibitor vs response equation in GraphPad Prism to obtain an $IC_{50}$ and error bars are plotted as ± S.D. All rates were normalized to DMSO control.

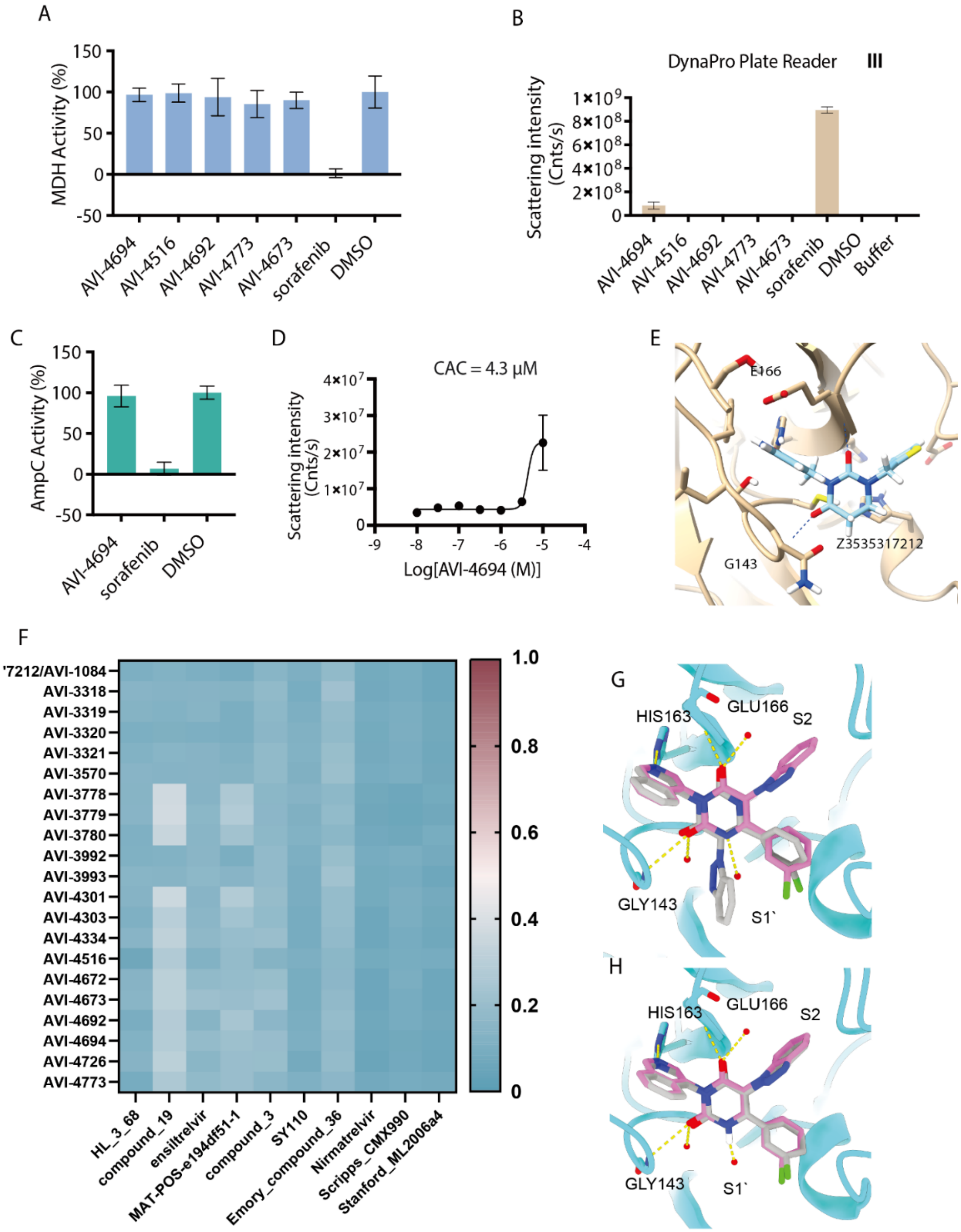


**Supplementary Fig. 3.** Aggregation testing of inhibitors. **A:** MDH activity when treated with compounds listed to test for non-specific aggregation-based inhibition. Sorafenib is used as a positive control. **B:** DLS measurement for detection of aggregation at 10 µM. **C:** AMPc activity in the presence of AVI-4694 and positive control to test for nonspecific aggregation. **D:** Dose response scattering measurement of AVI-4694 to determine critical aggregation concentration (CAC) **E:** Docking pose of Z3535317212 (AVI-1084) **F:** Comparison of Tanimoto coefficients for compounds reported here vs potent $M^{Pro}$ small molecule inhibitors compounds: HL-3-68*(1)*, compound_19*(2)*, ensitrelvir*(3)*, MAT-POS-e194df51-1*(4)*, compound_3*(5)*, SY110*(6)*, Emory_compound_36*(7)*, nirmatrelvir*(8)*, Scripps_CMX990*(9)*, Stanford_ML2026a4*(10)*. **G:** Docked pose of AVI-4303 wherein the benzotriazole binds the S1` pocket (grey, docked pose) superimposed with the complex structure of AVI-4303 (magenta). **H:** Docked pose of AVI-4303 wherein the benzotriazole bound the S2 pocket (grey, docked pose) superimposed with the complex structure of Avi-4303 (magenta).

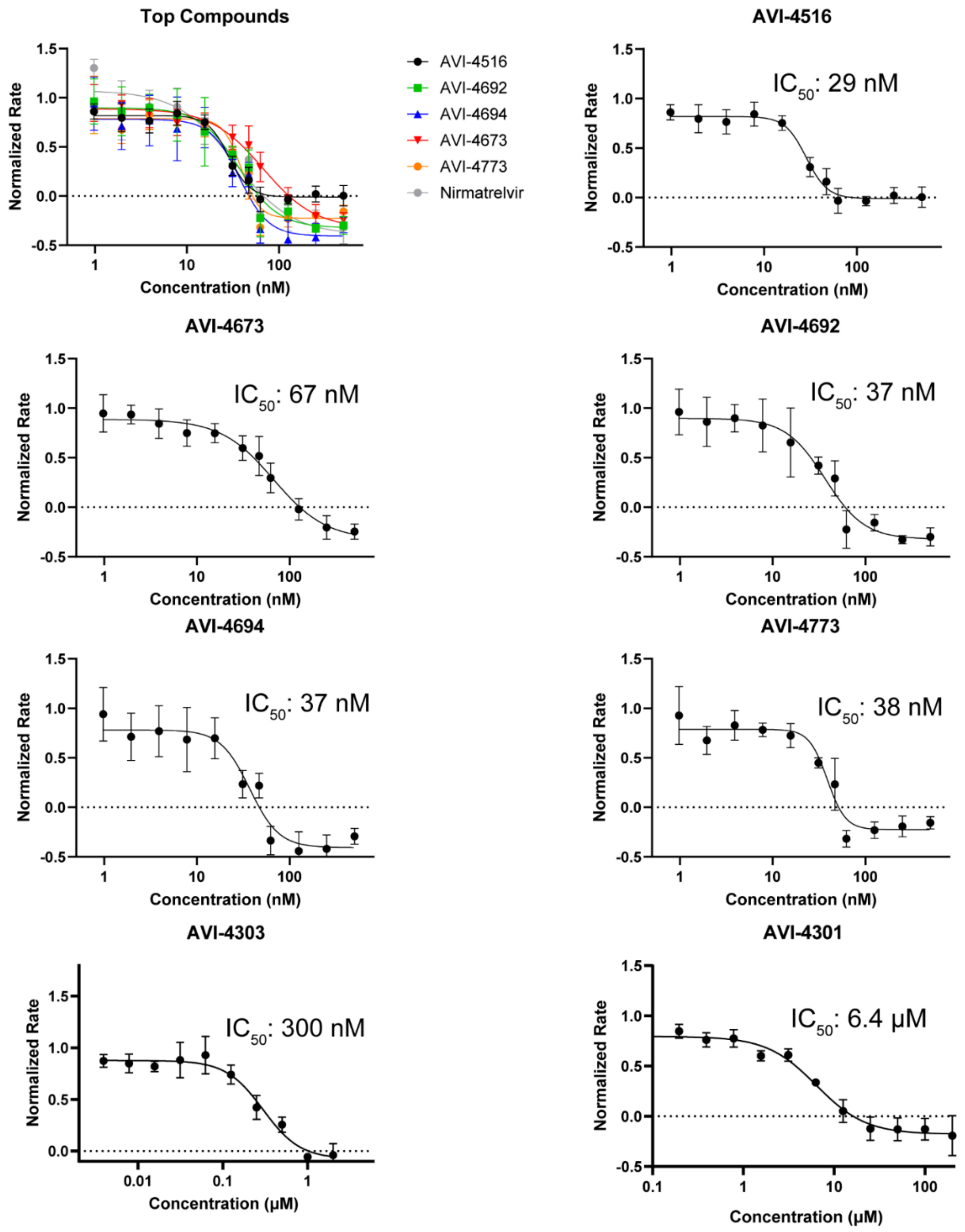


**Supplementary Fig. 4.** Biochemical assay dose response of compounds from Figure 2. Compounds were incubated with $M^{Pro}$ for 1 h and activity was then measured to generate the curve. All rates were normalized to DMSO control. Each assay was performed in technical triplicate and plotted ± S.D. and fit to a four parameter $IC_{50}$ equation using Prism.

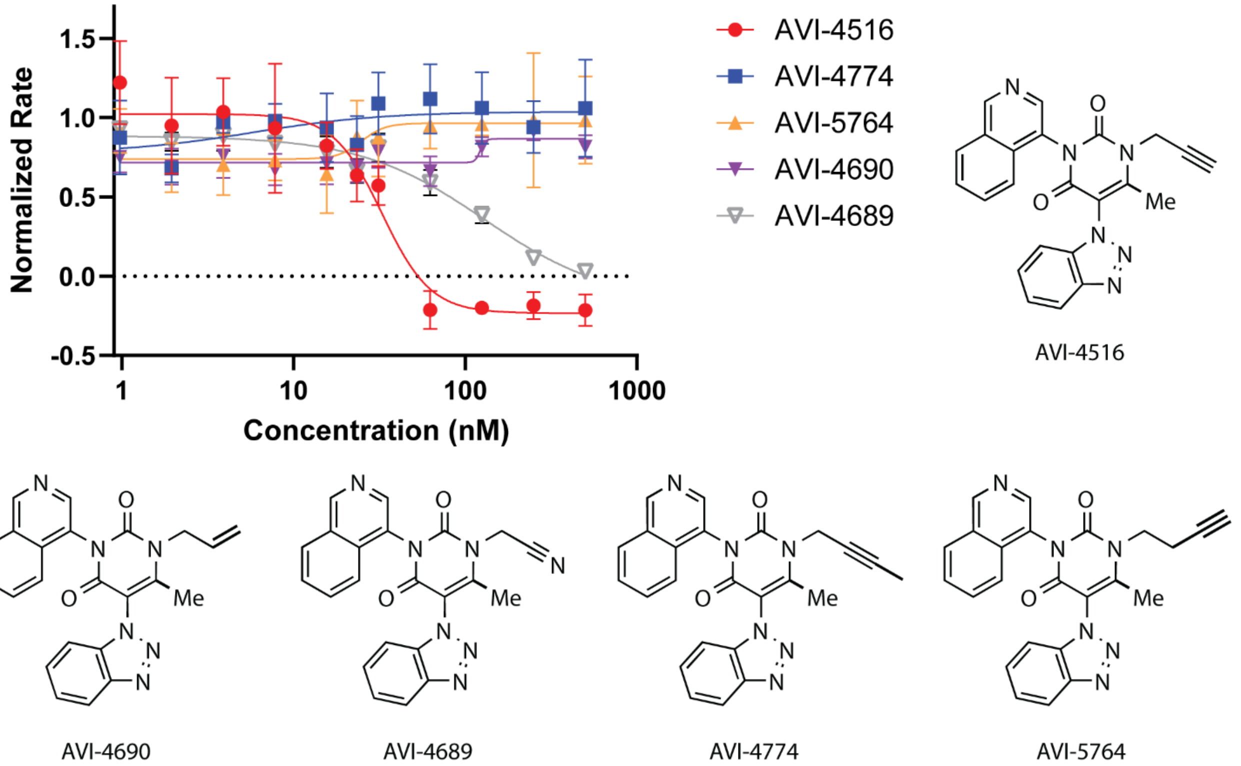


**Supplementary Fig. 5.** Biochemical inhibition in dose response for analogs of AVI-4516 bearing different N1 side chains in place of propargyl. Only nitrile analog AVI-4689 retained activity in this dose range. All rates were normalized to DMSO control. Each assay was performed in technical triplicate and plotted ± S.D. and fit to a four parameter $IC_{50}$ equation using Prism.

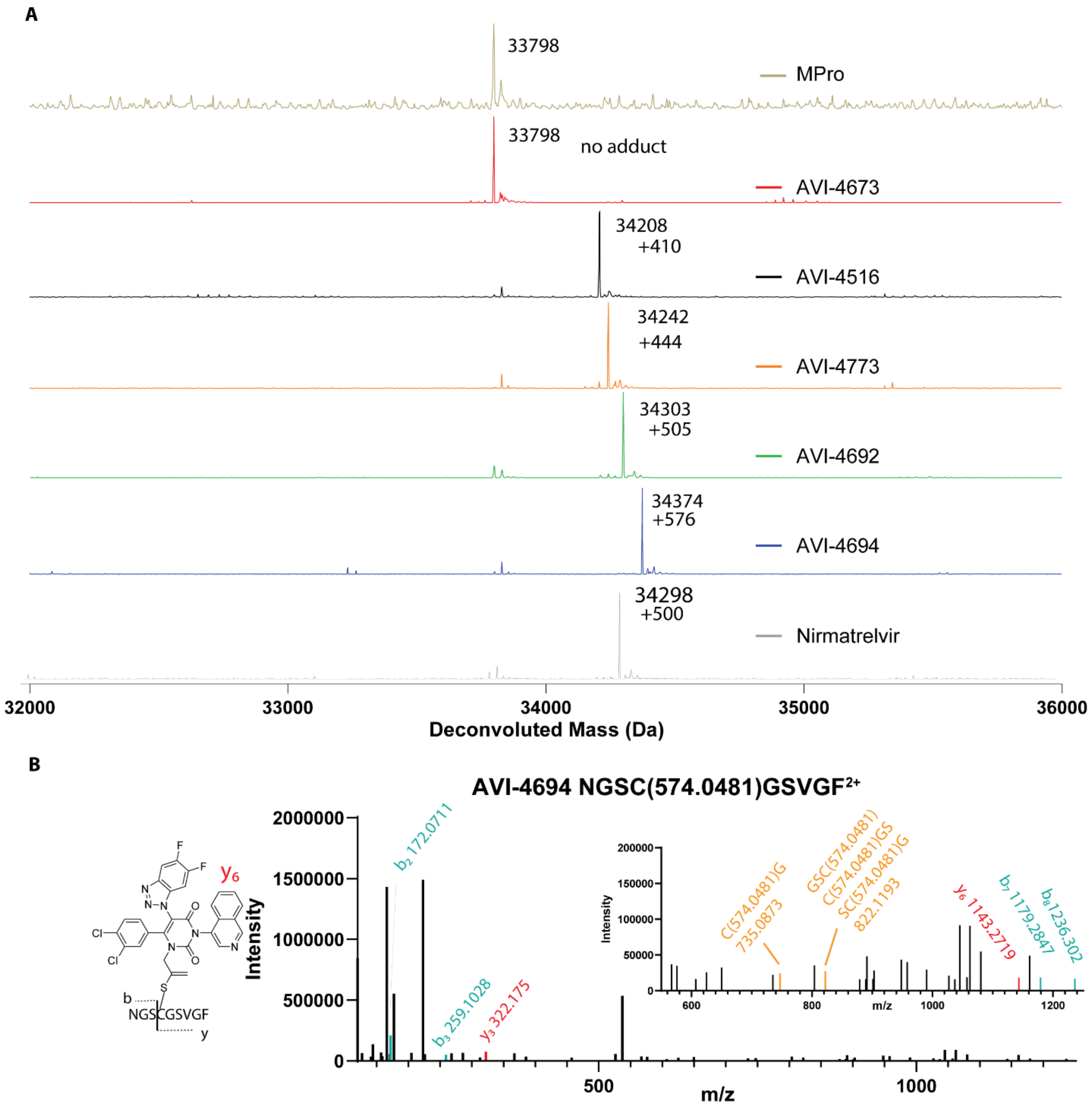


**Supplementary Fig. 6.** Mass spectrometric analysis of covalent adduct formation with MPro C145. **A:** Deconvoluted whole protein denaturing MS data for Mpro alone or upon incubation with AVI-4673, AVI-4516, AVI-4773, AVI-4692, AVI-4694, or nirmatrelvir. 10 µM of $M^{Pro}$ was treated with 100 µM of compound overnight, then diluted to 500 nM enzyme and analyzed via MS. The observed adduct after deconvolution is noted next to main peak. **B:** Structure of predicted adduct based on previous literature*(11)* and the complex structures of AVI-4692 and AVI-4516 bound to $M^{Pro.}$ At right, MS2 spectra of $M^{Pro}$ treated with 4694 and then digested with chymotrypsin. The MS1 ion that was selected was NGSC(574.0481)GSVGF$^{2+}$ $Y_6$ and $b_3$ ions are noted in the spectra. The ion that comprises C(574.0481)G shows that the modification is localized to the cysteine.

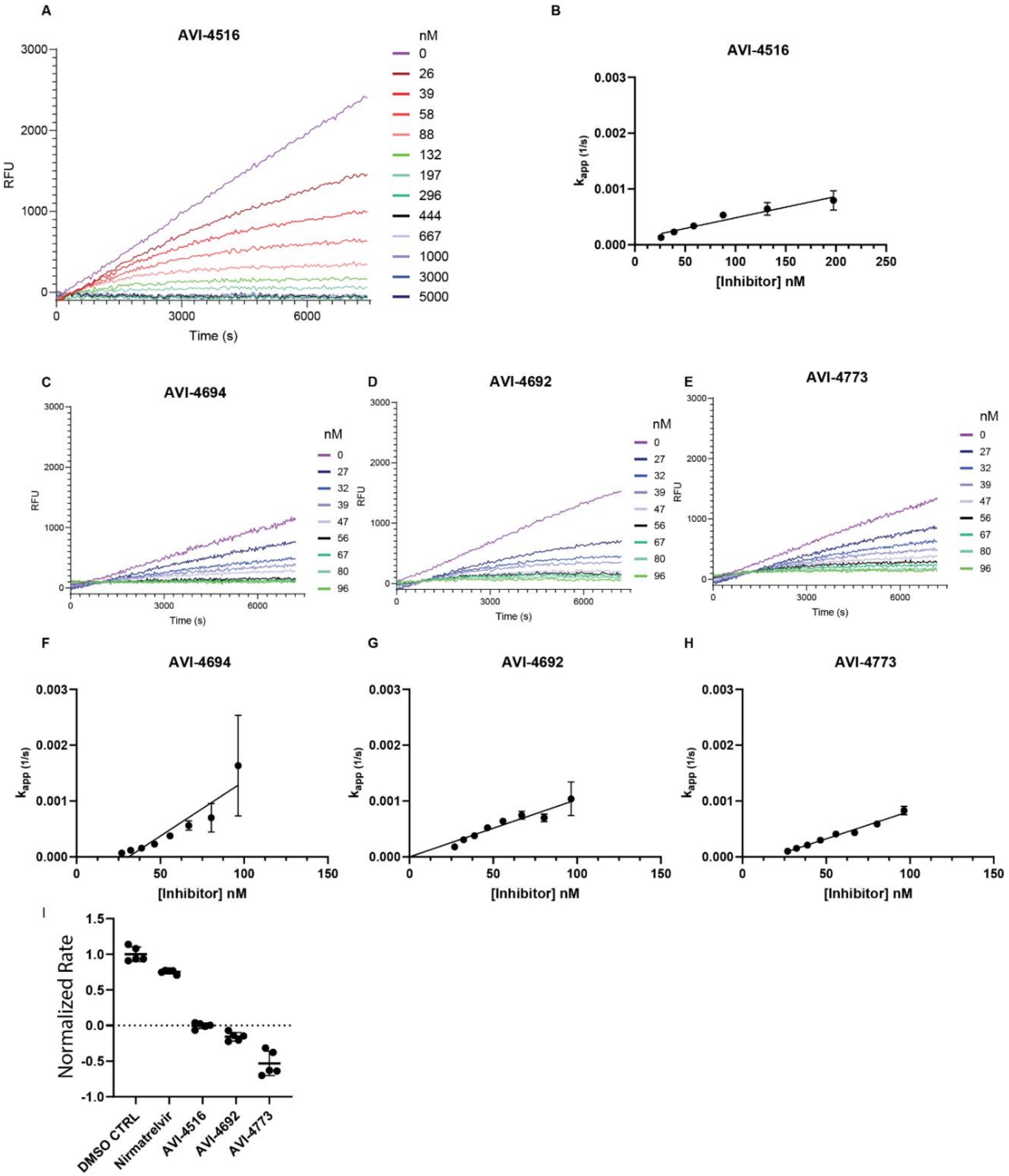


**Supplementary Fig. 7.** Inhibitor-kinetic experiments for AVI-4516, AVI-4773, AVI-4692, and AVI-4694. **A:** Average blank subtracted raw traces for AVI-4516. **B**. A plot of the linear portion of AVI-4516 $k_{app}$ vs inhibitor concentration and linear fit used to determine $k_{inact}/K_I$ to compare with the other inhibitors in the series. **C-D:** Average blank subtracted raw traces for AVI-4694, AVI-4692, and AVI-4773. **F-H:** A plot of $k_{app}$ vs inhibitor concentration and linear fit used to determine $k_{inact}/K_I$. All inhibition kinetic experiments were done in technical quintuplicate. Error bars are plotted as 95% CI. **I.** Dialysis experiment after 7 days of incubation with compound. Normalized rates when 100 µL of 1 µM $M^{Pro}$ is treated with 1.5 µM inhibitor and then dialyzed against 300 mL of assay buffer for 7 d at RT then diluted to 50 nM enzyme in kinetic assay (final 60,000 x dilution). Each rate was measured in technical quintuplicate error bars are ± S.D.

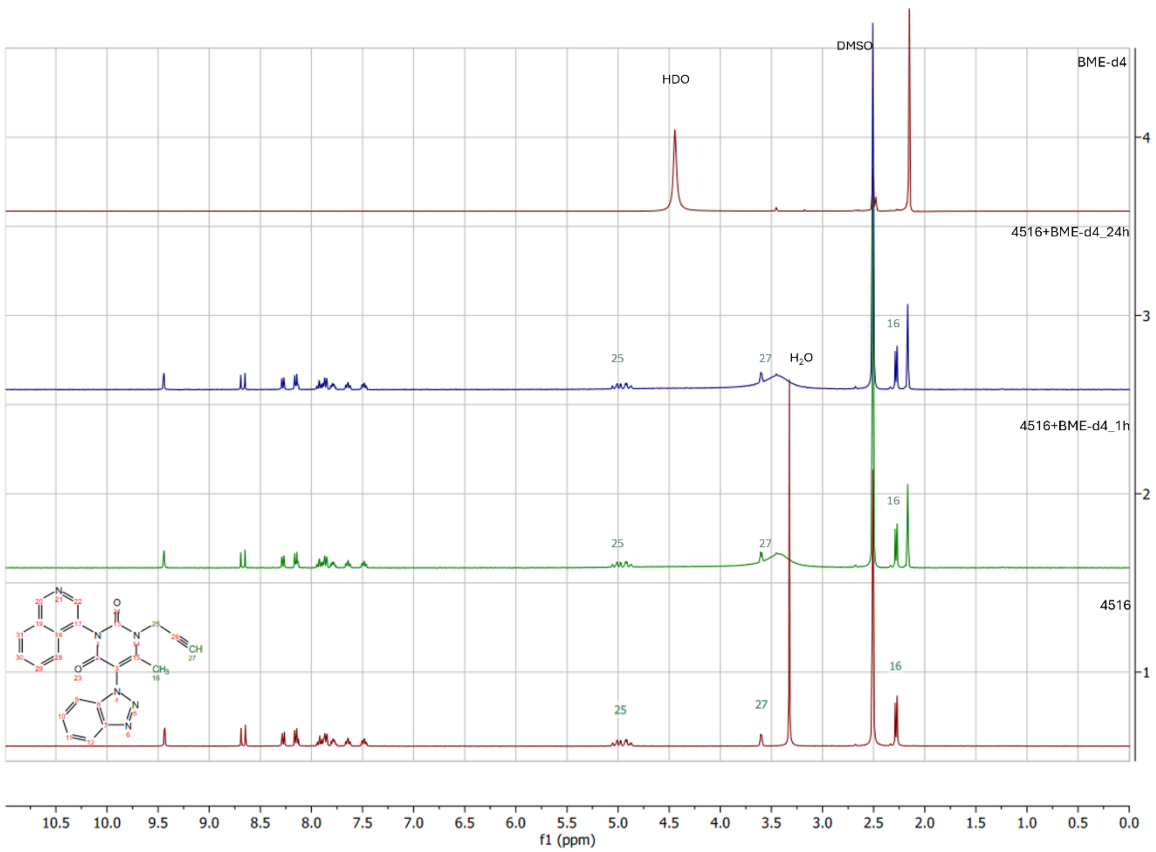


**Supplementary Fig. 8.** AVI-4516 is unreactive with a large excess (10 equiv.) thiol *d4*-betamercaptoethanol (*d4*-BME). $^{1}$H NMR spectrum of AVI-4516 alone (bottom red trace), and in the presence 10 equiv. of *d4*-BME after 1 h (green trace) and 24 h (blue trace). The spectra of *d4*-BME (top red trace) shows only resonances for residual protio solvent peaks. The spectrum of AVI-4516 is unchanged in the presence of d4-BME, indicating a lack of general thiol reactivity. Propargyl methylene (peak 25) and methine (peak 27) resonances are indicated.

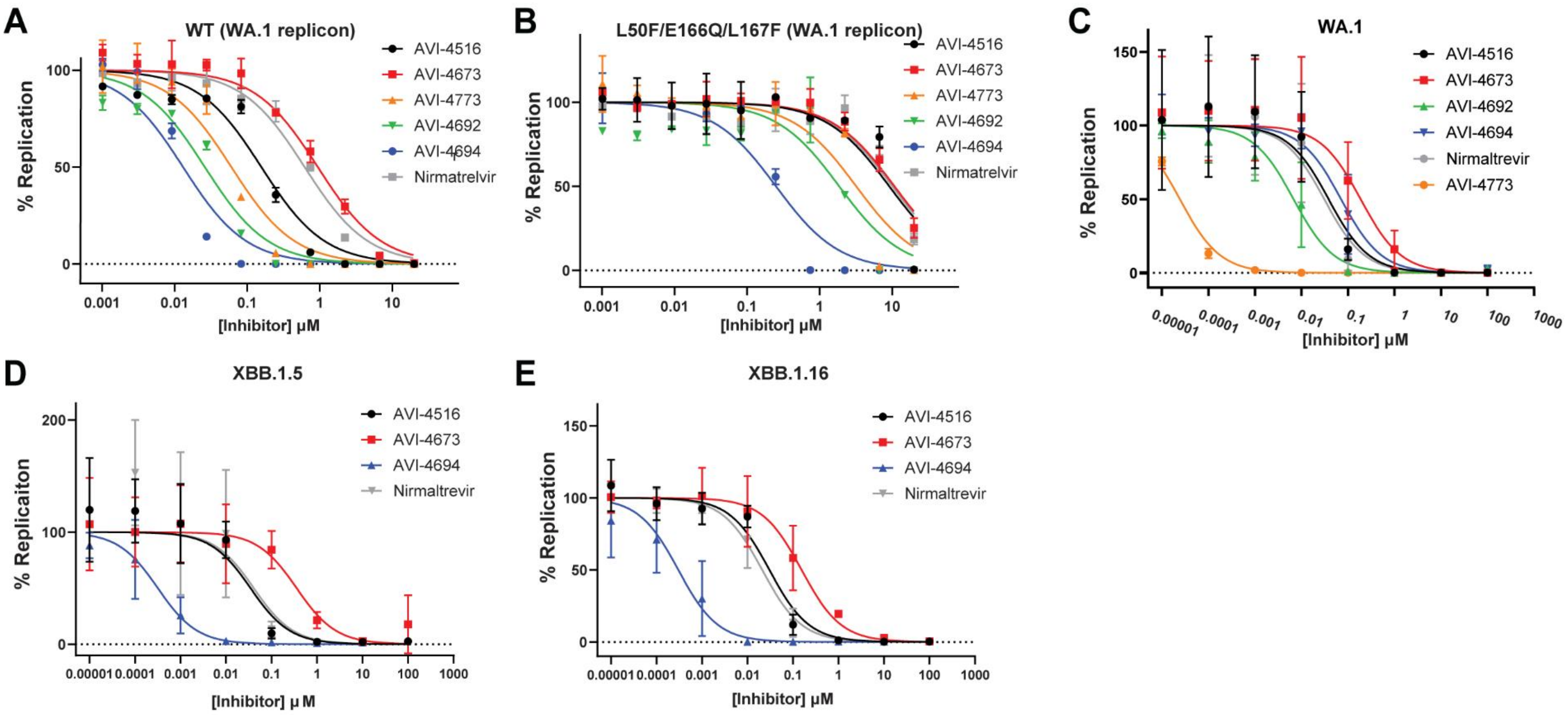


**Supplementary Fig. 9.** Dose response curves of SARS-CoV-2 replicon and viral infection. **A-B:** Replicon-based dose response curves. **C-E:** Live virus Incucyte-based measurements. Each point was measured in biological triplicate. Error bars were plot ± S.D. Inhibition curves were fit using Prism.

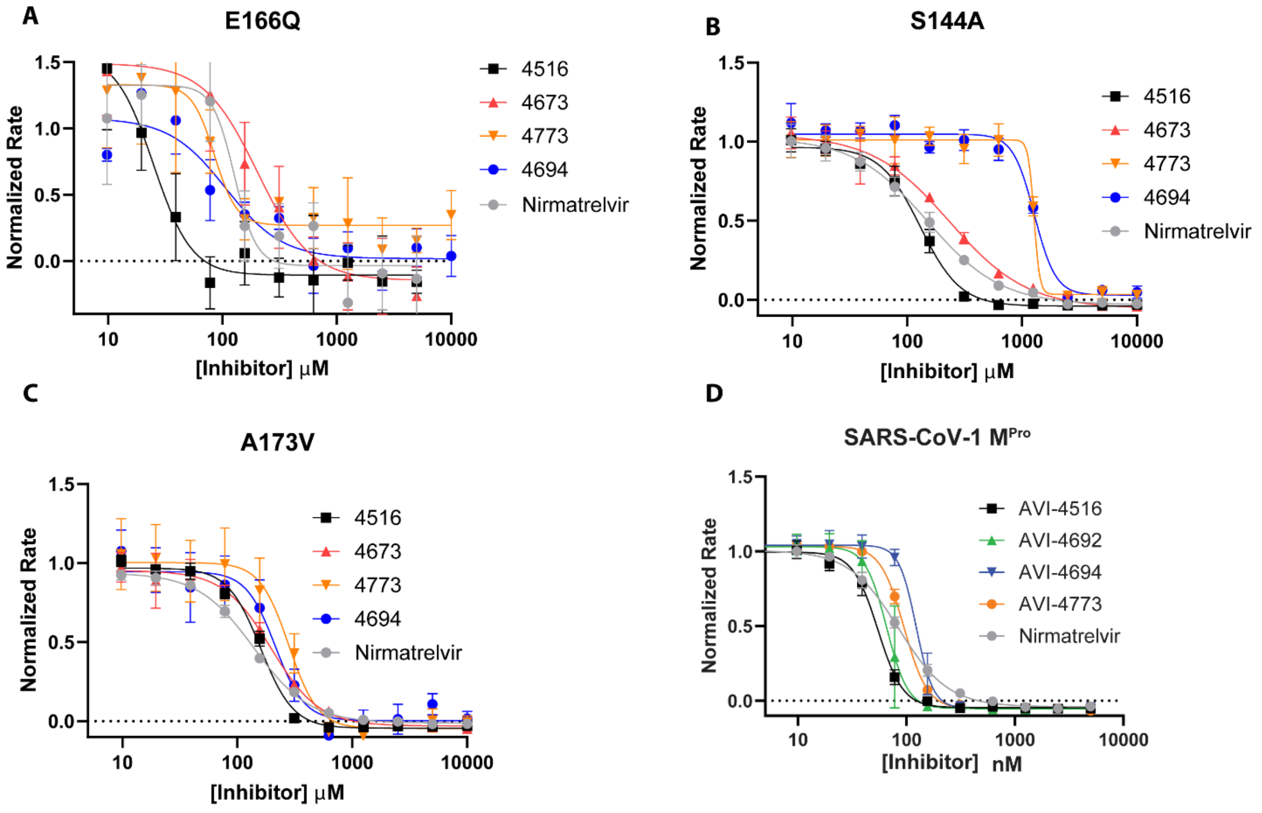


| IC50 (nM) | | | | | | |
|---|---|---|---|---|---|---|
| | **AVI-4516** | **AVI-4673** | **AVI-4773** | **AVI-4694** | **AVI-4692** | **Nirmatrelvir** |
| **A173V** | 160 | 200 | 280 | 210 | | 140 |
| **S144A** | 130 | 230 | 1200 | 1300 | | 150 |
| **E166Q** | 25 | 200 | 85 | 110 | | 130 |
| **SARS-CoV-1 $M^{Pro}$** | 55 | | 95 | 66 | 120 | 85 |

**Supplementary Fig. 10.** Dose response of AVI-4516, AVI-4673, AVI-4773, AVI-4694, and nirmatrelvir vs activity of selected nirmatrelvir resistant mutants and SARS-CoV-1 $M^{Pro}$. Each point was performed in technical triplicate. All compounds were fit using four parameter inhibitor vs response equation in Prism to obtain an $IC_{50}$. All rates were normalized to DMSO control. Error bars were plotted as ± S.D.

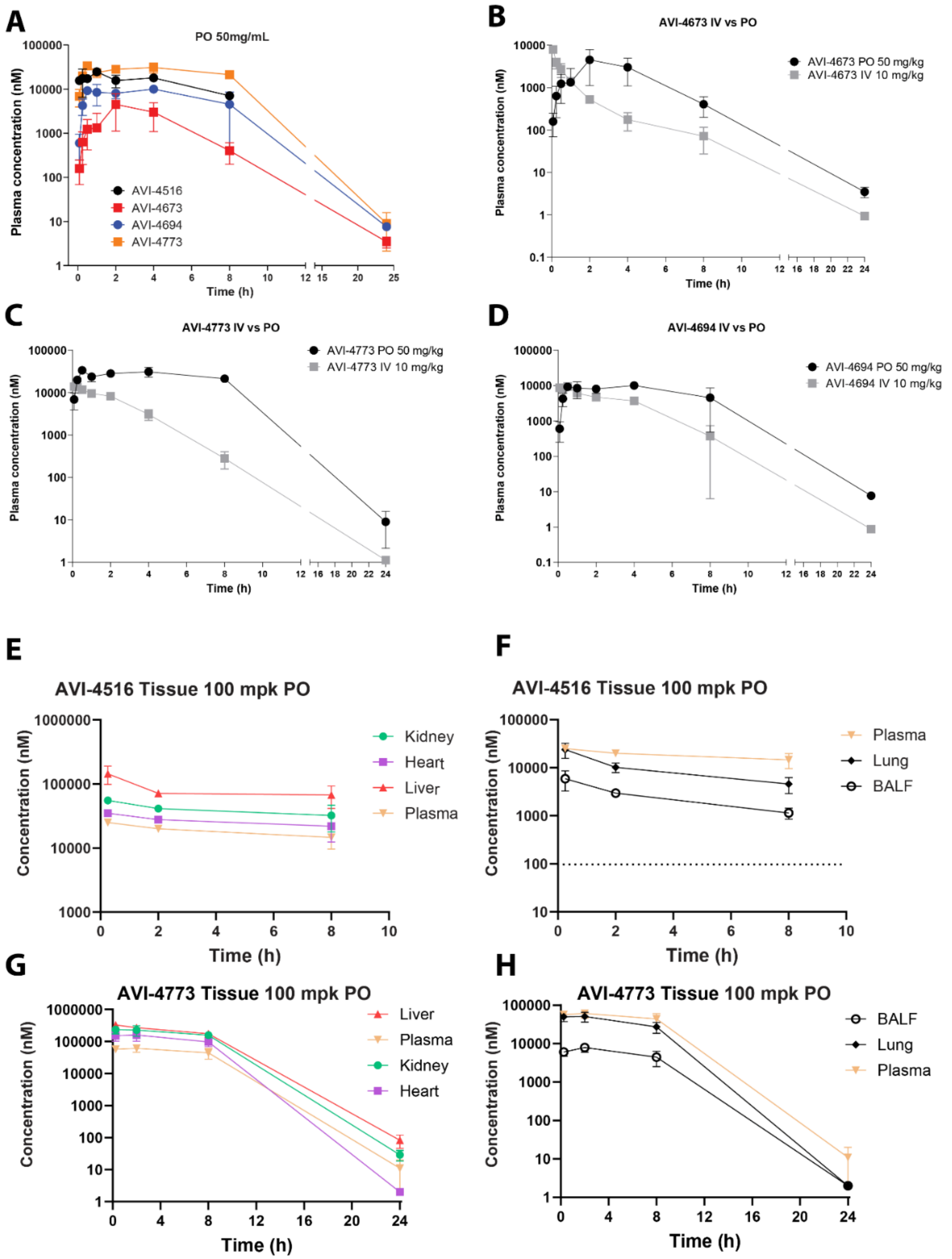


**Supplementary Fig. 11.** Mouse PK comparison of oral dosing and IV dosing. **A:** comparison of concentration in plasma through oral dosing (50mg/kg) of AVI-4516, AVI-4694, AVI-4673 and AVI-4773. **B-D:** Plasma concentration comparison of PO at 50 mg/kg and IV at 10mg/kg dosing scheme for AVI-4673, AVI-4773, and AVI-4694 respectively. **E:** Kidney, heart, and liver distribution compared to plasma of AVI-4516 after 100 mg/kg PO. Error bars are plotted as ±SD. **F:** Comparison of AVI-4516 (100 mg/kg PO) in lung, plasma and BAL fluid. Error bars are plotted as ±SD. **G**: Kidney, heart, and liver distribution compared to plasma of AVI-4773 after 100 mg/kg PO. **H**: Comparison of AVI-4773 (100 mg/kg PO) in lung, plasma and BAL fluid. Error bars are plotted as ±SD.

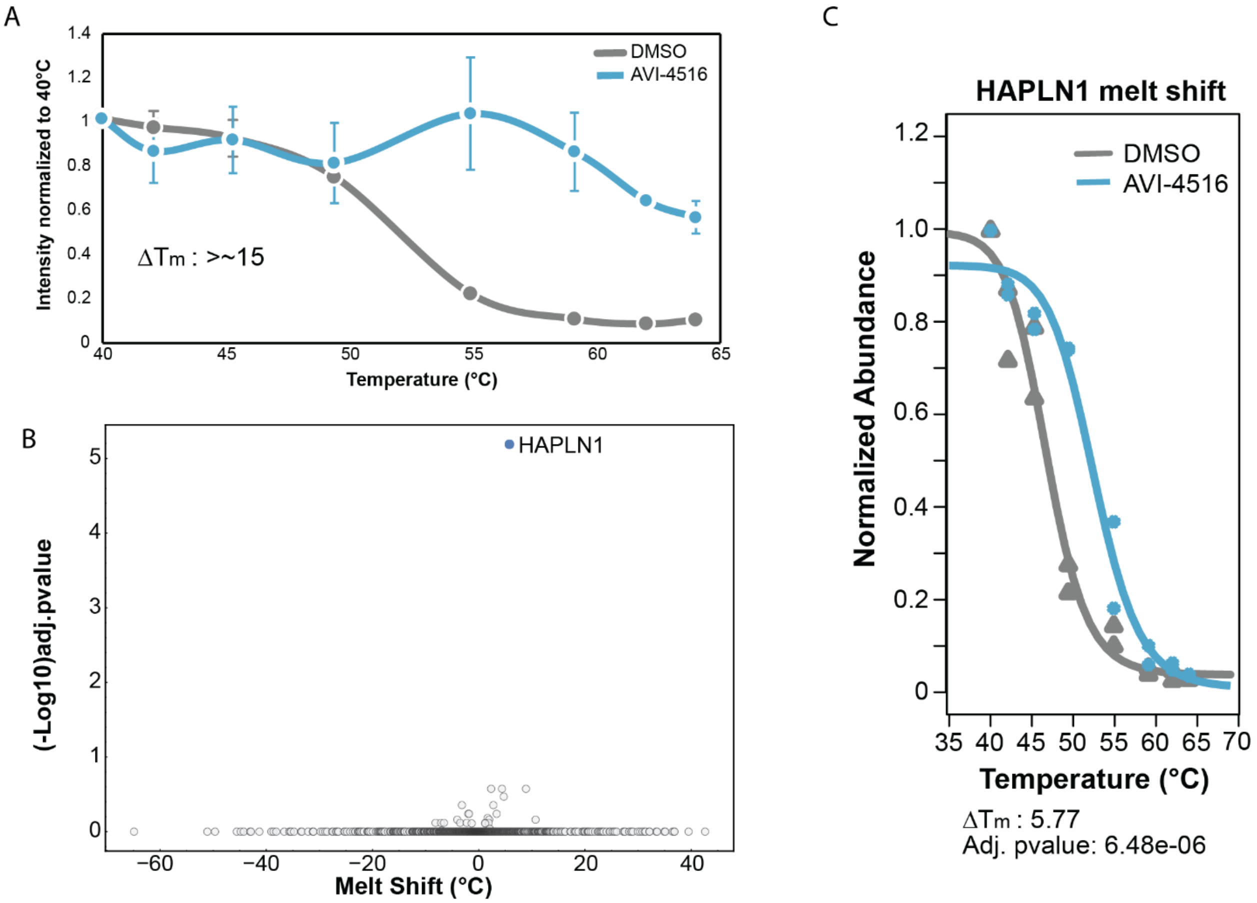


**Supplementary Fig. 12**. $M^{Pro}$ $T_m$ shift and TPP data. **A:** The $T_m$ of purified $M^{Pro}$ alone with increasing concentrations of AVI-4516, the compound stabilizes the protein >15 °C. This experiment serves as a positive control for **B**. **B:** Volcano plot of TPP analysis of A549 lysates treated with **AVI-4516**, revealed a single protein that exhibited statistically significant increase in $T_m$. C. Shift in the melting curve of HAPLN1 in AVI-4516 treated lysate vs. DMSO control. The apparent shift is 5.77 °C

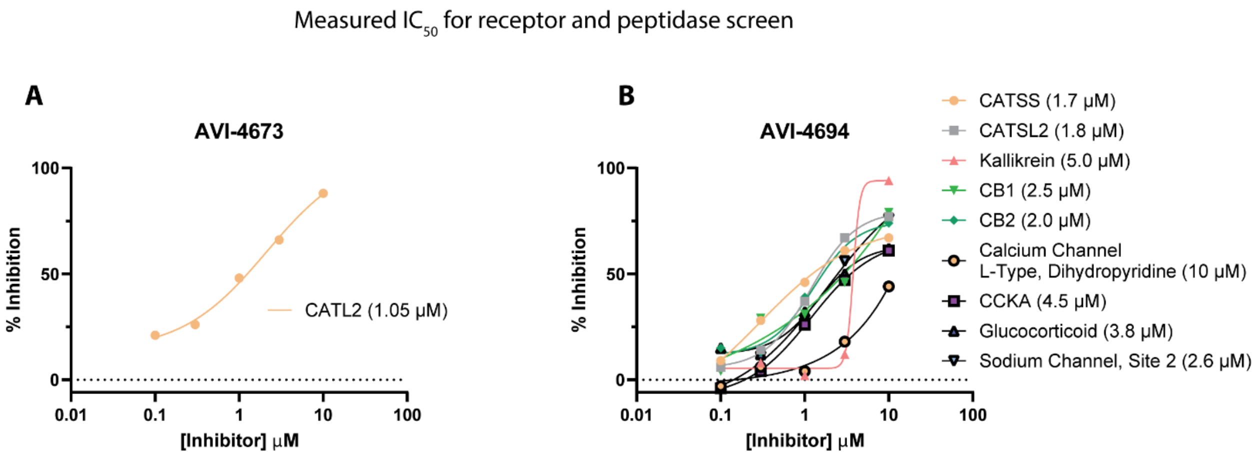


**Supplementary Fig. 13.** Measured $IC_{50}$s from i*n vitro* safety screen. **A**: AVI-4673 dose response curve for Cathepsin L. **B**: AVI-4694 dose response curve for Cathepsin L, Cathepsin S, Kallikrein, cannabinoid receptor CB1 and CB2, Calcium Channel L-Type, CCK, Glucocorticoid and Sodium channel. Computed $IC_{50}$s are noted in the legend.

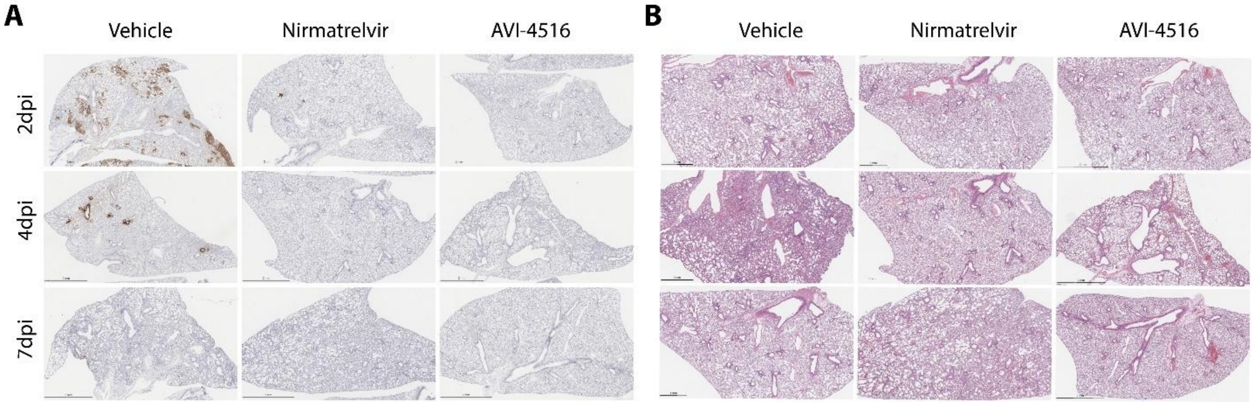


**Supplementary Fig. 14.** Representative images of immunohistochemistry of the SARS-CoV-2 N protein (**A**) and Hematoxylin and eosin (H&E) staining (**B**) in the left lung lobe of mice from different treatment groups at the specified time points. Scale bars represent 2mm.

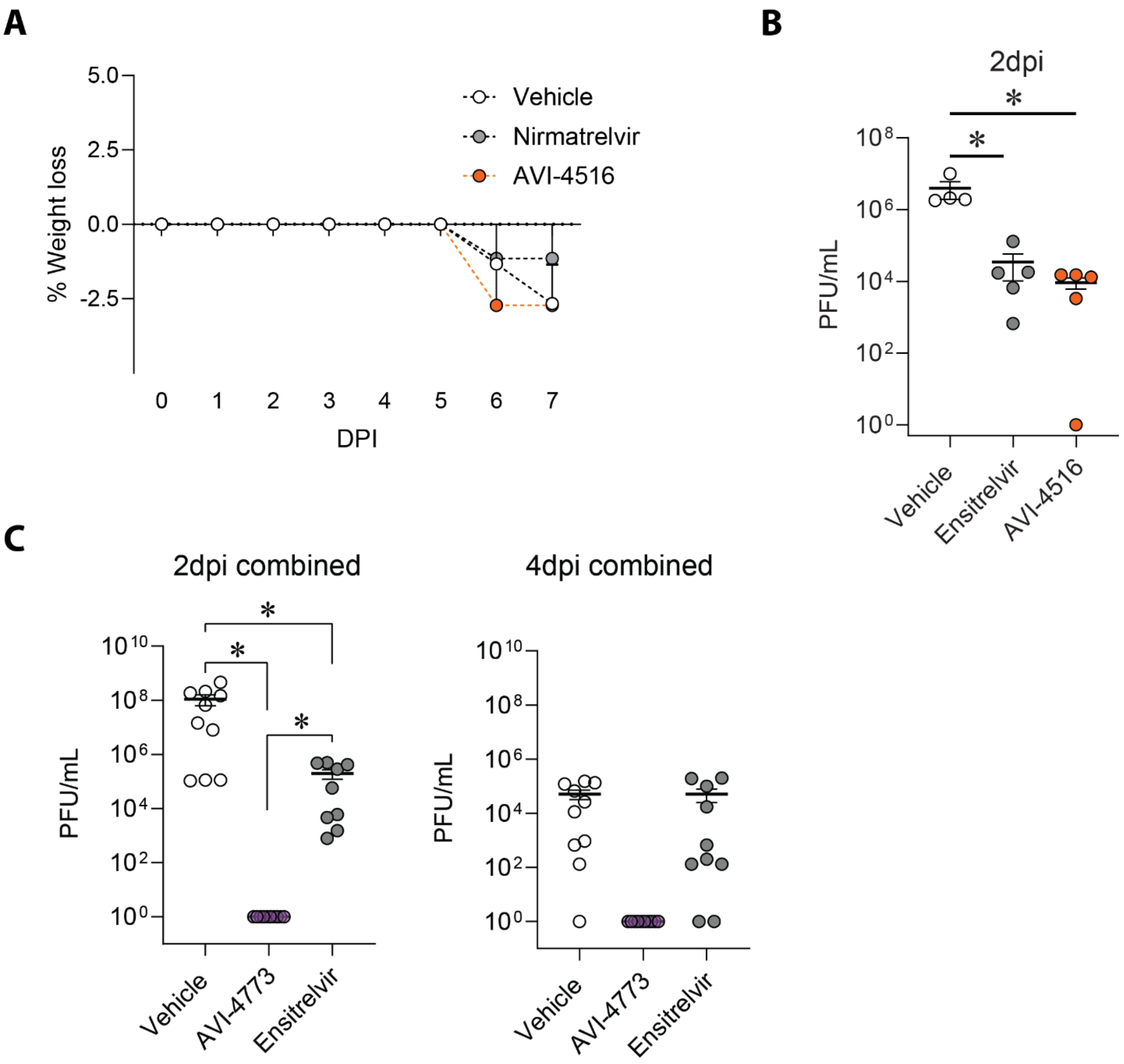


**Supplementary Fig. 15.** Efficacy of AVI-4516 and AVI-4773 in mouse infection model. **A:** Weight of mice treated with vehicle or test compounds over the course of study. The data are plotted +/- SD.**B:** Antiviral effects of ensitrelvir and AVI-4516 in SARS-CoV-2 (Beta variant) infected mice. In this study, 100 mg/kg BID of each compound was administered to WT mice after 4 hours of infection. Animals were sacrified and viral titers were measured from harvested lungs at 2 days post infection (dpi). **C:** Efficacy of AVI-4773 as compared to ensitrelvir. In this study, WT mice (n=10 per time point per treatment group) infected with SARS-CoV-2 (Beta variant) were treated with 100 mg/kg BID dosing of AVI-4773 and ensitrelvir. A subset of mice was euthanized at either 2 or 4 days post-infection, and lung tissues were harvested and homogenized for plaque assays. The graphs depict viral replication, measured as plaque-forming units, across treatment groups and time points. Data are presented as mean ± s.e.m. for each time point and analyzed using a two-tailed unpaired Student's t-test. Each data point represents the infectious virus titer in an individual mouse (*$p < 0.05$)

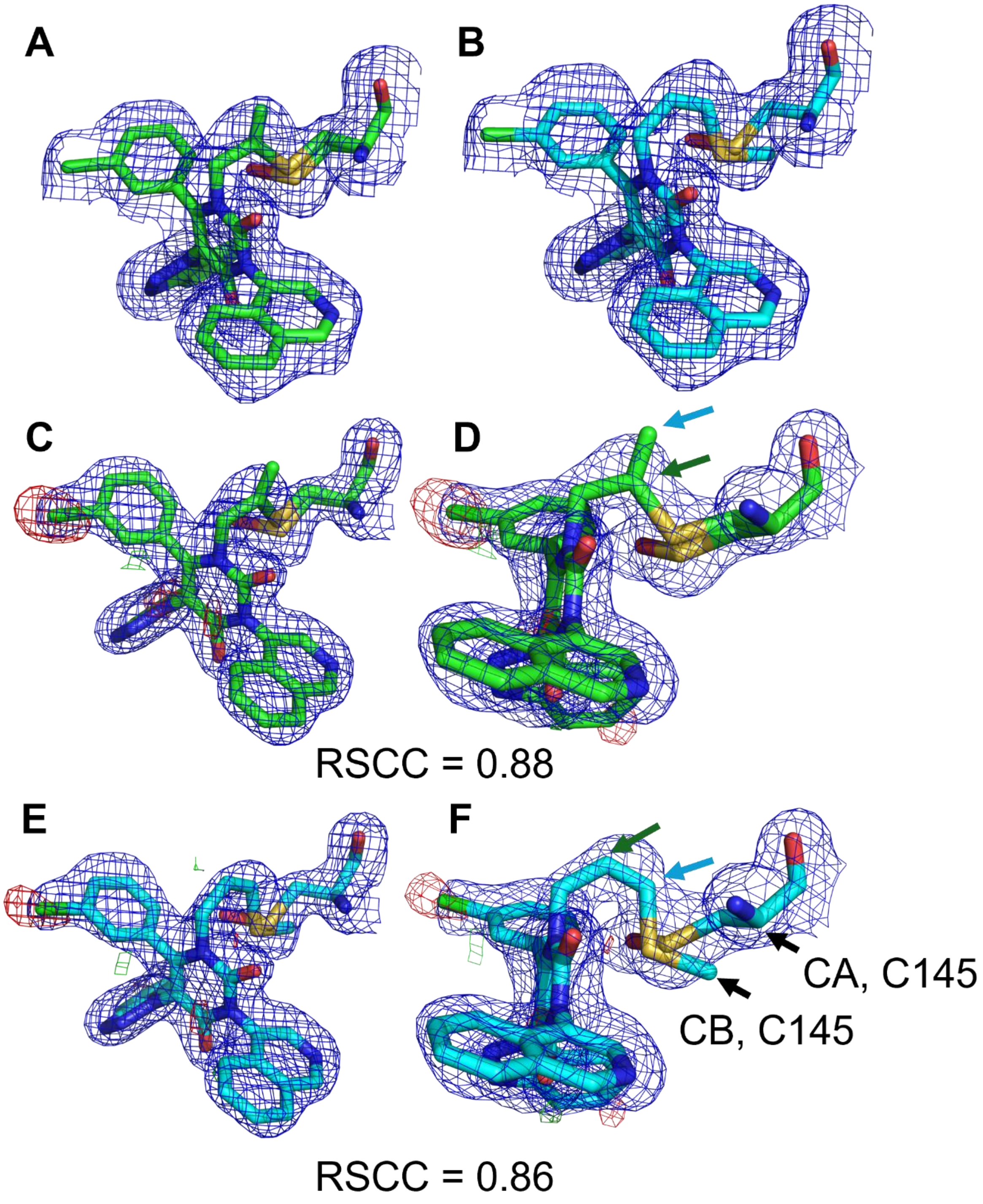


**Supplementary Fig. 16.** Comparison of density maps for cysteine 145 connectivity to **AVI-4692**. Omit maps (Fo-Fc electron density in blue contoured at 1σ) of the bound ligand AVI-4692 reacting either at its internal carbon **(A)** or terminal carbon **(B)**. Visualization of the ligand AVI-4692 reacting either at its internal carbon **(C,D)** or terminal carbon **(E,F)** fit into the 2mFo-DFc map in blue contoured at 1σ and mFo-DFc map at 3σ in red (negative) and green (positive). The internal and terminal carbon are shown with a green or a blue arrow on the side views **(D,F)**. Partial occupancy oxidized cysteine 145 is also shown. In the reaction with the terminal carbon, accommodating the compound within the electron density places $C_\alpha$ of the cysteine 145 at 2.33 Å distance from $C_\beta$ breaking the bond distance for covalent bond. This further supports the reaction mechanism through the internal carbon. Internally reacted AVI-4692 shows better fit evidenced by higher RSCC value obtained with wwPDB Validation System.

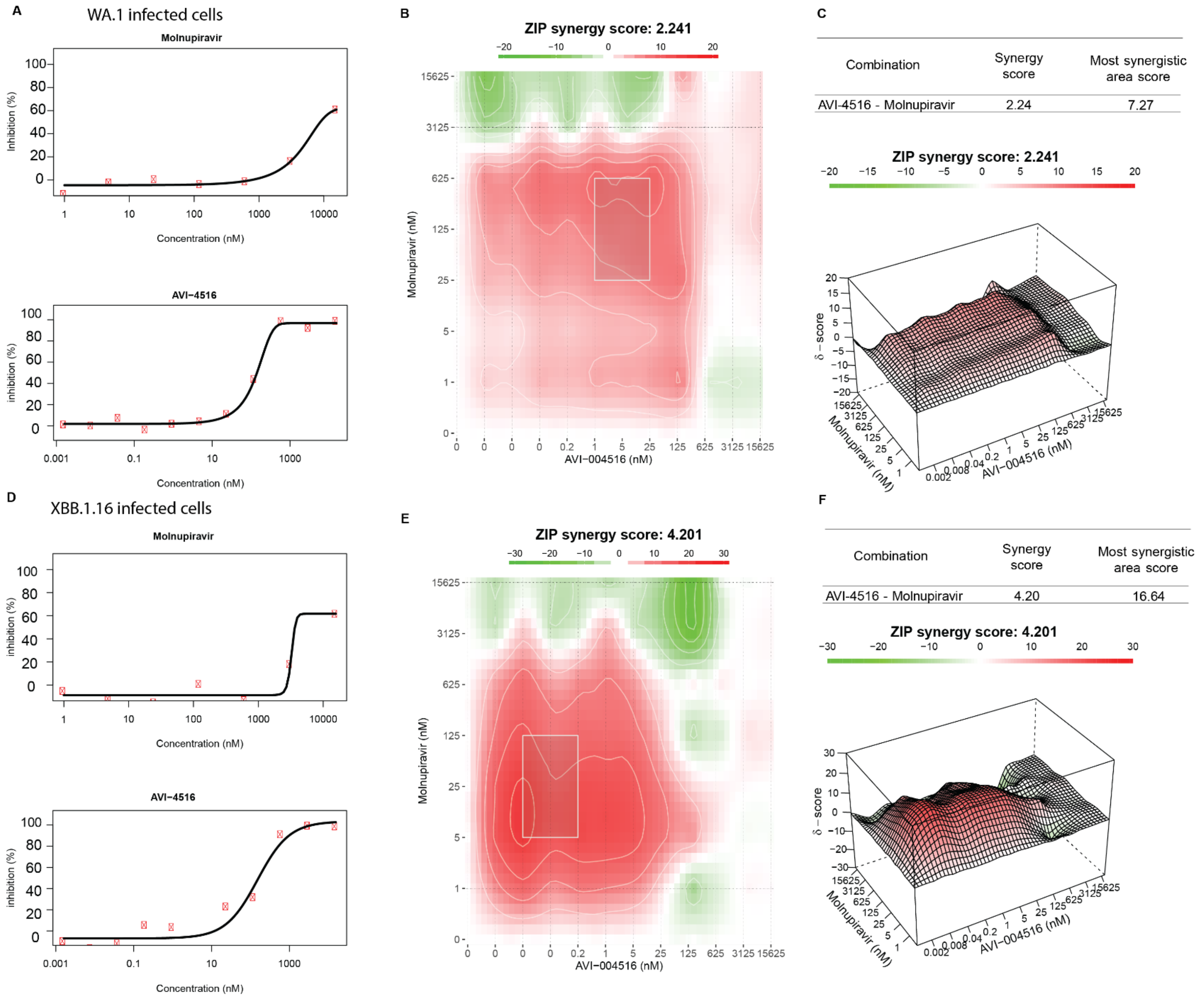


**Supplementary Figure 17**. Synergy experiments with AVI-4516 and molnupiravir. **A-C**. Synergy experiments with AVI-4516 and molnupiravir with WA.1 infected cells. **A**: Dose response for each compound tested. **B**: 2-D plot of ZIP analysis of synergy C: 3-D plot of ZIP analysis of synergy with computed overall synergy score and highest area score. **D-F**: Synergy experiments with AVI-4516 and molnupiravir with XBB.1.16 infected cells. **D:** Dose response for each compound tested and in matrix format. **E**: 2-D plot of ZIP analysis of synergy **F**: 3-D plot of ZIP analysis of synergy with computed overall synergy score and highest area score.

A

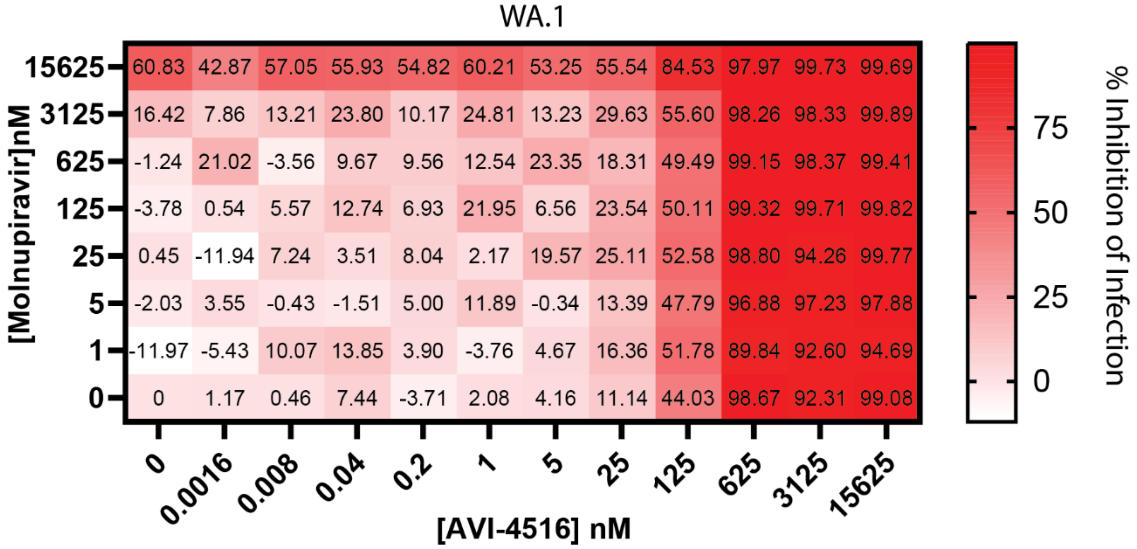


B

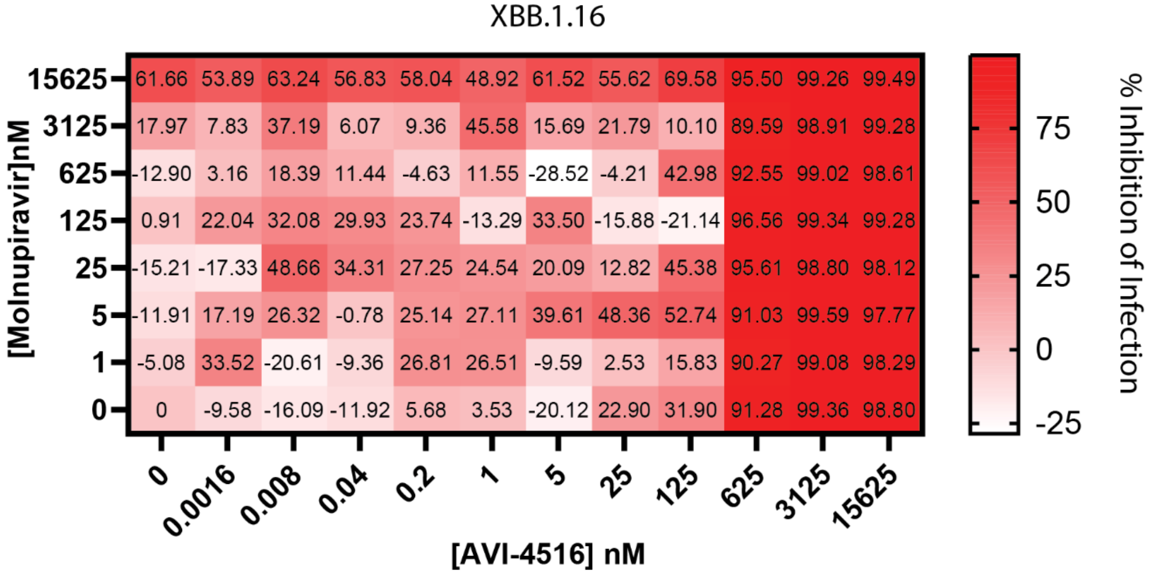


**Supplementary Figure 18.** Dose-response matrix for molnupiravir and AVI-4516 synergy experiments. A Dose-response matrix of molnupiravir and AVI-4516 and the infection inhibition % for cells treated with WA.1. B. Dose response matrix of molnupiravir and AVI-4516 and the infection inhibition % for cells treated with XBB.1.16.

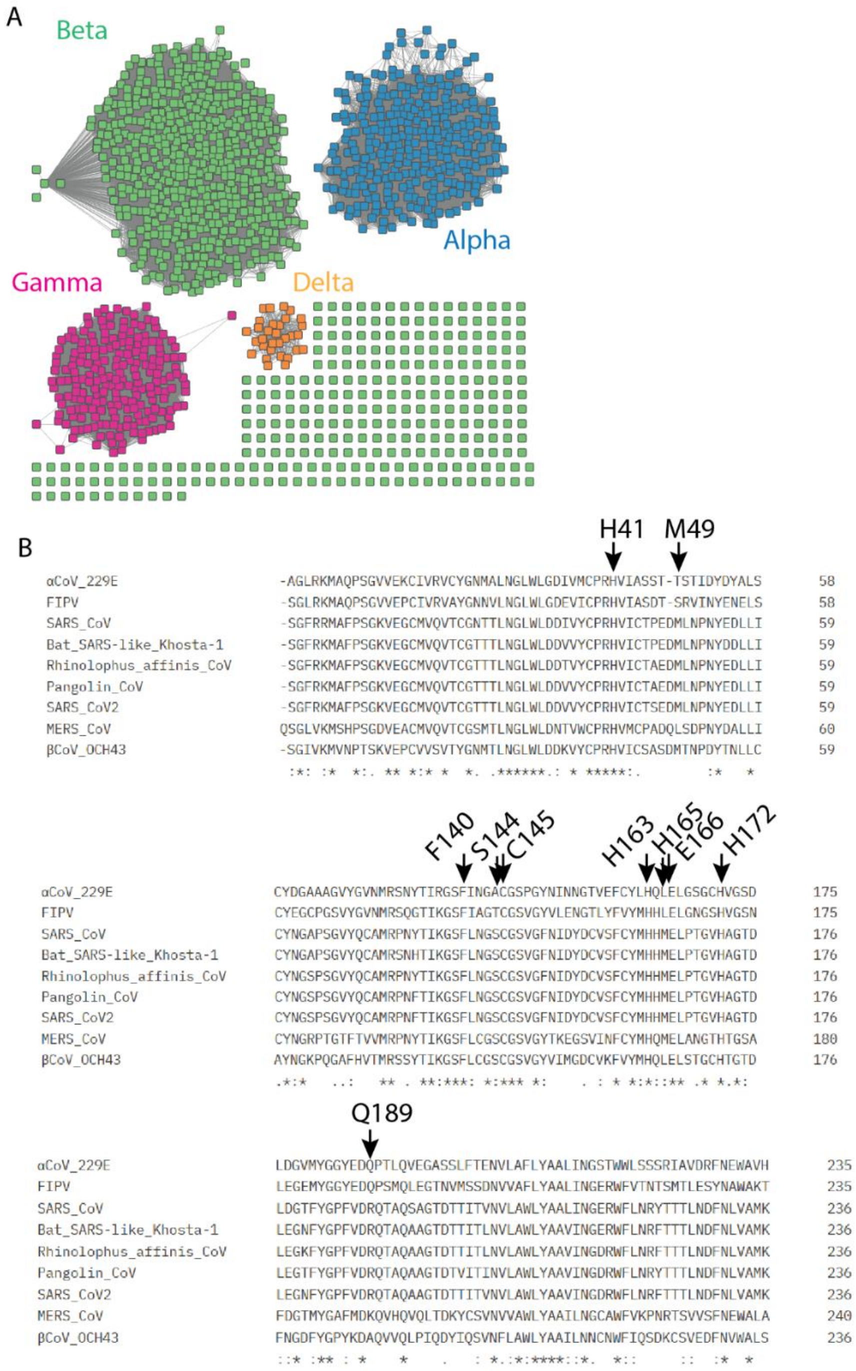


**Supplementary Figure 19.** Sequence similarity network and alignment of various Coronavirus M$^{Pro}$s from the beta and alpha coronavirus families. **A.** a sequence similarity network of coronavirus main proteases with a cutoff of alignment score 1500. Each family is labeled by color beta (green) alpha (blue) Gamma (magenta) and Delta (orange). **B.** An alignment of two Mpros from the alpha coronavirus family 229E, and FIPV, and several beta coronaviruses. Residues that make important contacts or stabilize residues that contact with AVI-4516 and AVI-4692 are highlighted.

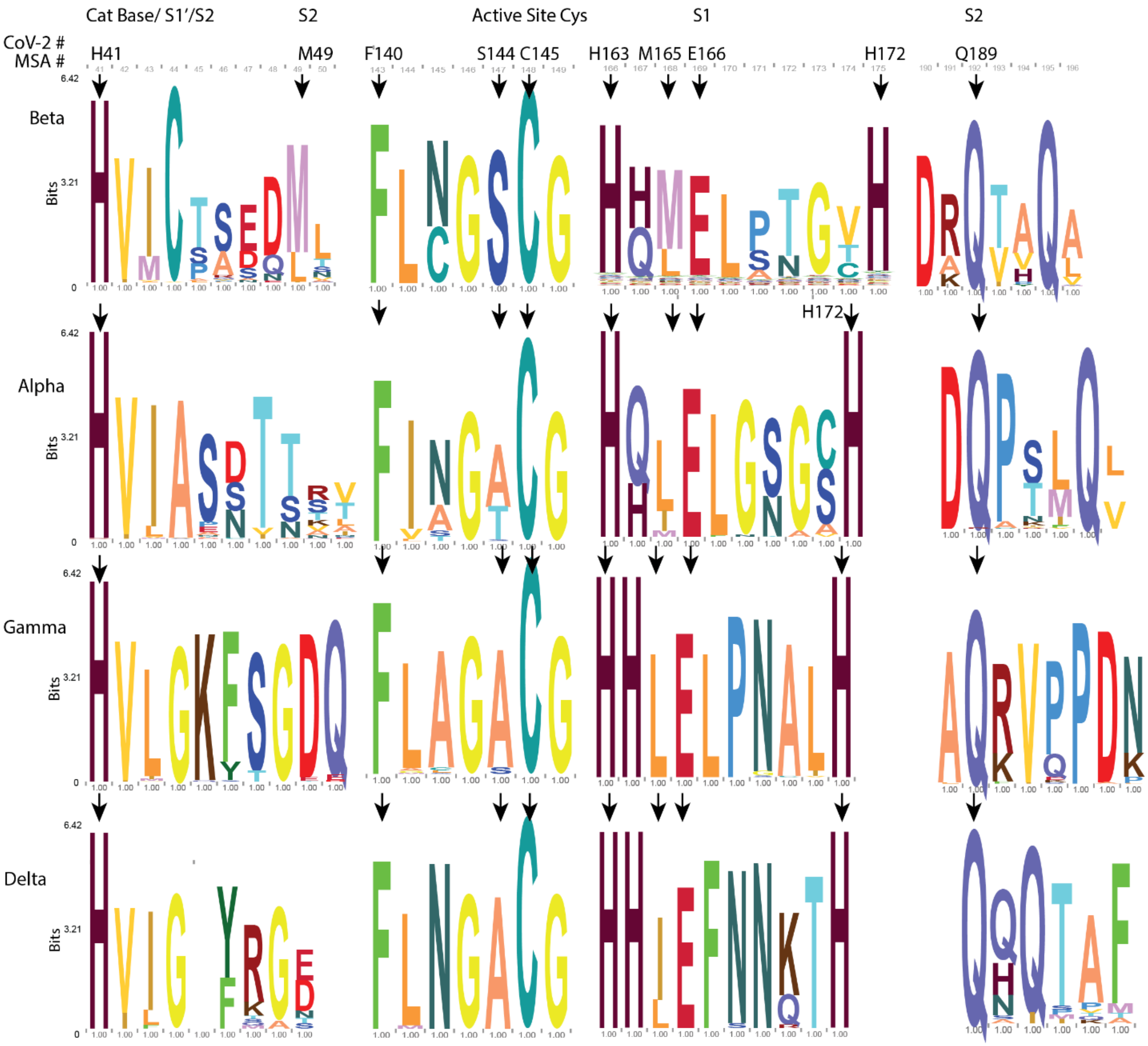


**Supplementary Figure 20.** Logos of residues that contact the compounds across coronavirus families. His 41 is absolutely conserved cross all coronavirus families as it is the catalytic base. The methionine at position 49 in the SARS-CoV-2 $M^{Pro}$ is not as well conserved across the families and a hydrophobic residue is only present in the beta coronavirus family. E166, His 163 and His 172 are all absolutely conserved with the M165 is a linear hydrophobic residue for most sequences.

## Supplementary Tables

**Supplementary Table 1.** Chemical structure and *in vitro* activity of compounds identified by docking. Table contains $IC_{50}$s or % $M^{Pro}$ activity when treated with 100 µM of compound.

| Compound | $IC_{50}$[µM] (% activity at 100 µM) | Compound | $IC_{50}$[µM] (% activity at 100 µM) |
|---|---|---|---|
| 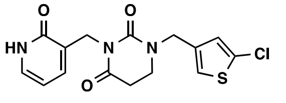 Z8189030022 AVI-3570 | 1.5 | 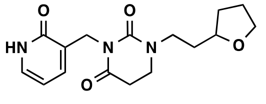 Z3535316691 AVI-3441 | (49%) |
| 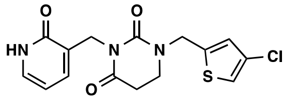 Z3638153574 AVI-3318 | 4.6 | 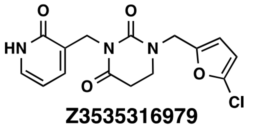 Z3535316979 AVI-3432 | (25%) |
| 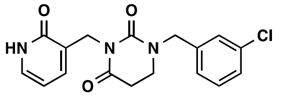 Z8189030055 AVI-3993 | 8.5 | 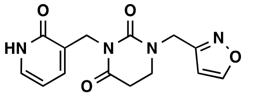 Z3535314312 AVI-3433 | (63%) |
| 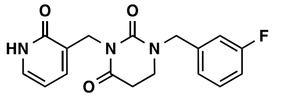 Z8189030074 AVI-3992 | 10.1 | 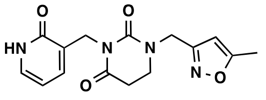 Z3535316272 AVI-3439 | (60%) |
| 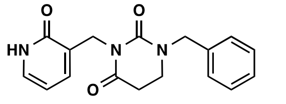 Z7160953946 AVI-3321 | 25.9 | 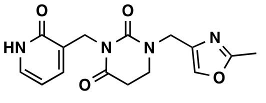 Z3535313327 AVI-3437 | (66%) |
| 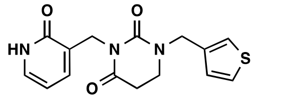 Z3535316454 AVI-3320 | 22.8 | 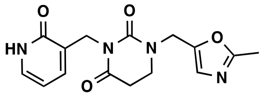 Z3535313803 AVI-3422 | (57%) |
| 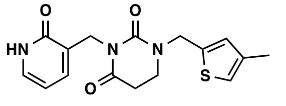 Z3535311658 AVI-3319 | 29.5 | 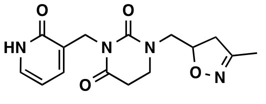 Z3535314281 AVI-3423 | (66%) |
| 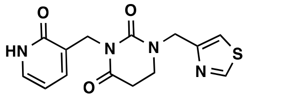 Z3535315305 AVI-3428 | (19%) | 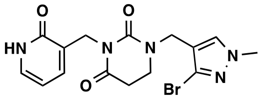 Z3535311170 AVI-3443 | (51%) |
| 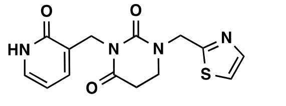 Z3535315936 AVI-3418 | (36%) | 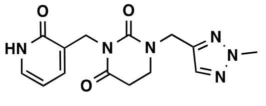 Z7160954841 AVI-3424 | (69%) |

| | | | |
|---|---|---|---|
| 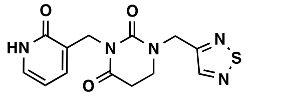 **Z4349493904** **AVI-3419** | (33%) | 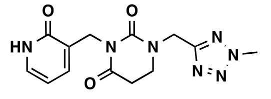 **Z7160954739** **AVI-3435** | (64%) |
| 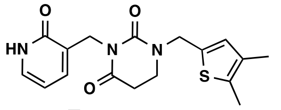 **Z3535313440** **AVI-3440** | (30%) | 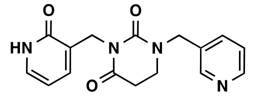 **Z3338086954** **AVI-3436** | (64%) |
| 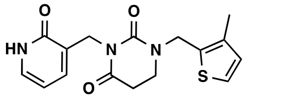 **Z3535316611** **AVI-3425** | (39%) | 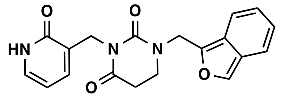 **Z7160954690** **AVI-3415** | (29%) |
| 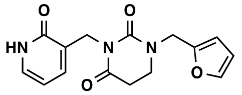 **Z3535317000** **AVI-3444** | (31%) | 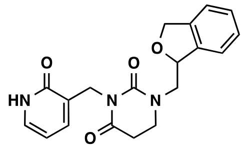 **Z3535311304** **AVI-3431** | (51%) |
| 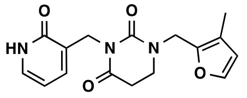 **Z3535315436** **AVI-3429** | (53%) | 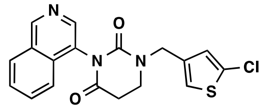 **Z8189030155** **AVI-3778** | 0.85 |
| 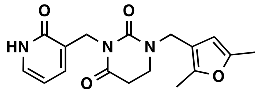 **Z3638153760** **AVI-3416** | (32%) | 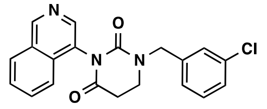 **Z8435235406** **AVI-3779** | 0.66 |
| 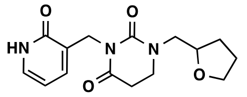 **Z3338086960** **AVI-3420** | (49%) | 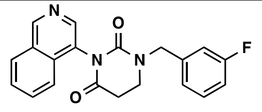 **Z8445588872** **AVI-3780** | 0.8 |

**Supplementary Table 2:** Cytotoxicity concentration 50% ($CC_{50}$) in A549 cells and percent cell viability at 100 µM

| Molecule Name | A549 $CC_{50}$ [µM] | A549 Viability at 100 µM (%) | Molecule Name | A549 $CC_{50}$ [µM] | A549 Viability at 100 µM (%) |
|---|---|---|---|---|---|
| **AVI-3778** | >100 | 95.7 | **AVI-3443** | >100 | 100 |
| **AVI-3779** | >100 | 100 | **AVI-3444** | >100 | 96.9 |
| **AVI-3780** | >100 | 100 | **AVI-3570** | >100 | 100 |
| **AVI-4301** | >100 | 100 | **AVI-4434** | >100 | 100 |
| **AVI-4303** | >100 | 92.3 | **AVI-4435** | >100 | 94.7 |
| **AVI-4434** | >100 | 96.9 | **AVI-4436** | >100 | 99.7 |
| **AVI-4516** | >100 | 90.4 | **AVI-3419** | >100 | 94.4 |
| **AVI-4673** | >100 | 94.4 | **AVI-3420** | >100 | 100 |
| **AVI-1027** | >100 | 100 | **AVI-3421** | >100 | 100 |
| **AVI-1084** | >100 | 100 | **AVI-3422** | >100 | 100 |
| **AVI-1242** | >100 | 100 | **AVI-3423** | >100 | 96.8 |
| **AVI-3318** | >100 | 88.9 | **AVI-3425** | >100 | 94.2 |
| **AVI-3319** | >100 | 100 | **AVI-3426** | >100 | 100 |
| **AVI-3320** | >100 | 100 | **AVI-3428** | >100 | 100 |
| **AVI-3321** | >100 | 100 | **AVI-3429** | >100 | 100 |
| **AVI-3415** | >100 | 100 | **AVI-3430** | >100 | 100 |
| **AVI-3416** | >100 | 100 | **AVI-3431** | >100 | 100 |
| **AVI-3417** | >100 | 100 | **AVI-3432** | >100 | 78.1 |
| **AVI-3418** | >100 | 99.6 | **AVI-3433** | >100 | 98.2 |
| **AVI-3437** | >100 | 100 | **AVI-3434** | >100 | 100 |
| **AVI-3438** | >100 | 70.3 | **AVI-3435** | >100 | 100 |
| **AVI-3439** | >100 | 97.1 | **AVI-3436** | >100 | 100 |
| **AVI-3440** | >100 | 99.1 | **AVI-3441** | >100 | 100 |

**Supplementary Table 3:** Derived apparent permeabilities (Papp) of selected compounds for gut-based Parallel artificial membrane permeability assay **(PAMPA-Gut).**

| **Molecule Name** | **PAMPA-Gut $P_{app}$ ($10^{-6}$cm/s)** |
|---|---|
| **AVI-4143** | 42.3 |
| **AVI-4692** | 31.8 |
| **AVI-3778** | 26.4 |
| **AVI-3779** | 23.9 |
| **AVI-3780** | 19.6 |
| **AVI-4301** | 31.8 |
| **AVI-4303** | 6.6 |
| **AVI-4434** | 12.9 |
| **AVI-4516** | 22.4 |
| **AVI-4673** | 4.99 |

**Supplementary Table 4.** Plasma concentration of AVI-4516 after IV (10mg/kg) and PO (50mg/kg) in male CD-1 mice

**AVI-4516 IV dose 10 mg/kg**

| Dose (mg/kg) | Sampling time (h) | Concentration (ng/mL) Individual | | | Mean (ng/mL) | SD | CV(%) |
|---|---|---|---|---|---|---|---|
| 10 IV | perdose | BQL | BQL | BQL | **BQL** | NA | NA |
| | 0.083 | 7850 | 7710 | 7280 | **7613** | 297 | 3.90 |
| | 0.25 | 4680 | 4520 | 5130 | **4777** | 316 | 6.62 |
| LLOQ=1.00 ng/mL BQL＜LLOQ | 0.5 | 3140 | 3680 | 2250 | **3023** | 722 | 23.9 |
| | 1 | 2080 | 1350 | 2140 | **1857** | 440 | 23.7 |
| | 2 | 615 | 178 | 545 | **446** | 235 | 52.6 |
| | 4 | 43.2 | 96.4 | 30.6 | **56.7** | 34.9 | 61.6 |
| | 8 | 10.2 | 7.28 | 12.9 | **10.1** | 2.81 | 27.8 |
| | 24 | BQL | BQL | BQL | **BQL** | NA | NA |
| PK parameters | Unit | Estimated Value | | | | | |
| CL | L/hr/kg | 1.74 | | | | | |
| $V_{ss}$ | L/kg | 1.39 | | | | | |
| $T_{1/2}$ | hr | 0.866 | | | | | |
| $AUC_{last}$ | hr*ng/mL | 5732 | | | | | |
| $AUC_{INF}$ | hr*ng/mL | 5744 | | | | | |
| $MRT_{INF}$ | hr | 0.798 | | | | | |

**AVI-4516 PO dose 50 mg/kg**

| Dose (mg/kg) | Sampling time (h) | Concentration (ng/mL) Individual | | | Mean (ng/mL) | SD | CV(%) |
|---|---|---|---|---|---|---|---|
| 50 PO | perdose | BQL | BQL | BQL | **BQL** | NA | NA |
| | 0.083 | 5080 | 6540 | 7490 | **6370** | 1214 | 19.1 |
| | 0.25 | 5630 | 3590 | 12100 | **7107** | 4443 | 62.5 |
| LLOQ=1.00 ng/mL BQL＜LLOQ | 0.5 | 8310 | 5590 | 7260 | **7053** | 1372 | 19.4 |
| | 1 | 9050 | 9280 | 12000 | **10110** | 1641 | 16.2 |
| | 2 | 5560 | 4940 | 8730 | **6410** | 2033 | 31.7 |
| | 4 | 7520 | 8490 | 6040 | **7350** | 1234 | 16.8 |
| | 8 | 2630 | 2730 | 3300 | **2887** | 361 | 12.5 |
| | 24 | BQL | BQL | BQL | **BQL** | NA | NA |
| PK parameters | Unit | Estimated Value | | | | | |
| $T_{max}$ | hr | 1.00 | | | | | |
| $C_{max}$ | ng/mL | 10110 | | | | | |
| $T_{1/2}$ | hr | 4.70 | | | | | |
| $AUC_{last}$ | hr*ng/mL | 49944 | | | | | |
| $AUC_{INF}$ | hr*ng/mL | 69535 | | | | | |
| F | % | 174 | | | | | |

**Supplementary Table 5.** Plasma concentration of AVI-4673 after IV (10mg/kg) and PO (50mg/kg) in male CD-1 mice

**AVI-4673 IV dose 10 mg/kg**

| Dose (mg/kg) | Dose route | Sampling time (hr) | Concentration (ng/mL) Individual | | | Mean (ng/mL) | SD | CV(%) |
|---|---|---|---|---|---|---|---|---|
| 10 | IV | perdose | BQL | BQL | BQL | **BQL** | NA | NA |
| | | 0.083 | 4270 | 4830 | 3650 | **4250** | 590 | 13.9 |
| | | 0.25 | 1460 | 2590 | 2120 | **2057** | 568 | 27.6 |
| LLOQ=1.00 ng/mL | | 0.5 | 1020 | 2030 | 1300 | **1450** | 521 | 36.0 |
| BQL＜LLOQ | | 1 | 759 | 791 | 702 | **751** | 45.1 | 6.01 |
| | | 2 | 268 | 300 | 272 | **280** | 17.4 | 6.23 |
| | | 4 | 59.2 | 143 | 81.0 | **94.4** | 43.5 | 46.1 |
| | | 8 | 14.4 | 39.0 | 61.8 | **38.4** | 23.7 | 61.7 |
| | | 24 | BQL | BQL | BQL | **BQL** | NA | NA |

| PK parameters | Unit | Estimated Value |
|---|---|---|
| CL | L/hr/kg | 3.10 |
| $V_{ss}$ | L/kg | 4.75 |
| $T_{1/2}$ | hr | 2.19 |
| $AUC_{last}$ | hr*ng/mL | 3100 |
| $AUC_{INF}$ | hr*ng/mL | 3221 |
| $MRT_{INF}$ | hr | 1.53 |

**AVI-4673 PO dose 50 mg/kg**

| Dose (mg/kg) | Dose route | Sampling time (hr) | Concentration (ng/mL) Individual | | | Mean (ng/mL) | SD | CV(%) |
|---|---|---|---|---|---|---|---|---|
| 50 | PO | perdose | BQL | BQL | BQL | **BQL** | NA | NA |
| | | 0.083 | 35.8 | 87.4 | 132 | **85.1** | 48.1 | 56.6 |
| | | 0.25 | 582 | 323 | 114 | **340** | 234 | 69.0 |
| LLOQ=1.00 ng/mL | | 0.5 | 219 | 1090 | 675 | **661** | 436 | 65.9 |
| BQL＜LLOQ | | 1 | 280 | 1640 | 219 | **713** | 803 | 113 |
| | | 2 | 4500 | 1640 | 1120 | **2420** | 1820 | 75.2 |
| | | 4 | 927 | 2800 | 1120 | **1616** | 1030 | 63.8 |
| | | 8 | 93.9 | 250 | 307 | **217** | 110 | 50.8 |
| | | 24 | 2.45 | 1.72 | 1.44 | **1.87** | 0.521 | 27.9 |

| PK parameters | Unit | Estimated Value |
|---|---|---|
| $T_{max}$ | hr | 2.00 |
| $C_{max}$ | ng/mL | 2420 |
| $T_{1/2}$ | hr | 2.12 |
| $AUC_{last}$ | hr*ng/mL | 11526 |
| $AUC_{INF}$ | hr*ng/mL | 11532 |
| F | % | 71.6 |

**Supplementary Table 6.** Plasma concentration of AVI-4773 after IV (10mg/kg) and PO (50mg/kg) in male CD-1 mice

**AVI-4773 IV dose 10 mg/kg**

| Dose (mg/kg) | Dose route | Sampling time (h) | Concentration (ng/mL) Individual | | | Mean (ng/mL) | SD | CV(%) |
|---|---|---|---|---|---|---|---|---|
| 10 | IV | perdose | BQL | BQL | BQL | **BQL** | NA | NA |
| | | 0.083 | 6340 | 6270 | 5800 | **6137** | 294 | 4.79 |
| | | 0.25 | 6080 | 5700 | 6170 | **5983** | 249 | 4.17 |
| LLOQ =1.00 ng/mL BQL <LLOQ | | 0.5 | 4940 | 5640 | 5030 | **5203** | 381 | 7.32 |
| | | 1 | 4110 | 4500 | 4080 | **4230** | 234 | 5.54 |
| | | 2 | 3920 | 3180 | 3850 | **3650** | 409 | 11.2 |
| | | 4 | 1270 | 1780 | 1030 | **1360** | 383 | 28.2 |
| | | 8 | 84.6 | 186 | 103 | **125** | 54.0 | 43.4 |
| | | 24 | BQL | BQL | BQL | **BQL** | NA | NA |

**AVI-4773 PO dose 50 mg/kg**

| Dose (mg/kg) | Dose route | Sampling time (h) | Concentration (ng/mL) Individual | | | Mean (ng/mL) | SD | CV(%) |
|---|---|---|---|---|---|---|---|---|
| 50 | PO | perdose | BQL | BQL | BQL | **BQL** | NA | NA |
| | | 0.083 | 3040 | 4390 | 1760 | **3063** | 1315 | 42.9 |
| | | 0.25 | 8540 | 8950 | 9020 | **8837** | 259 | 2.93 |
| LLOQ =1.00 ng/mL BQL <LLOQ | | 0.5 | 15100 | 14800 | 14900 | **14933** | 153 | 1.02 |
| | | 1 | 10100 | 13300 | 8230 | **10543** | 2564 | 24.3 |
| | | 2 | 10800 | 12800 | 13900 | **12500** | 1572 | 12.6 |
| | | 4 | 14500 | 9920 | 16900 | **13773** | 3546 | 25.7 |
| | | 8 | 8640 | 9450 | 10300 | **9463** | 830 | 8.77 |
| | | 24 | 3.35 | 1.34 | 7.35 | **4.01** | 3.06 | 76.2 |

| PK parameters | Unit | Estimated Value | PK parameters | Unit | Estimated Value |
|---|---|---|---|---|---|
| CL | L/hr/kg | 0.574 | $T_{max}$ | hr | 0.500 |
| $V_{ss}$ | L/kg | 1.18 | $C_{max}$ | ng/mL | 14933 |
| $T_{1/2}$ | hr | 1.22 | $T_{1/2}$ | hr | 13.0 |
| $AUC_{last}$ | hr*ng/mL | 17200 | $AUC_{last}$ | hr*ng/mL | 94730 |
| $AUC_{INF}$ | hr*ng/mL | 17420 | $AUC_{INF}$ | hr*ng/mL | 272740 |
| $MRT_{INF}$ | hr | 2.06 | F | % | 110 |

**Supplementary Table 7.** Plasma concentration of AVI-4694 after IV (10mg/kg) and PO (50mg/kg) in male CD-1 mice

**AVI-4694 IV dose 10 mg/kg**

| Dose (mg/kg) | Dose route | Sampling time (h) | Concentration (ng/mL) Individual | | | Mean (ng/mL) | SD | CV(%) |
|---|---|---|---|---|---|---|---|---|
| 10 | IV | perdose | BQL | BQL | BQL | **BQL** | NA | NA |
| | | 0.083 | 4850 | 5280 | 4830 | **4987** | 254 | 5.10 |
| | | 0.25 | 4410 | 5030 | 4650 | **4697** | 313 | 6.66 |
| LLOQ=2.00 ng/mL BQL＜LLOQ | | 0.5 | 5160 | 5670 | 5420 | **5417** | 255 | 4.71 |
| | | 1 | 2770 | 3580 | 4060 | **3470** | 652 | 18.8 |
| | | 2 | 2220 | 2450 | 3420 | **2697** | 637 | 23.6 |
| | | 4 | 1710 | 2420 | 2220 | **2117** | 366 | 17.3 |
| | | 8 | 30.8 | 166 | 441 | **213** | 209 | 98.3 |
| | | 24 | BQL | BQL | BQL | **BQL** | NA | NA |

| PK parameters | Unit | Estimated Value |
|---|---|---|
| CL | L/hr/kg | 0.562 |
| $V_{ss}$ | L/kg | 1.46 |
| $T_{1/2}$ | hr | 1.72 |
| $AUC_{last}$ | hr*ng/mL | 17270 |
| $AUC_{INF}$ | hr*ng/mL | 17797 |
| $MRT_{INF}$ | hr | 2.60 |

**AVI-4694 PO dose 50 mg/kg**

| Dose (mg/kg) | Dose route | Sampling time (h) | Concentration (ng/mL) Individual | | | Mean (ng/mL) | SD | CV(%) |
|---|---|---|---|---|---|---|---|---|
| 50 | PO | perdose | BQL | BQL | BQL | **BQL** | NA | NA |
| | | 0.083 | 456 | 112 | 468 | **345** | 202 | 58.5 |
| | | 0.25 | 2020 | 1740 | 3580 | **2447** | 991 | 40.5 |
| LLOQ=2.00 ng/mL BQL＜LLOQ | | 0.5 | 5490 | 4920 | 5530 | **5313** | 341 | 6.42 |
| | | 1 | 6730 | 2070 | 5830 | **4877** | 2472 | 50.7 |
| | | 2 | 4370 | 3630 | 5870 | **4623** | 1141 | 24.7 |
| | | 4 | 6470 | 5940 | 4890 | **5767** | 804 | 13.9 |
| | | 8 | 1460 | 5280 | 1070 | **2603** | 2326 | 89.4 |
| | | 24 | 3.88 | 4.05 | 5.25 | **4.39** | 0.747 | 17.0 |

| PK parameters | Unit | Estimated Value |
|---|---|---|
| $T_{max}$ | hr | 4.00 |
| $C_{max}$ | ng/mL | 5767 |
| $T_{1/2}$ | hr | 1.87 |
| $AUC_{last}$ | hr*ng/mL | 56507 |
| $AUC_{INF}$ | hr*ng/mL | 56519 |
| F | % | 63.5 |

**Supplementary Table 8**. AVI-4516 tissue distribution after 100 mg/kg PO dose

| Sampling time (h) | Plasma (ng/mL) | | | | Brain (ng/g) | | | | Kidney (ng/g) | | | |
|---|---|---|---|---|---|---|---|---|---|---|---|---|
| | | | | Average | | | | Average | | | | Average |
| 0.25 | 3.57* | 10400 | 10000 | **10200** | 17.1* | 805 | 666 | **736** | 8.86* | 22600 | 22700 | **22650** |
| 2 | 7080 | 9120 | 8370 | **8190** | 542 | 671 | 609 | **607** | 16300 | 18800 | 15400 | **16833** |
| 8 | 6100 | 3870 | 7970 | **5980** | 471 | 262 | 500 | **411** | 12700 | 7500 | 19200 | **13133** |

| Sampling time (h) | Liver (ng/g) | | | | Heart (ng/g) | | | |
|---|---|---|---|---|---|---|---|---|
| | | | | Average | | | | Average |
| 0.25 | 38.2* | 72000 | 45700 | **58850** | 9.05* | 15200 | 13400 | **14300** |
| 2 | 28700 | 29700 | 29000 | **29133** | 10900 | 12300 | 10800 | **11333** |
| 8 | 27100 | 17200 | 38200 | **27500** | 8970 | 5060 | 12900 | **8977** |

*=below limit of quantification

| Sampling time (h) | Plasma (ng/mL) | | | | Lung (ng/g) | | | |
|---|---|---|---|---|---|---|---|---|
| | | | | Average | | | | Average |
| 0.25 | 23400 | 18100 | 9870 | **17123** | 13000 | 10000 | 6270 | **9757** |
| 2 | 11000 | 9310 | 7880 | **9397** | 5090 | 4200 | 3200 | **4163** |
| 8 | 3750 | 4380 | 4390 | **4173** | 2650 | 1540 | 1410 | **1867** |

| Sampling time (h) | BALF (ng/mL) | | | | BALF (Sample Volume (mL) | | | |
|---|---|---|---|---|---|---|---|---|
| | | | | Average | | | | Average |
| 0.25 | 3250 | 2810 | 1190 | **2417** | 0.510 | 0.330 | 0.390 | **0.410** |
| 2 | 1420 | 1190 | 1020 | **1210** | 0.800 | 0.710 | 0.750 | **0.753** |
| 8 | 607 | 398 | 396 | **467** | 0.460 | 0.490 | 0.710 | **0.553** |

| Supplemental Table 9. Brain Distrubition of Ensitrelvir after a 100 mg/kg PO dose in male CD1 mouse | | | | | | |
|---|---|---|---|---|---|---|
| Dose (mg/kg) | Dose route | Sampling time (hr) | Concentration (ng/g) | | | **Mean (ng/g)** |
| | | | Individual | | | |
| 100 | PO | 0.25 | 1540 | 1280 | 1970 | **1597** |
| | | 2 | 1190 | 1590 | 1780 | **1520** |
| | | 8 | 1200 | 914 | 2150 | **1421** |
| | | 24 | 29.3 | 46.8 | 67.7 | **47.9** |

**Supplementary Table 10**. AVI-4773 tissue distribution after 100 mg/kg PO dose

| Sampling time (h) | Plasma (ng/mL) | | | | Brain (ng/g) | | | | Kidney (ng/g) | | | |
|---|---|---|---|---|---|---|---|---|---|---|---|---|
| | | | | Average | | | | Average | | | | Average |
| 0.25 | 25100 | 30600 | 20600 | 25433 | 3350 | 4060 | 2100 | 3170 | 93100 | 136000 | 83800 | 104300 |
| 2 | 19800 | 32600 | 28900 | 27100 | 2480 | 5990 | 4110 | 4193 | 60100 | 113000 | 131000 | 101367 |
| 8 | 13300 | 27400 | 18200 | 19633 | 1240 | 2890 | 2150 | 2093 | 57200 | 68700 | 83900 | 69933 |

| Sampling time (h) | Liver (ng/g) | | | | Heart (ng/g) | | | |
|---|---|---|---|---|---|---|---|---|
| | | | | Average | | | | Average |
| 0.25 | 127000 | 168000 | 146000 | 147000 | 91500 | 62400 | 48300 | 67400 |
| 2 | 78600 | 146000 | 135000 | 119867 | 42100 | 90700 | 80400 | 71067 |
| 8 | 59400 | 97700 | 76600 | 77900 | 30000 | 55400 | 44700 | 43367 |

*=below limit of quantification

| Sampling time (h) | Lung (ng/g) | | | |
|---|---|---|---|---|
| | | | | Average |
| 0.25 | 28400 | 20200 | 17900 | 22167 |
| 2 | 15500 | 28000 | 24000 | 22500 |
| 8 | 7910 | 15300 | 13000 | 12070 |

| Sampling time (h) | BALF (ng/mL) | | | | BALF (Sample Volume (mL) | | | |
|---|---|---|---|---|---|---|---|---|
| | | | | Average | | | | Average |
| 0.25 | 2880 | 2020 | 3180 | 2880 | 0.850 | 0.870 | 0.500 | 0.740 |
| 2 | 2600 | 4260 | 3630 | 2600 | 0.720 | 0.860 | 0.890 | 0.823 |
| 8 | 1250 | 2910 | 1750 | 1250 | 0.790 | 0.870 | 0.830 | 0.830 |

**Supplementary Table 11.** Pan-coronavirus activity of $M^{Pro}$ inhibitors. EC50 and EC90 are effective concentrations for 50% and 90% reduction in CPE, respectively. CC50 is cytotoxic concentration 50% viability of cells. SI50 and SI90 are selectivity indices defined by EC50 divided by CC50 and EC90 divided by CC50, respectively. Further description can be found in the methods.

| Compound Name | Virus | Virus Strain | Cell line | Test Compound | | | | | Positive Control | | | | |
|---|---|---|---|---|---|---|---|---|---|---|---|---|---|
| | | | | $EC_{50}$ | $EC_{90}$ | $CC_{50}$ | $SI_{50}$ | $SI_{90}$ | $EC_{50}$ | $EC_{90}$ | $CC_{50}$ | $SI_{50}$ | $SI_{90}$ |
| AVI - 4516 | HCoV | Alpha 229E | Huh7 | 2.9 | 3.5 | >100 | >34 | 29 | 0.44 | 0.37 | >10 | >23 | >27 |
| AVI - 4694 | | | | >1.6 | >1.6 | 1.6 | 0 | 0 | 0.44 | 0.37 | >10 | >23 | >27 |
| AVI - 4516 | HCoV | Beta OC43 | Rhabdomyosarcoma | 0.59 | 0.52 | >100 | >170 | >190 | 0.035 | 0.032 | 6.1 | 170 | 190 |
| AVI - 4694 | | | | 0.055 | 0.061 | >100 | >1800 | >1600 | 0.035 | 0.032 | 6.1 | 170 | 190 |
| AVI - 4516 | MERS-CoV | EMC | Vero E6 | <0.032 | 0 | >100 | >3200 | 2900 | 0.0037 | 0.039 | 2 | 540 | 51 |
| AVI - 4694 | | | | 0.045 | 0 | >100 | >2200 | 1300 | 0.0037 | 0.039 | 2 | 540 | 51 |
| AVI - 4516 | SARS-CoV | Urbani | | 0.57 | 1 | >100 | >180 | >100 | 0.12 | 0.14 | >10 | >83 | >71 |
| AVI - 4694 | | | | 0.052 | 0.091 | 54 | 1000 | 590 | 0.12 | 0.14 | >10 | >83 | >71 |
| AVI - 4516 | SARS-CoV-2 | B. 1. 617. 2 (delta) | | 0.24 | 0.18 | >100 | >420 | 560 | 0.17 | 0.05 | 36 | 210 | 720 |
| AVI - 4694 | | | | 0.063 | 0.047 | 32 | 510 | 680 | 0.17 | 0.05 | 36 | 210 | 720 |
| AVI - 4516 | | BA.2 (omicron) | | 0.18 | 0.39 | >100 | >560 | 260 | 0.053 | 0.043 | 43 | 810 | 1000 |
| AVI - 4694 | | | | 0.05 | 0.045 | 41 | 820 | 910 | 0.053 | 0.043 | 43 | 810 | 1000 |

**Supplementary Table 12:** Refinement statistics for X-ray diffraction data and protein models

| Compound | AVI-3318 | AVI-4516 | AVI-4692 | AVI-4303 |
|---|---|---|---|---|
| PDB entry ID | 9MVM | 9MVP | 9MVO | 9MVQ |
| Data collection | | | | |
| Wavelength (Å) | 0.88557 | 1.12709 | 1.12709 | 1.115830 |
| Resolution (Å) | 50.00 - 1.96 | 50.00 - 2.35 | 50.00 - 1.85 | 50.00 - 1.58 |
| Space group | C 1 2 1 | C 1 2 1 | C 1 2 1 | P $3_2$ 2 1 |
| Unit cell dimensions | | | | |
| *a, b, c* (Å) | 115.5 53.8 45.2 | 115.2 53.7 45.0 | 114.8 53.5 44.8 | 66.4 66.4 233.6 |
| *α, β, γ* (°) | 90 101 90 | 90 101.5 90 | 90 102.1 90 | 90 90 120 |
| Total number of reflections ([a]) | 475284 (27923) | 66793 (10390) | 155577 (25174) | 1159359 (186246) |
| Unique reflections ([a]) | 17848 (1774) | 11344 (1752) | 23010 (2253) | 84920 (13483) |
| Multiplicity ([a]) | 10.06 (15.74) | 5.88 (5.93) | 6.18 (6.29) | 13.6 (13.8) |
| Completeness (%) ([a]) | 90 (56) | 99.1 (96.0) | 99.6 (98.8) | 99.9 (99.4) |
| Mean I/σ(I) ([a]) | 45.13 (2.96) | 13.39 (1.96) | 21.74 (2.57) | 25.88 (3.31) |
| $R_{merge}$ (%) ([a]) | 4.7 (83.5) | 10.1 (81.0) | 5.3 (77.6) | 5.7 (76) |
| $R_{meas}$ (%) ([a]) | 4.8 (86.2) | 11.1 (88.9) | 5.7 (83.9) | 6.0 (78.9) |
| $CC_{1/2}$ (%) ([a]) | 100 (89) | 99.7 (72.1) | 100 (86.4) | 100 (91.6) |
| Wilson B-factor ($Å^2$) | 40.22 | 47.29 | 30.61 | 21.14 |
| Refinement | | | | |
| Resolution (Å) ([a]) | 44.44 - 1.96 (2.03 - 1.96) | 48.59 - 2.35 (2.43 - 2.35) | 38.68 - 1.84 (1.91 - 1.84) | 46.29 - 1.57 (1.62 - 1.57) |
| Reflections used ([a]) | 17809 (962) | 11339 (1075) | 23012 (2253) | 84857 (8380) |
| Reflections used for $R_{free}$ ([a]) | 862 (42) | 557 (56) | 2000 (196) | 4166 (371) |
| $R_{work}$ (%) ([a]) | 0.20 (0.29) | 0.20 (0.30) | 0.17 (0.28) | 0.15 (0.17) |
| $R_{free}$ (%) ([a]) | 0.24 (0.36) | 0.25 (0.44) | 0.21 (0.38) | 0.20 (0.26) |

| | | | | |
|---|---|---|---|---|
| Number of non-hydrogen atoms | 2398 | 2387 | 2630 | 5380 |
| macromolecules | 2342 | 2328 | 2379 | 4774 |
| ligands | 23 | 37 | 76 | 73 |
| solvent | 33 | 22 | 175 | 530 |
| Protein residues | 305 | 305 | 305 | 606 |
| RMS (bonds) (Å) | 0.049 | 0.047 | 0.033 | 0.042 |
| RMS (angles) (°) | 1.14 | 1.42 | 1.80 | 1.08 |
| Ramachandran plot | | | | |
| favored (%) | 98.33 | 96.33 | 98.00 | 97.67 |
| allowed (%) | 1.33 | 3.33 | 2.00 | 2.33 |
| outliers (%) | 0.33 | 0.33 | 0.00 | 0.00 |
| rotamer outlier (%) | 0.39 | 0.40 | 0.00 | 0.74 |
| Clashscore | 5.17 | 2.17 | 4.18 | 2.41 |
| Average B factor (Å) | 45.06 | 54.79 | 34.06 | 29.75 |
| macromolecules | 45.11 | 54.80 | 33.48 | 28.77 |
| ligands | 43.19 | 58.03 | 36.52 | 31.30 |
| solvent | 43.03 | 48.12 | 40.93 | 38.36 |

([a]) Values in parentheses correspond to the highest-resolution shell

**Supplementary Table 13**. Eurofins peptidase and pharmacology screen information.

| EUROFINS ASSAY CATALOG NUMBER | Ascii Assay Name | Ligand or Substrate | Species | Tissue |
|---|---|---|---|---|
| 104010 | Cholinesterase, Acetyl, ACES | Acetylthiocholine | Human | recombinant |
| 107371 | Peptidase Bleomycin Hydrolase (BLMH) | Met-AMC | Human | recombinan |
| 108010 | Peptidase, CAN1 (CANPL1, Calpain-1) | Casein-FITC | Human | erythrocytes |
| 112250 | Peptidase, CTSB (Cathepsin B) | Boc-Leu-Arg-Arg-AMC | Human | liver |
| 112510 | Peptidase, CTSG (Cathepsin G) | Suc-Ala-Ala-Pro-Phe-AMC | Human | neutrophils |
| 112550 | Peptidase, CTSH (Cathepsin H) | L-Arg-AMC | Human | liver |
| 112600 | Peptidase, CTSK (Cathepsin K) | Z-Phe-Arg-AMC | Human | recombinant |
| 112650 | Peptidase, CTSL (Cathepsin L) | Z-Phe-Arg-AMC | Human | liver |
| 112750 | Peptidase, CTSS (Cathepsin S) | Z-Leu-Arg-AMC | Human | recombinant |
| 112800 | Peptidase, CTSL2 (Cathepsin L2) | Z-Leu-Arg-AMC | Human | recombinant |
| 112900 | Peptidase, CTSZ (Cathepsin Z) | Mca-Arg-Pro-Pro-Gly-Phe-Ser-Ala-Phe-Lys(Dnp)-OH | Human | recombinant |
| 113310 | Peptidase, Chymase | Suc-Ala-Ala-Pro-Phe-AMC | Human | recombinant |
| 113400 | Peptidase, Chymotrypsin | Suc-Ala-Ala-Pro-Phe-AMC | Human | pancreas |
| 113500 | Peptidase, Factor VIIa | N-CH3-SO2-D-Phe-Gly-Arg-pNA | Human | plasma |
| 113600 | Peptidase, Factor Xa | N-alpha-Z-D-Arg-Gly-Arg-pNA | Human | plasma |
| 113800 | Peptidase, Kallikrein, Plasma | H-D-Pro-Phe-Arg-pNA | Human | plasma |
| 115900 | Peptidase, PLAU (Urokinase) | Z-Gly-Gly-Arg-AMC | Human | Urine |
| 116030 | Cyclooxygenase COX-1 | Arachidonic acid | Human | recombinant |
| 118030 | Cyclooxygenase COX-2 | Arachidonic Acid | Human | recombinant |
| 140010 | Monoamine Oxidase MAO-A | Kynuramine | Human | recombinant |
| 152300 | Phosphodiesterase PDE3A | FAM-cAMP | Human | recombinant |
| 154420 | Phosphodiesterase PDE4D2 | FAM-cAMP | Human | recombinant |
| 163100 | Peptidase, CASP2 (Caspase 2) | Z-VDVAD-AFC | Human | recombinant |
| 163240 | Peptidase, CASP4 (Caspase 4) | Ac-LEVD-AFC | Human | recombinant |
| 163280 | Peptidase, CASP5 (Caspase 5) | Ac-WEHD-AFC | Human | recombinant |
| 164400 | Peptidase, Prolyl Oligopeptidase (POP) | Z-Gly-Pro-pNA | Bacteria | |
| 164500 | Peptidase, Plasmin | H-D-Val-Leu-Lys-pNA | Human | plasma |
| 165000 | Peptidase, Thrombin | Z-Gly-Pro-Arg-AMC | Human | plasma |
| 165050 | Peptidase, Tissue Plasminogen Activator (tPA) | N-alpha-Z-D-Arg-Gly-Arg-pNA | Human | Melanoma cell |

| | | | | |
|---|---|---|---|---|
| **165100** | Peptidase, Trypsin | Z-Gly-Pro-Arg-AMC | Human | pancreas |
| **165200** | Peptidase, Tryptase | Z-Gly-Pro-Arg-AMC | Human | recombinant |
| **166010** | Peptidase, ELA2 (Neutrophil Elastase 2) | N-MeOSuc-Ala-Ala-Pro-Val-pNA | Human | neutrophils |
| **166050** | Peptidase, ELA1 (Pancreatic Elastase 1) | N-MeOSuc-Ala-Ala-Pro-Val-pNA | Pig | pancreas |
| **176020** | Protein Tyrosine Kinase, LCK | Poly(Glu:Tyr) | Human | recombinant |
| **199007** | Peptidase, Dipeptidyl Peptidase 4 (DPP4, DPP IV) | GP-AMC | Human | recombinant |
| **199011** | Peptidase, CASP1 (Caspase 1) | Z-YUAD-AFC | Human | recombinant |
| **200610** | Adenosine A2A | [3H]CGS-21680 | Human | recombinant |
| **203110** | Adrenergic alpha1A | [3H]Prazosin | Human | recombinant |
| **203630** | Adrenergic alpha2A | [3H]Rauwolscine | Human | recombinant |
| **204010** | Adrenergic beta1 | [125I]Cyanopindolol | Human | recombinant |
| **204110** | Adrenergic beta2 | [3H]CGP-12177 | Human | recombinant |
| **204410** | Transporter, Norepinephrine (NET) | [125I]RTI-55 | Human | recombinant |
| **206000** | Androgen (Testosterone) | [3H]Methyltrienolone | Human | LNCaP clone FGC cells |
| **214600** | Calcium Channel L-Type, Dihydropyridine | [3H]Nitrendipine | Rat | cerebral cortex |
| **217050** | Cannabinoid CB1 | [3H]SR141716A | Human | recombinant |
| **217100** | Cannabinoid CB2 | [3H]WIN-55,212-2 | Human | recombinant |
| **218030** | Cholecystokinin CCK1 (CCKA) | [125I]CCK-8 | Human | recombinant |
| **219500** | Dopamine D1 | [3H]SCH-23390 | Human | recombinant |
| **219700** | Dopamine D2S | [3H]Spiperone | Human | recombinant |
| **220320** | Transporter, Dopamine (DAT) | [125I]RTI-55 | Human | recombinant |
| **224010** | Endothelin ETA | [125I]Endothelin-1 | Human | recombinant |
| **226600** | GABAA, Flunitrazepam, Central | [3H]Flunitrazepam | Rat | brain (minus cerebellum) |
| **232030** | Glucocorticoid | [3H]Dexamethasone | Human | recombinant |
| **232810** | Glutamate, NMDA, Agonism | [3H]CGP-39653 | Rat | cerebral cortex |
| **239610** | Histamine H1 | [3H]Pyrilamine | Human | recombinant |
| **239710** | Histamine H2 | [125I]Aminopotentidine | Human | recombinant |
| **252610** | Muscarinic M1 | [3H]N-Methylscopolamine | Human | recombinant |
| **252710** | Muscarinic M2 | [3H]N-Methylscopolamine | Human | recombinant |

| | | | | |
|---|---|---|---|---|
| **252810** | Muscarinic M3 | [3H]N-Methylscopolamine | Human | recombinant |
| **260130** | Opiate delta1 (OP1, DOP) | [3H]Naltrindole | Human | recombinant |
| **260210** | Opiate kappa (OP2, KOP) | [3H]Diprenorphine | Human | recombinant |
| **260410** | Opiate mu (OP3, MOP) | [3H]Diprenorphine | Human | recombinant |
| **265510** | Potassium Channel [KA] | [125I]alpha-Dendrotoxin | Rat | cerebral cortex |
| **265910** | Potassium Channel hERG, [3H]Dofetilide | [3H]Dofetilide | Human | recombinant |
| **271110** | Serotonin (5-Hydroxytryptamine) 5-HT1A | [3H]8-OH-DPAT | Human | recombinant |
| **271230** | Serotonin (5-Hydroxytryptamine) 5-HT1B | [3H]GR125743 | Human | recombinant |
| **271650** | Serotonin (5-Hydroxytryptamine) 5-HT2A | [3H]Ketanserin | Human | recombinant |
| **271700** | Serotonin (5-Hydroxytryptamine) 5-HT2B | [3H]Lysergic acid diethylamide (LSD) | Human | recombinant |
| **271910** | Serotonin (5-Hydroxytryptamine) 5-HT3 | [3H]GR-65630 | Human | recombinant |
| **274030** | Transporter, Serotonin (5-Hydroxytryptamine) (SERT) | [3H]Paroxetine | Human | recombinant |
| **279510** | Sodium Channel, Site 2 | [3H]Batrachotoxinin | Rat | brain (minus cerebellum) |
| **287530** | Vasopressin V1A | [125I]PhenylacetylTyr(Me)PheGlnAsnArgProArgTyr | Human | recombinant |
| **299031** | Nicotinic Acetylcholine alpha4beta2, Cytisine | [3H]Cytisine | Human | recombinant |

**NMR Spectra of Final Compounds**

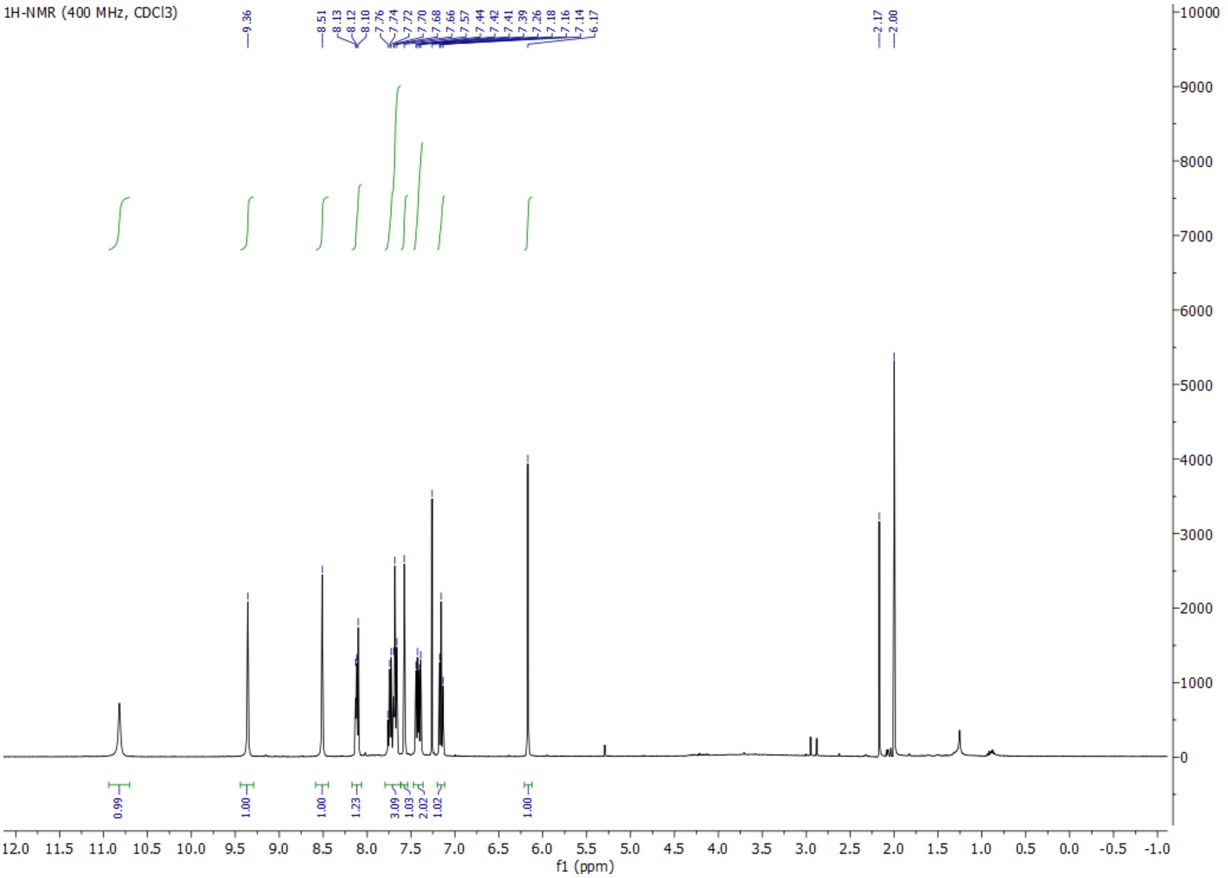


$^{13}$C-NMR (101 MHz, DMSO-d6)

1H-NMR (400 MHz, DMSO-d6)

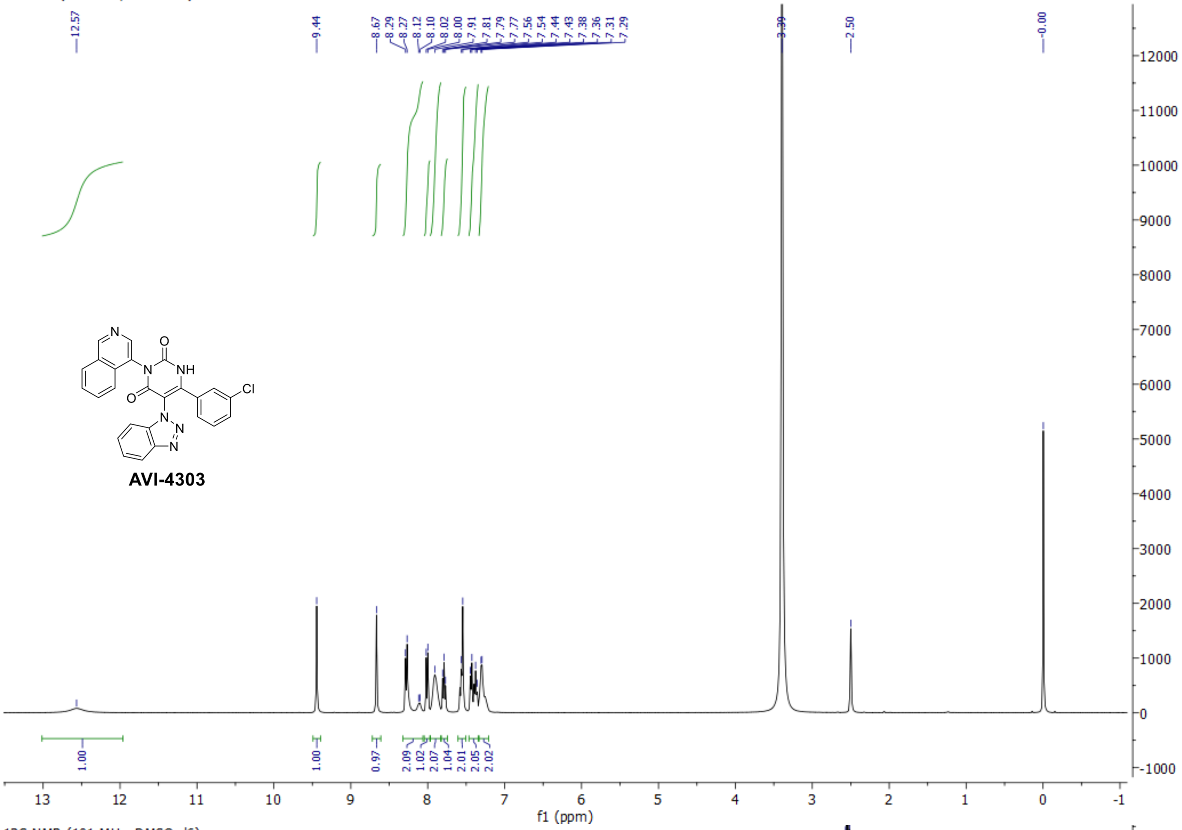

13C-NMR (101 MHz, DMSO-d6)

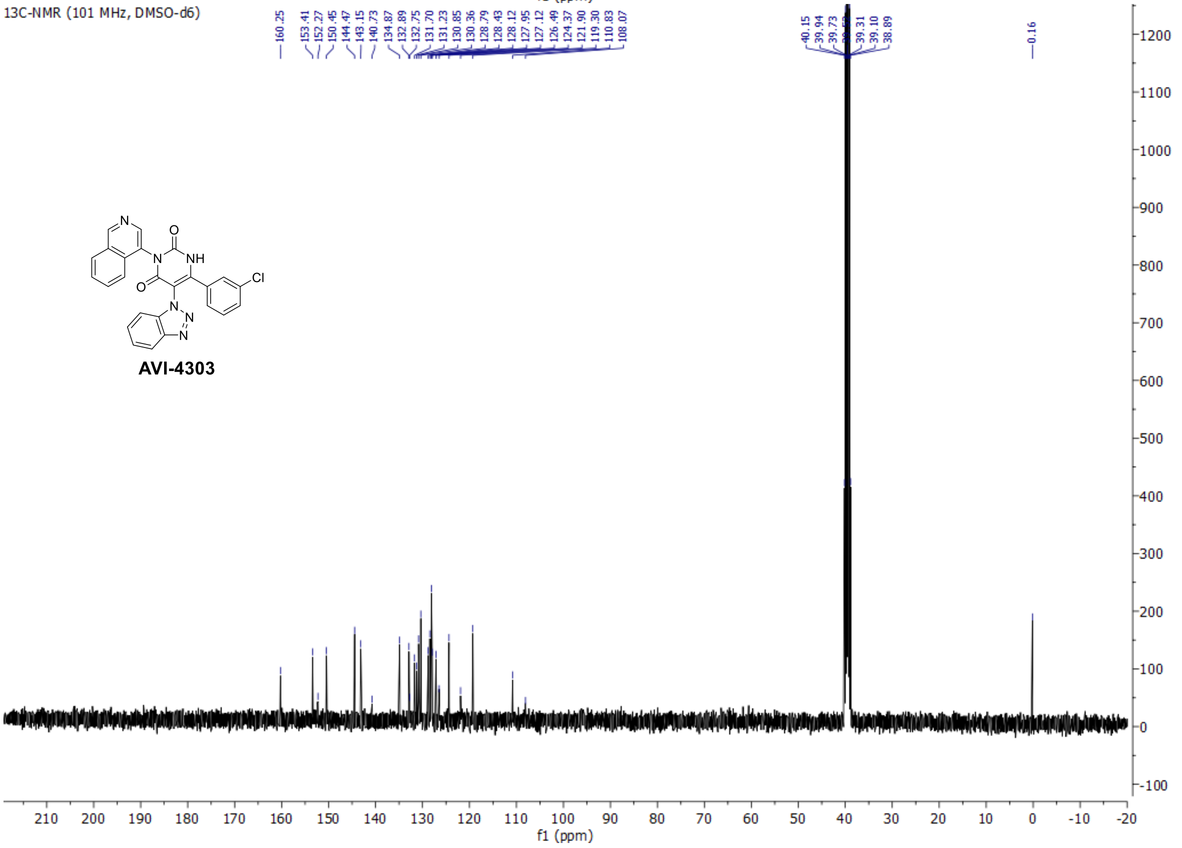

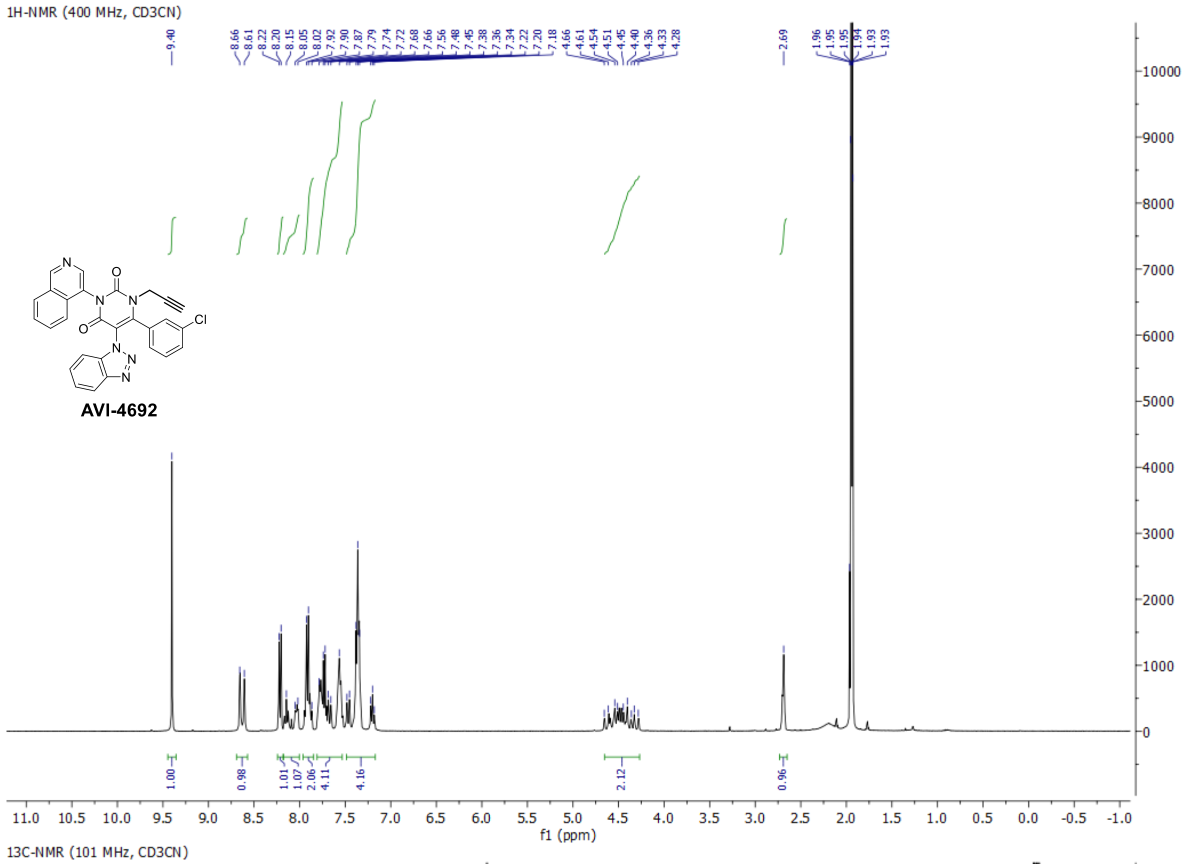
1H-NMR (400 MHz, CD3CN)
9.40
8.66
8.61
8.22
8.20
8.15
8.05
8.02
7.92
7.90
7.87
7.79
7.74
7.72
7.68
7.66
7.56
7.48
7.45
7.38
7.36
7.34
7.22
7.20
7.18
4.66
4.61
4.54
4.51
4.45
4.40
4.36
4.33
4.28
2.69
1.96
1.95
1.95
1.94
1.93
1.93
1.00
0.98
1.01
1.07
2.06
4.11
4.16
2.12
0.96
AVI-4692
f1 (ppm)

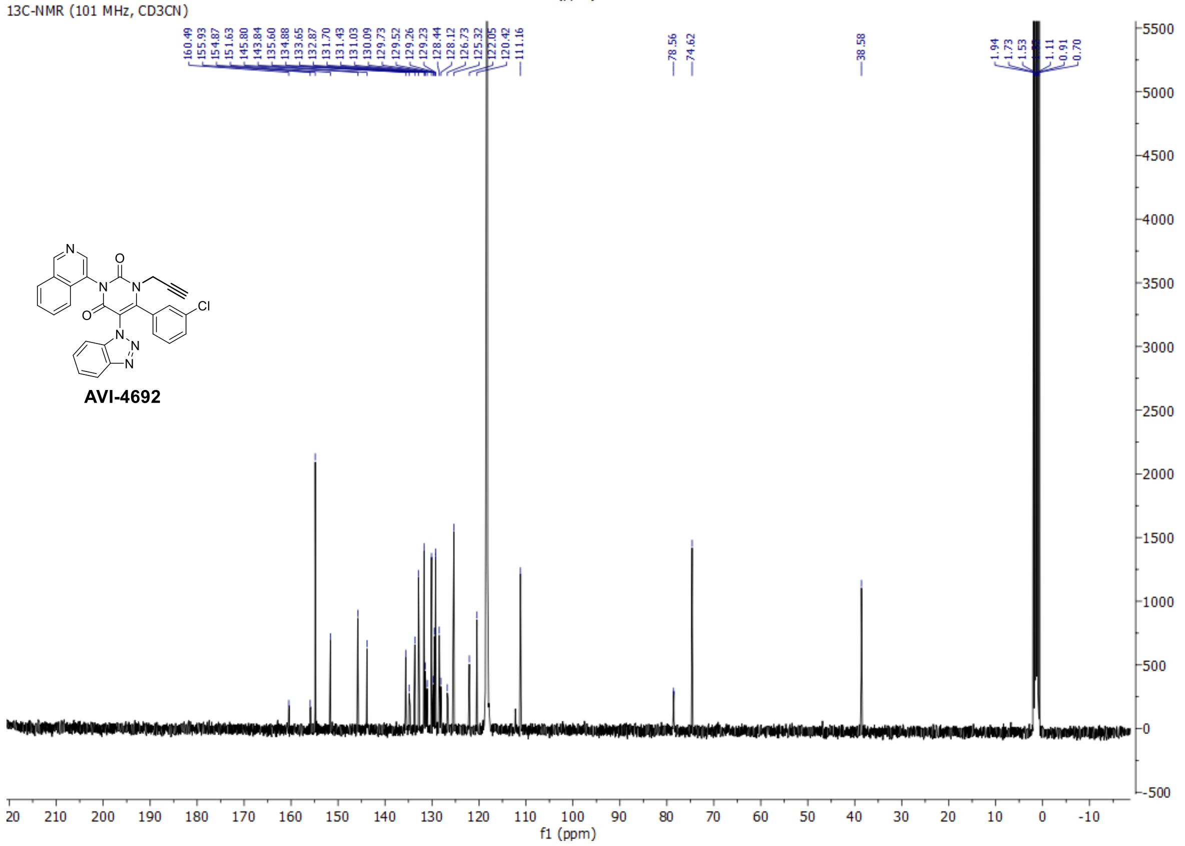
13C-NMR (101 MHz, CD3CN)
160.49
155.93
154.87
151.63
145.80
143.84
135.60
134.88
133.65
132.87
131.70
131.43
131.03
130.09
129.73
129.52
129.26
129.23
128.44
128.12
126.73
125.32
122.05
120.42
111.16
78.56
74.62
38.58
1.94
1.73
1.53
1.11
0.91
0.70
AVI-4692
f1 (ppm)

1H-NMR (400 MHz, DMSO-d6)

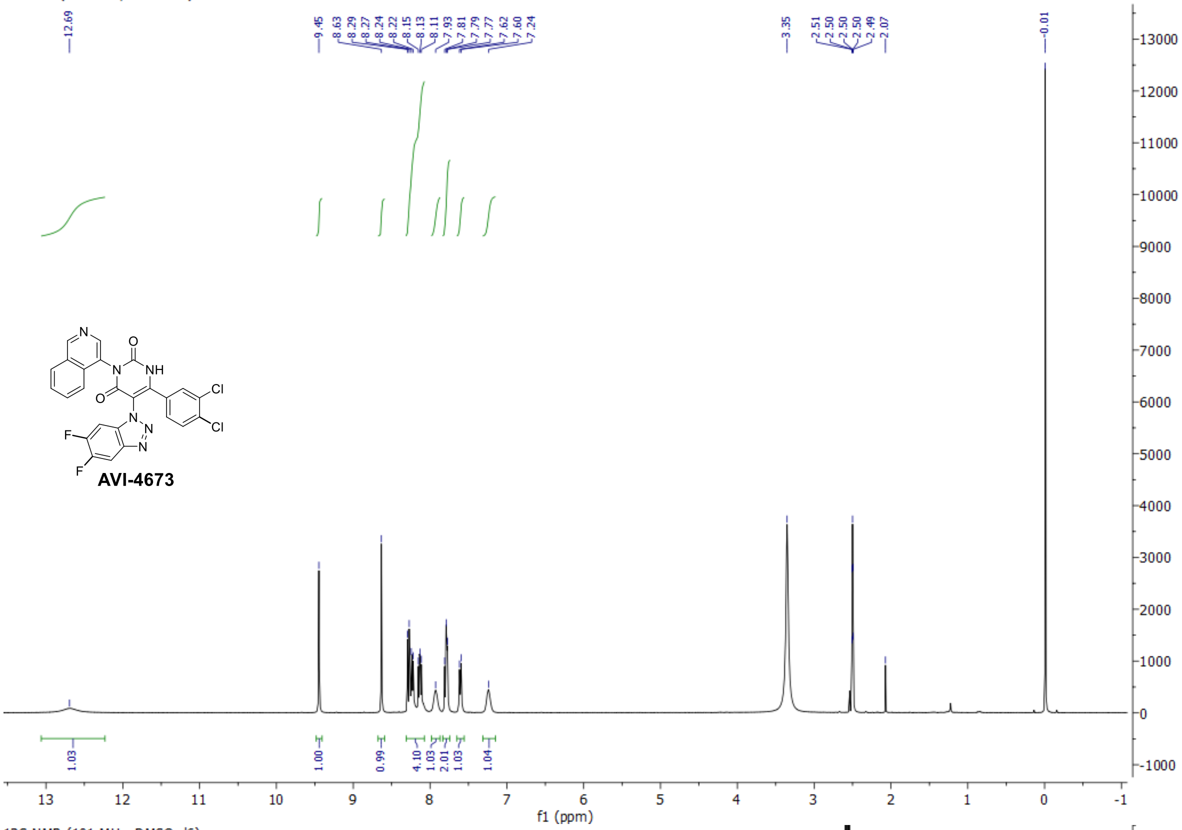


13C-NMR (101 MHz, DMSO-d6)

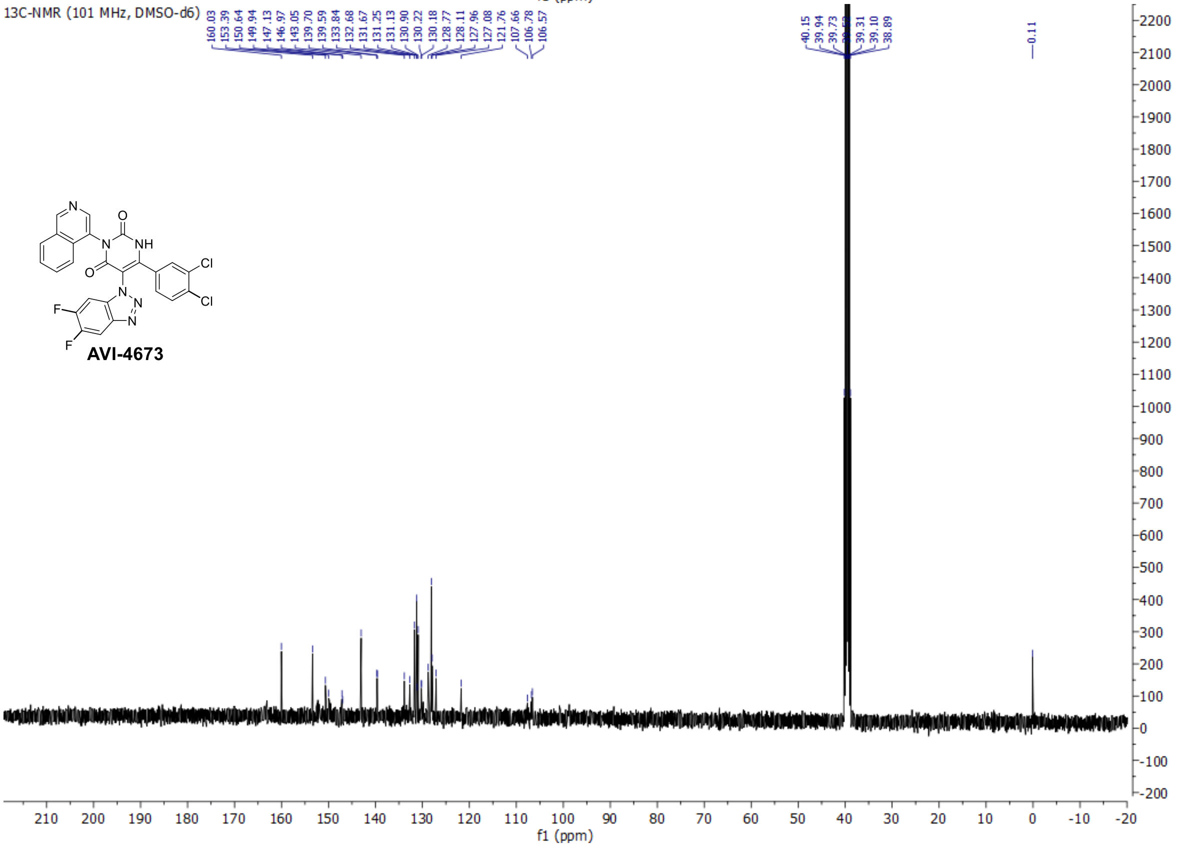

1H-NMR (400 MHz, DMSO-d6)

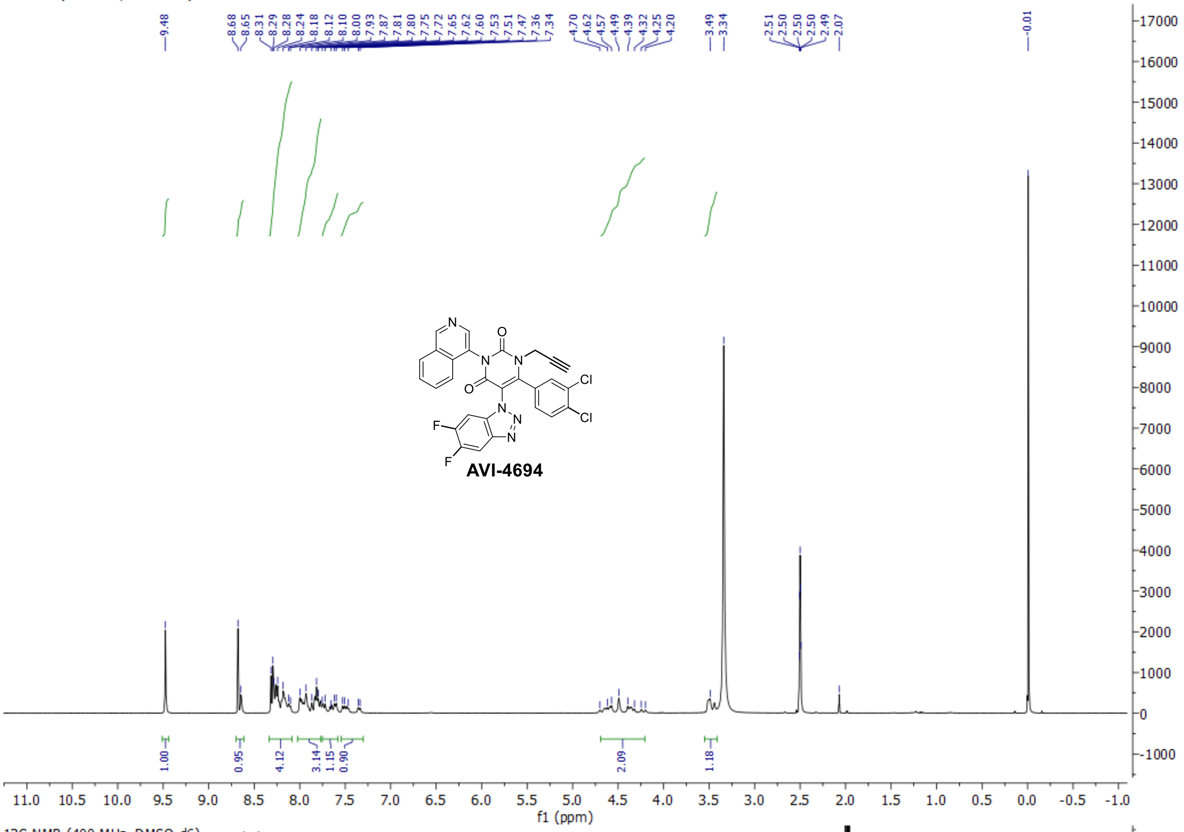


13C-NMR (400 MHz, DMSO-d6)

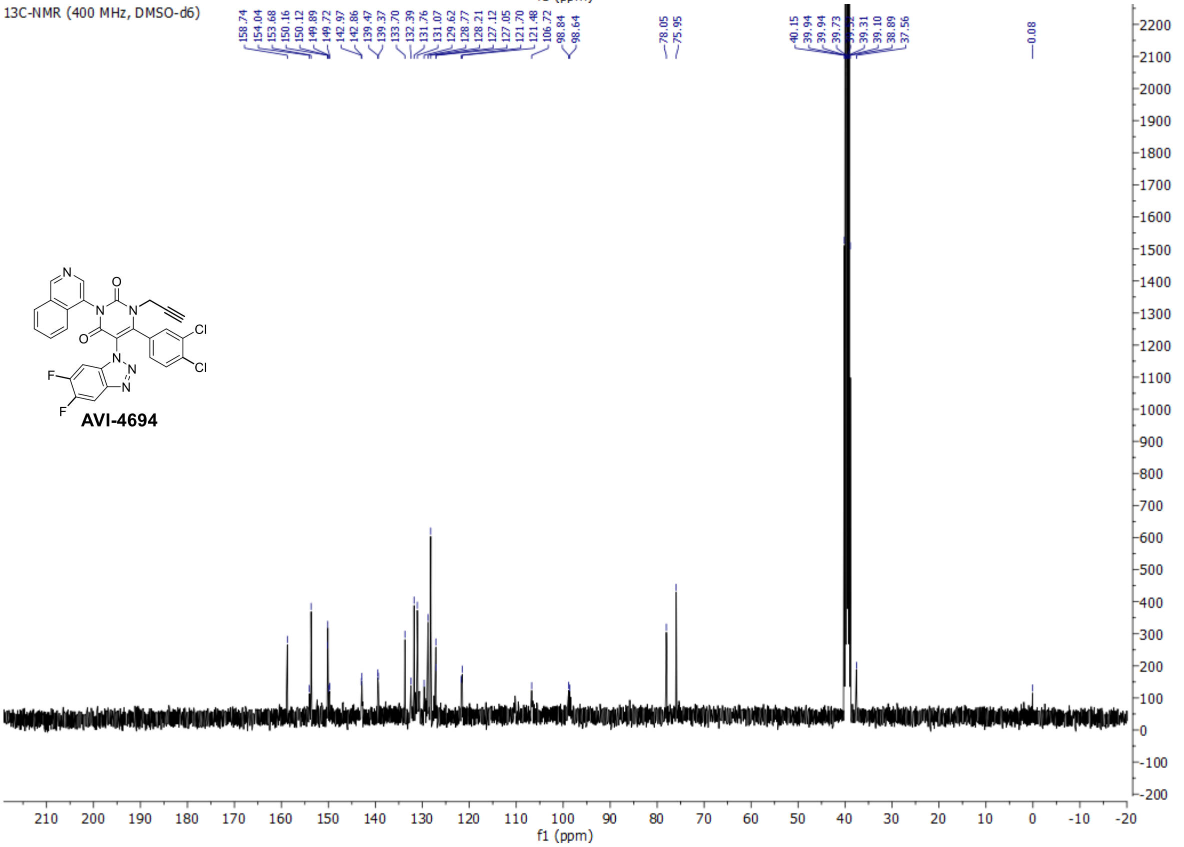

1H-NMR (400 MHz, DMSO-d6)

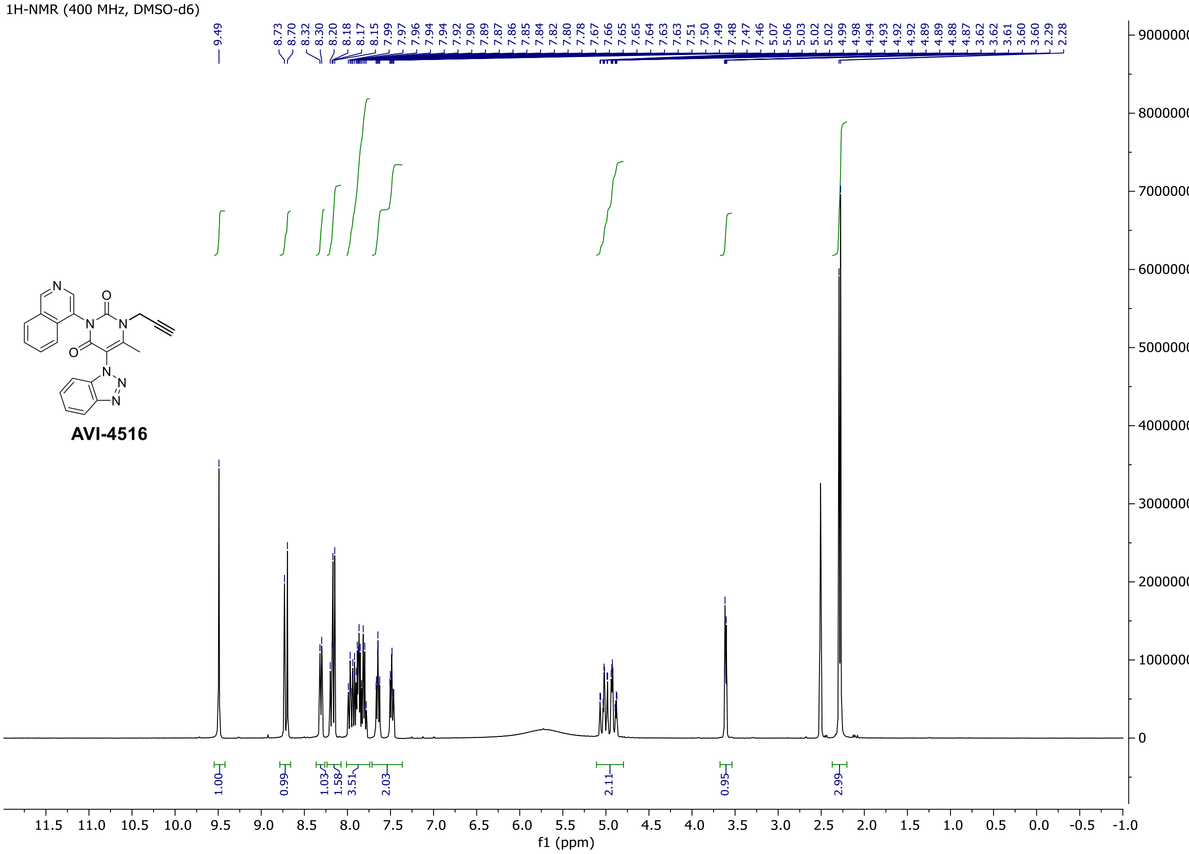


13C-NMR (101 MHz, DMSO-d6)

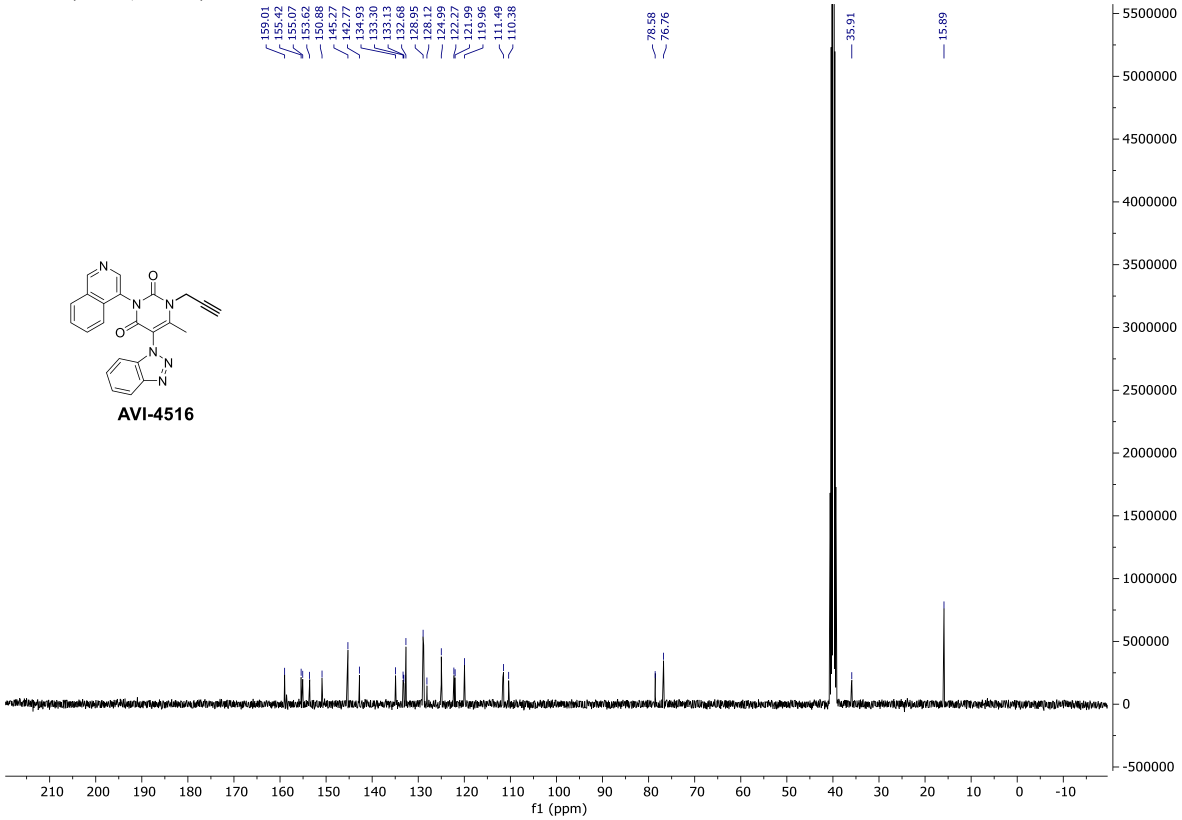

1H-NMR (400 MHz, DMSO-d6)

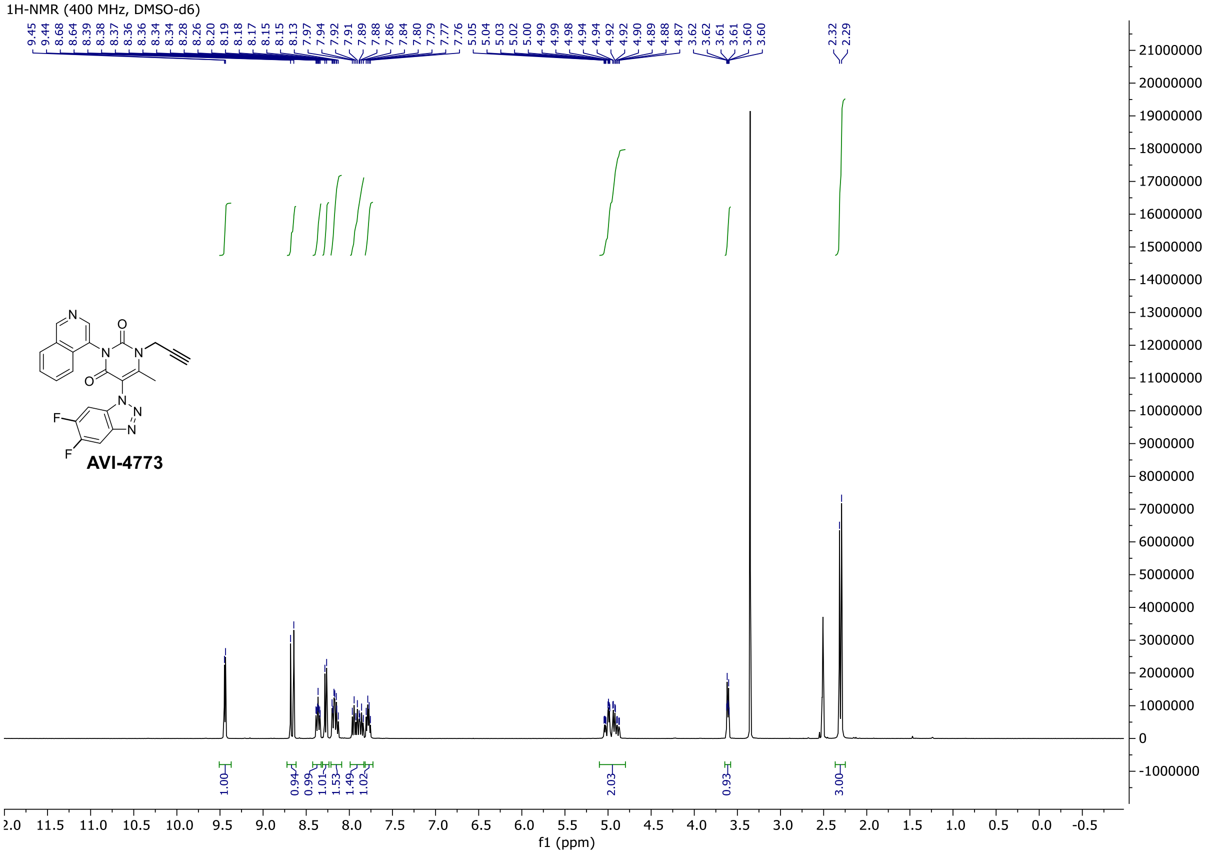


13C-NMR (101 MHz, DMSO-d6)

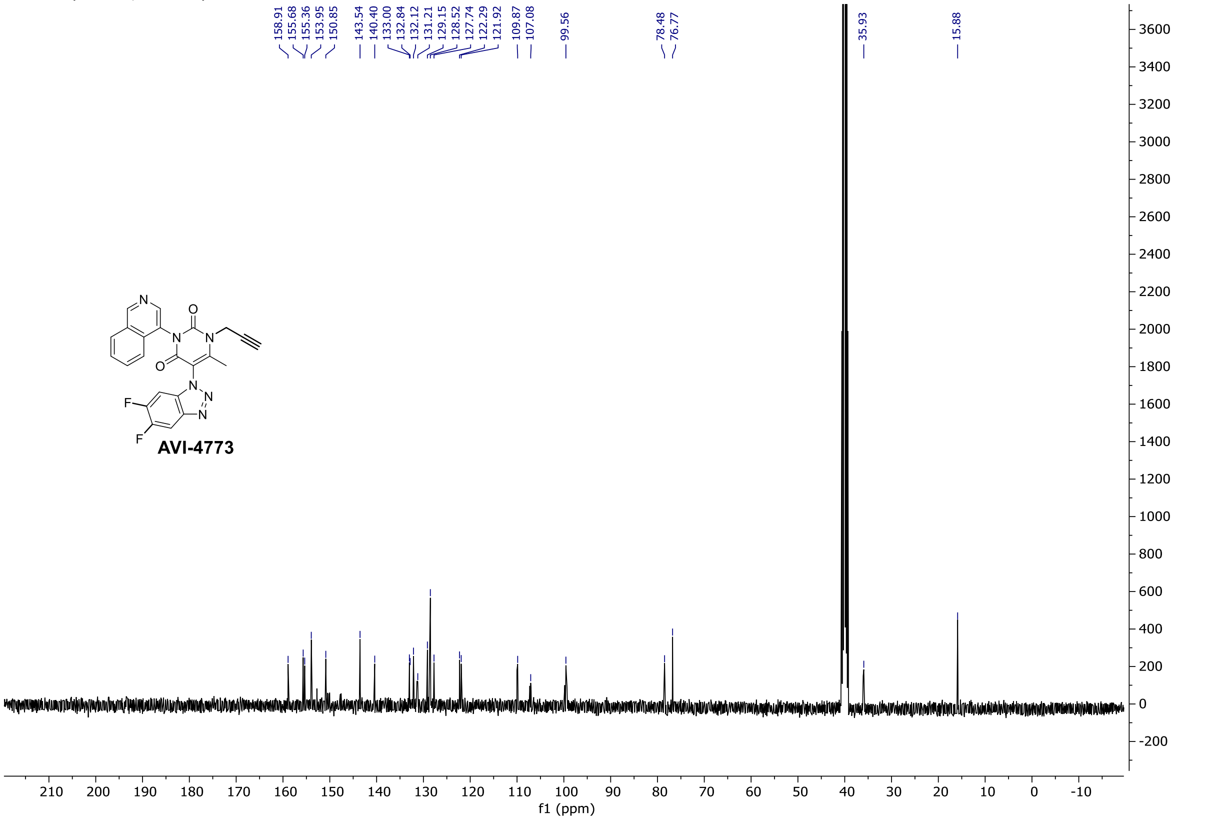

**Characterization data for Enamine Purchased compounds**

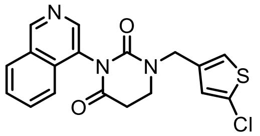


**Z8189030155**
**AVI-3778**

**$^1$H NMR** (500 MHz, DMSO-d6) δ 2.85 – 3.17 (m, 2H), 3.60 – 3.70 (m, 2H), 4.51 (s, 2H), 7.08 (s, 1H), 7.40 (s, 1H), 7.68 – 7.74 (m, 1H), 7.77 – 7.81 (m, 2H), 8.21 (d, *J* = 8.11 Hz, 1H), 8.40 (s, 1H), 9.33 (s, 1H).
**LC/MS** (ESI*) m/z 372.0 $[M+H]^+$
**HPLC** *System B*: $t_R$ = 1.038, purity: 96.97 % (215 nm)
**Method**: LCMS-29.

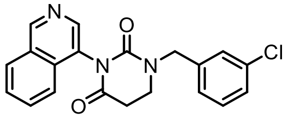


**Z8435235406**
**AVI-3779**

**$^1$H NMR** (500 MHz, DMSO-d6) δ 2.87 – 3.12 (m, 2H), 3.60 – 3.71 (m, 2H), 4.62 (s, 2H), 7.31 – 7.38 (m, 2H), 7.40 (d, *J* = 7.59 Hz, 1H), 7.42 – 7.45 (m, 1H), 7.70 – 7.74 (m, 1H), 7.76 – 7.84 (m, 2H), 8.21 (d, *J* = 8.13 Hz , 1H), 8.41 (s, 1H), 9.34 (s, 1H).
**LC/MS** (ESI*) m/z 366.0 $[M+H]^+$
**HPLC** *System B*: $t_R$ = 1.194, purity: 95.74 % (215 nm)
**Method**: LCMS-3.

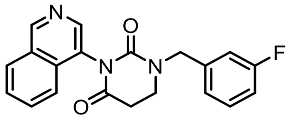


**Z8445588872**
**AVI-3780**

**$^1$H NMR** (500 MHz, DMSO-d6) δ 2.87 – 3.14 (m, 2H), 3.60 – 3.71 (m, 2H), 4.63 (s, 2H), 7.12 (t, J = 8.13 Hz, 1H), 7.18 – 7.21 (m, 1H), 7.21 – 7.24 (m, 1H), 7.38 – 7.46 (m, 1H), 7.72 (t, J = 7.03, 1H), 7.75 – 7.86 (m, 2H), 8.21 (d, J = 8.18 Hz, 1H), 8.41 (s, 1H), 9.34 (s, 1H).
**LC/MS** (ESI*) m/z 350.0 $[M+H]^+$
**HPLC** *System B*: $t_R$ = 0.968, purity: 97.13 % (215 nm)
**Method**: LCMS-29.

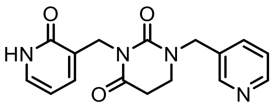


**Z3338086954**
**AVI-3436**

**$^1$H NMR** (500 MHz, DMSO-d6) δ 2.78 (t, *J* = 6.83 Hz, 2H), 3.45 (t, J = 6.73 Hz, 2H), 4.56 (s, 2H), 4.59 (s, 2H), 6.09 (t, *J* = 6.58 Hz, 1H), 6.96 (d, *J* = 6.69 Hz, 1H), 7.24 (d, J = 6.45 Hz, 1H), 7.36 (dd, *J* = 4.76, 7.90 Hz, 1H), 7.71 (d, *J* = 7.77 Hz, 1H), 8.48 (d, *J* = 4.72 Hz, 1H), 8.52 (s, 1H), 11.63 (br s, 1H).
**LC/MS** (ESI*) m/z 313.0 $[M+H]^+$

**HPLC** *System B*: $t_R$ = 0.588, purity: 95.53 % (215 nm)
**Method**: LCMS-1.

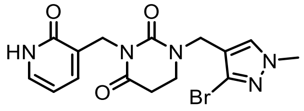


**Z3535311170**
**AVI-3443**

**¹H NMR** (500 MHz, DMSO-d6) δ 2.74 (t, *J* = 6.80 Hz, 2H), 3.38 (t, *J* = 6.70 Hz, 2H), 3.77 (s, 3H), 4.32 (s, 2H), 4.54 (s, 2H), 6.09 (t, *J* = 6.66 Hz, 1H), 6.90 (d, *J* = 6.69 Hz, 1H), 7.24 (d, *J* = 6.36 Hz, 1H), 7.73 (s, 1H), 11.63 (br s, 1H).
**LC/MS** (ESI*) m/z 394.0 $[M+H]^+$
**HPLC** *System B*: $t_R$ = 0.870, purity: 100 % (215 nm)
**Method**: LCMS-1.

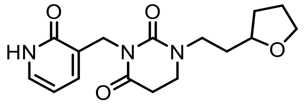


**Z3535316691**
**AVI-3441**

**¹H NMR** (500 MHz, DMSO-d6) δ 2.62 – 2.72 (m, 2H), 3.48 – 3.53 (m, 1H), 3.55 – 3.62 (m, 2H), 3.78 (dd, *J* = 3.25, 14.15 Hz, 1H), 4.44 – 4.56 (m, 2H), 4.96 – 5.10 (m, 2H), 5.35 – 5.40 (m, 1H), 6.07 (t, *J* = 6.57 Hz, 1H), 6.70 (d, *J* = 6.67 Hz, 1H), 7.21 – 7.26 (m, 2H), 7.27 – 7.29 (m, 1H), 7.29 – 7.35 (m, 2H), 11.62 (br s, 1H).
**LC/MS** (ESI*) m/z 354.0 $[M+H]^+$
**HPLC** *System B*: $t_R$ = 0.995, purity: 100 % (215 nm)
**Method**: LCMS-1.

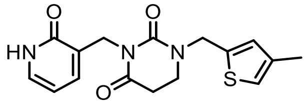


**Z3535311658**
**AVI-3319**

**¹H NMR** (500 MHz, DMSO-d6) δ 2.15 (s, 3H), 2.75 (t, *J* = 6.84 Hz, 2H), 3.41 (t, *J* = 6.82 Hz, 2H), 4.55 (s, 2H), 4.64 (s, 2H), 6.09 (t, *J* = 6.59 Hz, 1H), 6.82 – 6.91 (m, 2H), 7.00 (s, 1H), 7.24 (d, *J* = 6.47 Hz, 1H), 11.64 (br s, 1H).
**LC/MS** (ESI*) m/z 332.0 $[M+H]^+$
**HPLC** *System B*: $t_R$ = 1.054, purity: 94.30 % (215 nm)
**Method**: LCMS-1.

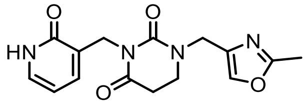


**Z3535313327**
**AVI-3437**

**¹H NMR** (500 MHz, DMSO-d6) δ 2.36 (s, 3H), 2.75 (t, *J* = 6.71 Hz, 2H), 3.47 (t, *J* = 6.69 Hz, 2H), 4.40 (s, 2H), 4.53 (s, 2H), 6.09 (t, *J* = 6.66 Hz, 1H), 6.93 (d, *J* = 6.84 Hz, 1H), 7.24 (d, *J* = 6.95 Hz, 1H), 7.89 (s, 1H), 11.63 (br s, 1H).
**LC/MS** (ESI*) m/z 317.0 $[M+H]^+$
**HPLC** *System B*: $t_R$ = 0.817, purity: 95.02 % (215 nm)
**Method**: LCMS-1.

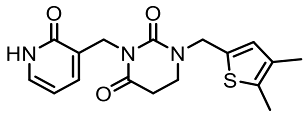

**Z3535313440**
**AVI-3440**

**$^{1}$H NMR** (500 MHz, DMSO-d6) δ 2.02 (s, 3H), 2.22 (s, 3H), 2.73 (t, *J* = 6.81 Hz, 2H), 3.38 (t, *J* = 6.80 Hz, 2H), 4.54 (s, 2H), 4.57 (s, 2H), 6.09 (t, *J* = 6.64 Hz, 1H), 6.73 (s, 1H), 6.88 (d, *J* = 6.69 Hz, 1H), 7.25 (d, *J* = 6.40 Hz, 1H), 11.64 (br s, 1H).
**LC/MS** (ESI*) m/z 346.0 $[M+H]^+$
**HPLC** *System B*: $t_R$ = 1.131, purity: 100 % (215 nm)
**Method**: LCMS-1.

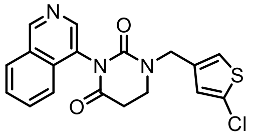

**Z8189030155**
**AVI-3778**

**$^{1}$H NMR** (500 MHz, DMSO-d6) δ 2.85 – 3.17 (m, 2H), 3.60 – 3.70 (m, 2H), 4.51 (s, 2H), 7.08 (s, 1H), 7.40 (s, 1H), 7.68 – 7.74 (m, 1H), 7.77 – 7.81 (m, 2H), 8.21 (d, *J* = 8.11 Hz, 1H), 8.40 (s, 1H), 9.33 (s, 1H).
**LC/MS** (ESI*) m/z 372.0 $[M+H]^+$
**HPLC** *System B*: $t_R$ = 1.038, purity: 96.97 % (215 nm)
**Method**: LCMS-29.

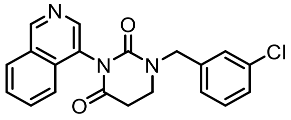

**Z8435235406**
**AVI-3779**

**$^{1}$H NMR** (500 MHz, DMSO-d6) δ 2.87 – 3.12 (m, 2H), 3.60 – 3.71 (m, 2H), 4.62 (s, 2H), 7.31 – 7.38 (m, 2H), 7.40 (d, *J* = 7.59 Hz, 1H), 7.42 – 7.45 (m, 1H), 7.70 – 7.74 (m, 1H), 7.76 – 7.84 (m, 2H), 8.21 (d, *J* = 8.13 Hz , 1H), 8.41 (s, 1H), 9.34 (s, 1H).
**LC/MS** (ESI*) m/z 366.0 $[M+H]^+$
**HPLC** *System B*: $t_R$ = 1.194, purity: 95.74 % (215 nm)
**Method**: LCMS-3.

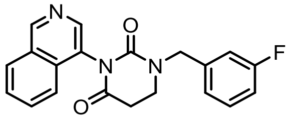

**Z8445588872**
**AVI-3780**

**$^{1}$H NMR** (500 MHz, DMSO-d6) δ 2.87 – 3.14 (m, 2H), 3.60 – 3.71 (m, 2H), 4.63 (s, 2H), 7.12 (t, J = 8.13 Hz, 1H), 7.18 – 7.21 (m, 1H), 7.21 – 7.24 (m, 1H), 7.38 – 7.46 (m, 1H), 7.72 (t, J = 7.03, 1H), 7.75 – 7.86 (m, 2H), 8.21 (d, J = 8.18 Hz, 1H), 8.41 (s, 1H), 9.34 (s, 1H).
**LC/MS** (ESI*) m/z 350.0 $[M+H]^+$
**HPLC** *System B*: $t_R$ = 0.968, purity: 97.13 % (215 nm)

**Method**: LCMS-29.

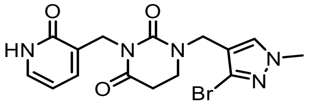

**Z3535311170**
**AVI-3443**

**$^1$H NMR** (500 MHz, DMSO-d6) δ 2.74 (t, *J* = 6.80 Hz, 2H), 3.38 (t, *J* = 6.70 Hz, 2H), 3.77 (s, 3H), 4.32 (s, 2H), 4.54 (s, 2H), 6.09 (t, *J* = 6.66 Hz, 1H), 6.90 (d, *J* = 6.69 Hz, 1H), 7.24 (d, *J* = 6.36 Hz, 1H), 7.73 (s, 1H), 11.63 (br s, 1H).
**LC/MS** (ESI*) m/z 394.0 $[M+H]^+$
**HPLC** *System B*: $t_R$ = 0.870, purity: 100 % (215 nm)
**Method**: LCMS-1.

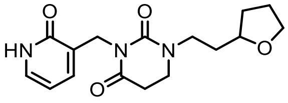

**Z3535316691**
**AVI-3441**

**$^1$H NMR** (500 MHz, DMSO-d6) δ 2.62 – 2.72 (m, 2H), 3.48 – 3.53 (m, 1H), 3.55 – 3.62 (m, 2H), 3.78 (dd, *J* = 3.25, 14.15 Hz, 1H), 4.44 – 4.56 (m, 2H), 4.96 – 5.10 (m, 2H), 5.35 – 5.40 (m, 1H), 6.07 (t, *J* = 6.57 Hz, 1H), 6.70 (d, *J* = 6.67 Hz, 1H), 7.21 – 7.26 (m, 2H), 7.27 – 7.29 (m, 1H), 7.29 – 7.35 (m, 2H), 11.62 (br s, 1H).
**LC/MS** (ESI*) m/z 354.0 $[M+H]^+$
**HPLC** *System B*: $t_R$ = 0.995, purity: 100 % (215 nm)
**Method**: LCMS-1.

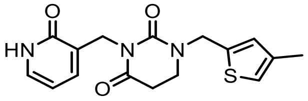

**Z3535311658**
**AVI-3319**

**$^1$H NMR** (500 MHz, DMSO-d6) δ 2.15 (s, 3H), 2.75 (t, *J* = 6.84 Hz, 2H), 3.41 (t, *J* = 6.82 Hz, 2H), 4.55 (s, 2H), 4.64 (s, 2H), 6.09 (t, *J* = 6.59 Hz, 1H), 6.82 – 6.91 (m, 2H), 7.00 (s, 1H), 7.24 (d, *J* = 6.47 Hz, 1H), 11.64 (br s, 1H).
**LC/MS** (ESI*) m/z 332.0 $[M+H]^+$
**HPLC** *System B*: $t_R$ = 1.054, purity: 94.30 % (215 nm)
**Method**: LCMS-1.

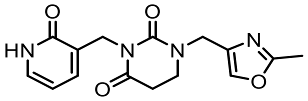

**Z3535313327**
**AVI-3437**

**$^1$H NMR** (500 MHz, DMSO-d6) δ 2.36 (s, 3H), 2.75 (t, *J* = 6.71 Hz, 2H), 3.47 (t, *J* = 6.69 Hz, 2H), 4.40 (s, 2H), 4.53 (s, 2H), 6.09 (t, *J* = 6.66 Hz, 1H), 6.93 (d, *J* = 6.84 Hz, 1H), 7.24 (d, *J* = 6.95 Hz, 1H), 7.89 (s, 1H), 11.63 (br s, 1H).
**LC/MS** (ESI*) m/z 317.0 $[M+H]^+$
**HPLC** *System B*: $t_R$ = 0.817, purity: 95.02 % (215 nm)
**Method**: LCMS-1.

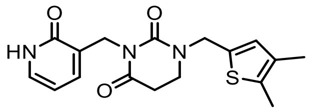

**Z3535313440**
**AVI-3440**

**$^1$H NMR** (500 MHz, DMSO-d6) δ 2.02 (s, 3H), 2.22 (s, 3H), 2.73 (t, *J* = 6.81 Hz, 2H), 3.38 (t, *J* = 6.80 Hz, 2H), 4.54 (s, 2H), 4.57 (s, 2H), 6.09 (t, *J* = 6.64 Hz, 1H), 6.73 (s, 1H), 6.88 (d, *J* = 6.69 Hz, 1H), 7.25 (d, *J* = 6.40 Hz, 1H), 11.64 (br s, 1H).
**LC/MS** (ESI*) m/z 346.0 $[M+H]^+$
**HPLC** *System B*: $t_R$ = 1.131, purity: 100 % (215 nm)
**Method**: LCMS-1.

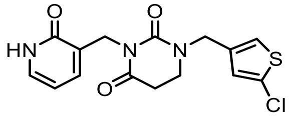

**Z8189030022**
**AVI-3750**

**$^1$H NMR** (500 MHz, DMSO-d6) ???
**LC/MS** (ESI*) m/z 352.2 $[M+H]^+$
**HPLC** *System B*: $t_R$ = 0.909, purity: 95 % (215 nm)
**Method**: LCMS-1.

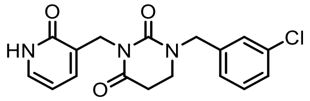

**Z8189030055**
**AVI-3993**

**$^1$H NMR** (500 MHz, DMSO-d6) δ 2.77 (t, J = 6.75 Hz, 2H), 3.42 (t, J = 6.86 Hz, 2H), 4.56 (s, 4H), 6.10 (t, J = 6.55 Hz, 1H), 6.96 (d, J = 6.70 Hz, 1H), 7.24 – 7.27 (m, 2H), 7.30 – 7.41 (m, 3H), 11.64 (br s, 1H).
**LC/MS** (ESI*) m/z 346.2 $[M+H]^+$
**HPLC** *System B*: $t_R$ = 0.915, purity: 92 % (215 nm)
**Method**: LCMS-1.

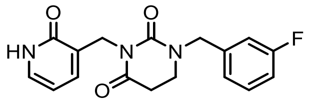

**Z8189030074**
**AVI-3992**

**$^1$H NMR** (500 MHz, DMSO-d6) δ 2.78 (t, J = 6.72 Hz, 2H), 3.42 (t, J = 6.74 Hz, 2H), 4.57 (s, 4H), 6.10 (t, J = 6.66 Hz, 1H), 6.97 (d, J = 6.83 Hz, 1H), 7.04 – 7.18 (m, 3H), 7.25 (d, J = 6.50 Hz, 1H), 7.33 – 7.41 (m, 1H), 11.64 (br s, 1H).
**LC/MS** (ESI*) m/z 330.2 $[M+H]^+$
**HPLC** *System B*: $t_R$ = 2.426, purity: 95 % (215 nm)
**Method**: LCMS-1.

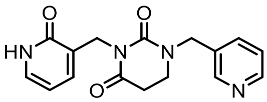

**Z3338086954**
**AVI-3436**

**$^1$H NMR** (500 MHz, DMSO-d6) δ 2.78 (t, J = 6.83 Hz, 2H), 3.45 (t, J = 6.73 Hz, 2H), 4.56 (s, 2H), 4.59 (s, 2H), 6.09 (t, J = 6.58 Hz, 1H), 6.96 (d, J = 6.69 Hz, 1H), 7.24 (d, J = 6.45 Hz, 1H), 7.36 (dd, J = 4.76, 7.90 Hz, 1H), 7.71 (d, J = 7.77 Hz, 1H), 8.48 (d, J = 4.72 Hz, 1H), 8.52 (s, 1H), 11.63 (br s, 1H).
**LC/MS** (ESI*) m/z 313 $[M+H]^+$
**HPLC** *System B*: $t_R$ = 0.588, purity: 96 % (215 nm)
**Method**: LCMS-1.

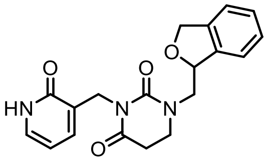

**Z3535311304**
**AVI-3431**

**$^1$H NMR** (500 MHz, DMSO-d6) δ 2.62 – 2.72 (m, 2H), 3.48 – 3.53 (m, 1H), 3.55 – 3.62 (m, 2H), 3.78 (dd, J = 3.25, 14.15 Hz, 1H), 4.44 – 4.56 (m, 2H), 4.96 – 5.10 (m, 2H), 5.35 – 5.40 (m, 1H), 6.07 (t, J = 6.57 Hz, 1H), 6.70 (d, J = 6.67 Hz, 1H), 7.21 – 7.26 (m, 2H), 7.27 – 7.29 (m, 1H), 7.29 – 7.35 (m, 2H), 11.62 (br s, 1H).
**LC/MS** (ESI*) m/z 354 $[M+H]^+$
**HPLC** *System B*: $t_R$ = 0.995, purity: 100 % (215 nm)
**Method**: LCMS-1.

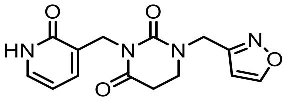

**Z3535314312**
**AVI-3433**

**$^1$H NMR** (500 MHz, DMSO-d6) δ 2.79 (t, J = 6.72 Hz, 2H), 3.50 (t, J = 6.77 Hz, 2H), 4.55 (s, 2H), 4.66 (s, 2H), 6.09 (t, J = 6.65 Hz, 1H), 6.47 – 6.61 (m, 1H), 6.95 (d, J = 6.79 Hz, 1H), 7.24 (d, J = 6.49 Hz, 1H), 8.66 – 9.06 (m, 1H), 11.64 (br s, 1H).
**LC/MS** (ESI*) m/z 303.1 $[M+H]^+$
**HPLC** *System B*: $t_R$ = 0.789, purity: 100 % (215 nm)
**Method**: LCMS-1.

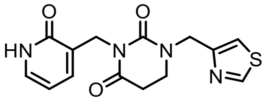

**Z3535315305**
**AVI-3428**

**$^1$H NMR** (500 MHz, DMSO-d6) δ 2.78 (t, J = 6.78 Hz, 2H), 3.52 (t, J = 6.85 Hz, 2H), 4.55 (s, 2H), 4.69 (s, 2H), 6.10 (t, J = 6.69 Hz, 1H), 6.95 (d, J = 6.76 Hz, 1H), 7.24 (d, J = 6.53 Hz, 1H), 7.60 (s, 1H), 9.07 – 9.11 (m, 1H), 11.63 (br s, 1H).
**LC/MS** (ESI*) m/z 319 $[M+H]^+$
**HPLC** *System B*: $t_R$ = 0.813, purity: 90 % (215 nm)
**Method**: LCMS-1.

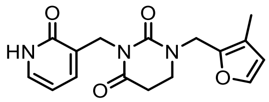

**Z3535315436**
**AVI-3429**

**$^1$H NMR** (500 MHz, DMSO-d6) δ 1.98 (s, 3H), 2.72 (t, J = 6.86 Hz, 2H), 3.37 (t, J = 6.86 Hz, 2H), 4.51 (s, 2H), 4.53 (s, 2H), 6.09 (t, J = 6.65 Hz, 1H), 6.23 – 6.39 (m, 1H), 6.87 (d, J = 6.82 Hz, 1H), 7.24 (d, J = 6.32 Hz, 1H), 7.44 – 7.55 (m, 1H), 11.64 (br s, 1H).
**LC/MS** (ESI*) m/z 316.2 $[M+H]^+$
**HPLC** *System B*: $t_R$ = 1.015, purity: 100 % (215 nm)
**Method**: LCMS-1.

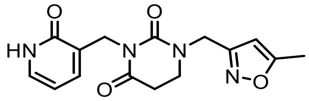

**Z3535316272**
**AVI-3439**

**$^1$H NMR** (500 MHz, DMSO-d6) δ 2.36 (s, 3H), 2.77 (t, J = 6.74 Hz, 2H), 3.47 (t, J = 6.75 Hz, 2H), 4.55 (s, 2H), 4.56 (s, 2H), 6.09 (t, J = 6.66 Hz, 1H), 6.17 (s, 1H), 6.93 (d, J = 6.78 Hz, 1H), 7.25 (d, J = 6.46 Hz, 1H), 11.63 (br s, 1H).
**LC/MS** (ESI*) m/z 317.2 $[M+H]^+$
**HPLC** *System B*: $t_R$ = 0.705, purity: 97 % (215 nm)
**Method**: LCMS-1.

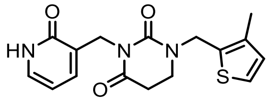

**Z3535316611**
**AVI-3425**

**$^1$H NMR** (500 MHz, DMSO-d6) δ 2.18 (s, 3H), 2.73 (t, J = 6.89 Hz, 2H), 3.39 (t, J = 6.77 Hz, 2H), 4.55 (s, 2H), 4.66 (s, 2H), 6.09 (t, J = 6.67 Hz, 1H), 6.84 (d, J = 5.08 Hz, 1H), 6.88 (d, J = 6.84 Hz, 1H), 7.24 (d, J = 6.44 Hz, 1H), 7.34 (d, J = 5.07 Hz, 1H), 11.64 (br s, 1H).
**LC/MS** (ESI*) m/z 332.1 $[M+H]^+$
**HPLC** *System B*: $t_R$ = 1.068, purity: 100 % (215 nm)
**Method**: LCMS-1.

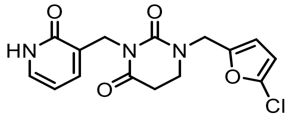

**Z3535316979**
**AVI-3432**

**$^1$H NMR** (500 MHz, DMSO-d6) δ 2.76 (t, J = 6.72 Hz, 2H), 3.44 (t, J = 6.82 Hz, 2H), 4.53 (s, 2H), 4.54 (s, 2H), 6.09 (t, J = 6.69 Hz, 1H), 6.42 (d, J = 3.36 Hz, 1H), 6.49 (d, J = 3.46 Hz, 1H), 6.91 (d, J = 6.66 Hz, 1H), 7.24 (d, J = 6.44 Hz, 1H), 11.64 (br s, 1H).
**LC/MS** (ESI*) m/z 336 $[M+H]^+$
**HPLC** *System B*: $t_R$ = 1.021, purity: 96 % (215 nm)
**Method**: LCMS-1.

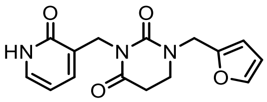

**Z3535317000**
**AVI-3444**

**$^1$H NMR** (500 MHz, DMSO-d6) δ 2.75 (t, J = 6.69 Hz, 2H), 3.42 (t, J = 6.62 Hz, 2H), 4.54 (s, 2H), 4.55 (s, 2H), 6.09 (t, J = 6.67 Hz, 1H), 6.33 – 6.38 (m, 1H), 6.38 – 6.45 (m, 1H), 6.90 (d, J = 6.70 Hz, 1H), 7.24 (d, J = 6.51 Hz, 1H), 7.53 – 7.71 (m, 1H), 11.64 (br s, 1H).
**LC/MS** (ESI*) m/z 302.1 [M+H]$^+$
**HPLC** *System B*: $t_R$ = 0.925, purity: 100 % (215 nm)
**Method**: LCMS-1.

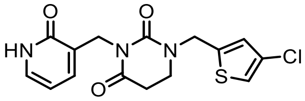

**Z3638153574**
**AVI-3318**

**$^1$H NMR** (500 MHz, DMSO-d6) δ 2.77 (t, J = 6.73 Hz, 2H), 3.45 (t, J = 6.82 Hz, 2H), 4.55 (s, 2H), 4.67 (s, 2H), 6.08 (t, J = 6.58 Hz, 1H), 6.90 (d, J = 6.75 Hz, 1H), 7.07 (s, 1H), 7.24 (d, J = 6.33 Hz, 1H), 7.48 (s, 1H), 11.64 (br s, 1H).
**LC/MS** (ESI*) m/z 351.9 [M+H]$^+$
**HPLC** *System B*: $t_R$ = 1.072, purity: 100 % (215 nm)
**Method**: LCMS-1.

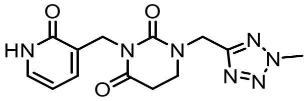

**Z7160954739**
**AVI-3435**

**$^1$H NMR** (500 MHz, DMSO-d6) δ 2.80 (t, J = 6.83 Hz, 2H), 3.59 (t, J = 6.78 Hz, 2H), 4.33 (s, 3H), 4.54 (s, 2H), 4.83 (s, 2H), 6.10 (t, J = 6.60 Hz, 1H), 6.93 (d, J = 6.76 Hz, 1H), 7.24 (d, J = 6.43 Hz, 1H), 11.64 (br s, 1H).
**LC/MS** (ESI*) m/z 318.2 [M+H]$^+$
**HPLC** *System B*: $t_R$ = 0.736, purity: 98 % (215 nm)
**Method**: LCMS-1.

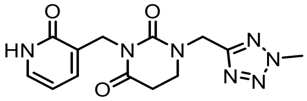

**Z7160954739**
**AVI-3435**

**$^1$H NMR** (500 MHz, DMSO-d6) δ 2.80 (t, J = 6.83 Hz, 2H), 3.59 (t, J = 6.78 Hz, 2H), 4.33 (s, 3H), 4.54 (s, 2H), 4.83 (s, 2H), 6.10 (t, J = 6.60 Hz, 1H), 6.93 (d, J = 6.76 Hz, 1H), 7.24 (d, J = 6.43 Hz, 1H), 11.64 (br s, 1H).
**LC/MS** (ESI*) m/z 318.2 [M+H]$^+$
**HPLC** *System B*: $t_R$ = 0.736, purity: 98 % (215 nm)
**Method**: LCMS-1.

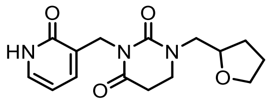

**Z3338086960**
**AVI-3420**

**$^1$H NMR** (500 MHz, DMSO-d6) δ 1.45 – 1.51 (m, 1H), 1.75 – 1.91 (m, 3H), 2.72 (t, J = 6.69 Hz, 2H), 3.26 – 3.29 (m, 1H), 3.45 – 3.57 (m, 3H), 3.59 – 3.64 (m, 1H), 3.71 – 3.76 (m, 1H), 3.94 – 4.00 (m, 1H), 4.52 (s, 2H), 6.09 (t, J = 6.66 Hz, 1H), 6.89 (d, J = 6.78 Hz, 1H), 7.24 (d, J = 6.55 Hz, 1H), 11.62 (br s, 1H).
**LC/MS** (ESI*) m/z 306.1 $[M+H]^+$
**HPLC** *System B*: $t_R$ = 0.854, purity: 100 % (215 nm)
**Method**: LCMS-1.

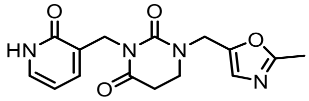

**Z3535313803**
**AVI-3422**

**$^1$H NMR** (500 MHz, DMSO-d6) δ 2.35 (s, 3H), 2.76 (t, J = 6.79 Hz, 2H), 3.44 (t, J = 6.81 Hz, 2H), 4.54 (s, 2H), 4.57 (s, 2H), 6.09 (t, J = 6.60 Hz, 1H), 6.92 (d, J = 6.77 Hz, 1H), 6.96 (s, 1H), 7.24 (d, J = 6.60 Hz, 1H), 11.63 (br s, 1H).
**LC/MS** (ESI*) m/z 317.1 $[M+H]^+$
**HPLC** *System B*: $t_R$ = 0.804, purity: 100 % (215 nm)
**Method**: LCMS-1.

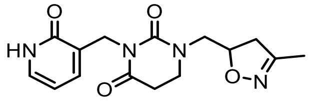

**Z3535314281**
**AVI-3423**

**$^1$H NMR** (500 MHz, DMSO-d6) δ 1.88 (s, 3H), 2.66 – 2.70 (m, 1H), 2.70 – 2.74 (m, 2H), 3.02 (dd, J = 10.51, 17.41 Hz, 1H), 3.39 – 3.44 (m, 1H), 3.48 – 3.61 (m, 3H), 4.53 (s, 2H), 4.62 – 4.69 (m, 1H), 6.09 (t, J = 6.63 Hz, 1H), 6.91 (d, J = 6.67 Hz, 1H), 7.24 (d, J = 6.43 Hz, 1H), 11.63 (br s, 1H).
**LC/MS** (ESI*) m/z 319 $[M+H]^+$
**HPLC** *System B*: $t_R$ = 0.782, purity: 100 % (215 nm)
**Method**: LCMS-1.

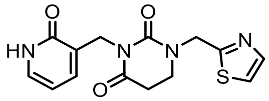

**Z3535315936**
**AVI-3418**

**$^1$H NMR** (500 MHz, DMSO-d6) δ 2.81 (t, J = 6.77 Hz, 2H), 3.58 (t, J = 6.79 Hz, 2H), 4.56 (s, 2H), 4.86 (s, 2H), 6.10 (t, J = 6.69 Hz, 1H), 6.94 (d, J = 6.74 Hz, 1H), 7.25 (d, J = 6.44 Hz, 1H), 7.69 (d, J = 3.37 Hz, 1H), 7.76 (d, J = 3.26 Hz, 1H), 11.64 (br s, 1H).
**LC/MS** (ESI*) m/z 319 $[M+H]^+$
**HPLC** *System B*: $t_R$ = 0.825, purity: 93 % (215 nm)
**Method**: LCMS-1.

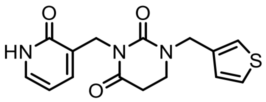

**Z3535316454**
**AVI-3320**

**$^1$H NMR** (500 MHz, DMSO-d6) δ 2.75 (t, J = 6.86 Hz, 2H), 3.40 (t, J = 6.80 Hz, 2H), 4.54 (s, 2H), 4.56 (s, 2H), 6.09 (t, J = 6.56 Hz, 1H), 6.93 (d, J = 6.68 Hz, 1H), 7.02 (d, J = 4.97 Hz, 1H), 7.24 (d, J = 6.55 Hz, 1H), 7.41 (s, 1H), 7.47 – 7.55 (m, 1H), 11.63 (br s, 1H).
**LC/MS** (ESI*) m/z 318.1 $[M+H]^+$
**HPLC** *System B*: $t_R$ = 0.989, purity: 100 % (215 nm)
**Method**: LCMS-1.

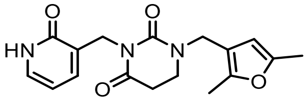

**Z3638153760**
**AVI-3416**

**$^1$H NMR** (500 MHz, DMSO-d6) δ ???
**LC/MS** (ESI*) m/z 330.2 $[M+H]^+$
**HPLC** *System B*: $t_R$ = 0.866, purity: 100 % (215 nm)
**Method**: LCMS-1.

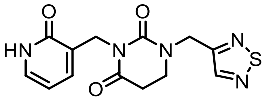

**Z4349493904**
**AVI-3419**

**$^1$H NMR** (500 MHz, DMSO-d6) δ 2.84 (t, J = 6.71 Hz, 2H), 3.61 (t, J = 6.77 Hz, 2H), 4.55 (s, 2H), 4.88 (s, 2H), 6.10 (t, J = 6.63 Hz, 1H), 6.98 (d, J = 6.72 Hz, 1H), 7.24 (d, J = 6.42 Hz, 1H), 8.81 (s, 1H), 11.64 (br s, 1H).
**LC/MS** (ESI*) m/z 320 $[M+H]^+$
**HPLC** *System B*: $t_R$ = 0.822, purity: 99 % (215 nm)
**Method**: LCMS-1.

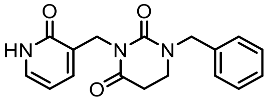

**Z7160953946**
**AVI-3321**

**$^1$H NMR** (500 MHz, DMSO-d6) δ 2.75 (t, J = 6.71 Hz, 2H), 3.38 (t, J = 6.72 Hz, 2H), 4.56 (s, 2H), 4.57 (s, 2H), 6.10 (t, J = 6.69 Hz, 1H), 6.95 (d, J = 6.64 Hz, 1H), 7.22 – 7.30 (m, 4H), 7.31 – 7.37 (m, 2H), 11.64 (br s, 1H).
**LC/MS** (ESI*) m/z 312.2 $[M+H]^+$
**HPLC** *System B*: $t_R$ = 1.003, purity: 100 % (215 nm)
**Method**: LCMS-1.

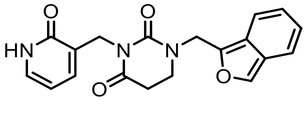

**Z7160954690**
**AVI-3415**

**$^1$H NMR** (500 MHz, DMSO-d6) δ 2.72 (t, J = 6.79 Hz, 2H), 3.39 (t, J = 6.81 Hz, 2H), 4.59 (s, 2H), 4.69 (s, 2H), 6.06 (t, J = 6.60 Hz, 1H), 6.90 (d, J = 6.69 Hz, 1H), 7.20 (t, J = 7.53 Hz, 1H), 7.26 (d, J = 6.78 Hz, 1H), 7.31 (t, J = 7.74 Hz, 1H), 7.57 (t, J = 8.24 Hz, 2H), 8.03 (s, 1H), 11.65 (br s, 1H).
**LC/MS** (ESI*) m/z 352 $[M+H]^+$
**HPLC** *System B*: $t_R$ = 1.086, purity: 100 % (215 nm)
**Method**: LCMS-1.

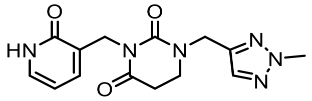


**Z7160954841**
**AVI-3424**

**$^1$H NMR** (500 MHz, DMSO-d6) δ 2.76 (t, J = 6.85 Hz, 2H), 3.47 (t, J = 6.83 Hz, 2H), 4.09 (s, 3H), 4.54 (s, 2H), 4.59 (s, 2H), 6.09 (t, J = 6.61 Hz, 1H), 6.93 (d, J = 6.78 Hz, 1H), 7.24 (d, J = 6.50 Hz, 1H), 7.65 (s, 1H), 11.63 (br s, 1H).
**LC/MS** (ESI*) m/z 317.1 $[M+H]^+$
**HPLC** *System B*: $t_R$ = 0.796, purity: 97 % (215 nm)
**Method**: LCMS-1.
